# Supplementary material for: Lithium hexamethyldisilazide initiated superfast ring opening polymerization of alpha-amino acid N-carboxyanhydrides
Source: Nat Commun. 2018 Dec 13;9:5297. doi: 10.1038/s41467-018-07711-y (PMC6294000; doi:10.1038/s41467-018-07711-y)
Supplement: Supplementary file 1 — Supplementary Information [file 41467_2018_7711_MOESM1_ESM.pdf]

Supplementary Information

Lithium Hexamethyldisilazide Initiated Superfast Ring Opening Polymerization of Alpha-Amino Acid *N*-Carboxyanhydrides

Y. Wu *et al.*

Supplementary Methods

Materials. Anhydrous tetrahydrofuran (THF), Anhydrous N,N-dimethylformamide (DMF), lithium hexamethyldisilazide (LiHMDS), n-hexylamine and 1,1,1,3,3,3-hexamethyldisilazane (HMDS) were purchased from Sigma-Aldrich and used without further purification; other commercial sources LiHMDS were purchased from Beijing HWRK Chem and Shanghai Adamas Reagent; Triphosgene was purchased from Beijing InnoChem Science and Technology Co., Ltd; L-Glutamic acid γ-benzyl ester was purchased from Shanghai Aladdin Bio-Chem Technology Co., Ltd; D,L-Lysine, L-leucine and 2,2'-bipyridine were purchased from Shanghai D&B Biological Science and Technology Co., Ltd; Nε-tert-butyloxycarbonyl-L-lysine and L-aspartic acid β-tert-butyl ester were purchased from Bide Pharmatech Ltd; α-pinene was purchased from J&K Scientific Ltd; bis(1,5-cyclooctadiene)nickel(0) ($Ni(COD)_2$) was purchased from Sinocompound Catalysts Co., Ltd; H-L-Orn(Boc)-OH, O-(tert-butyl)-L-serine, ethyl acetate (EtOAc), hexane and other solvents and reagents were purchased from Shanghai Adamas Reagent; all solvents used in *N*-carboxyanhydride (NCA) purification were either freshly distilled (for THF) or dried over $MgSO_4$ (for EtOAc and Hexane); *Staphylococcus aureus USA300* (methicillin-resistant), *Staphylococcus aureus USA300 LAC* (methicillin-resistant), and *Staphylococcus aureus Mu50* (methicillin-resistant), *Bacillus subtilis BR-151*, *Escherichia coli JM109*, *Pseudomonas aeruginosa ATCC9027* (intermediate resistance to carbenicillin and piperacillin), *Pseudomonas aeruginosa ATCC15442* (multidrug resistance), *Pseudomonas aeruginosa O1* (naturally sulfamethoxazole and tetracycline-resistant), *Acinetobacter baumannii*

ATCC BAA-747, were used for antimicrobial study. Synthesized intermediates were purified using a SepaBean machine equipped with Sepaflash columns produced by Santai Technologies Inc. in China.

Synthesis of γ-benzyl-α-L-glutamate NCA (BLG NCA)

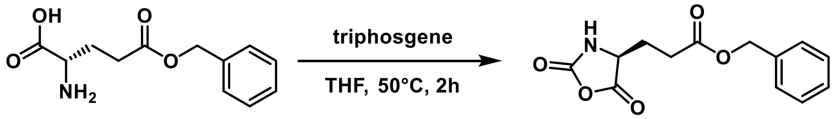

BLG NCA

BLG NCA was prepared by following the precedent literature with modifications[1]. To a solution of L-glutamic acid γ-benzyl ester (11.8 g, 50 mmol) in anhydrous THF (0.3 M) in a round bottom flask under ice-water bath, was added a solution of triphosgene (6.5 g, 22 mmol) in anhydrous THF under the protection of N_2. The reaction was stirred under N_2 protection at 50 °C for 2 hours, and then the solvent was removed under reduced pressure. The reaction mixture was dissolved in EtOAc (100 mL) and washed with cold water (100 mL) and cold brine (100 mL) successively. The collected organic layer was dried over anhydrous $MgSO_4$ and concentrated under reduced pressure to give a crude NCA. The crude product was purified by recrystallization three times in a glovebox with N_2 protection using a mixture of dried EtOAc and hexane to afford a colorless crystal 10.5 g (80% yield). ^{1}H NMR (400 MHz, $CDCl_3$, Supplementary Figure 8): δ 7.42–7.31 (m, 5H), 6.51 (s, 1H), 5.14 (s, 2H), 4.38 (t, J = 5.9 Hz, 1H), 2.60 (t, J = 6.8 Hz,

2H), 2.33–2.22 (m, 1H), 2.18-2.06 (m, 1H). ^{13}C NMR (100 MHz, $CDCl_3$, Supplementary Figure 9): δ 172.56, 169.46, 151.83, 135.32, 128.86, 128.76, 128.53, 67.28, 57.12, 30.06, 27.07.

Synthesis of Nε-*tert*-butyloxycarbonyl-α-D,L-lysine (H-D,L-Lys(Boc)-OH)

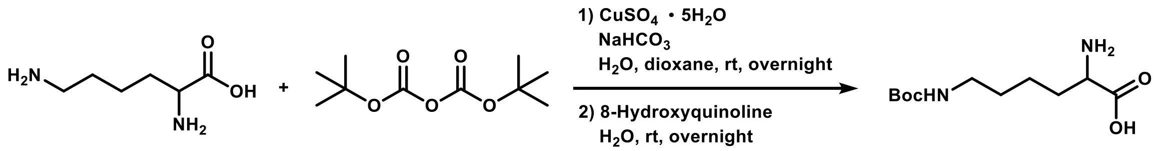

H-D,L-Lys(Boc)-OH was synthesized by following the previously reported procedure with slight modification.[2] To 250 mL aqueous solution of D,L-lysine (18.3 g, 125 mmol) supplemented with 1 M $NaHCO_3$ in a 1L round bottom flask, was slowly added $CuSO_4 \cdot 5H_2O$ (15.6 g, 62.5 mmol) and $NaHCO_3$ (10.5 g, 125 mmol) successively. Di-tert-butyl dicarbonate (35.4 g, 162.5 mmol) in dioxane (150 mL) was added to the flask and the reaction was stirred overnight at room temperature. MeOH (36 mL) was added to the flask and the reaction was stirred for another 2 hours, followed by addition of H_2O (125 mL) and EtOAc (125 mL). The blue solid was collected from filtration and then was suspended in H_2O (500 mL) under vigorously stir, followed by addition of 8-quinolinol (23.5 g, 162.5 mmol). The mixture was stirred overnight and then the green suspension was filtered, with the solid washed with H_2O. The filtrate was collected and washed with CH_2Cl_2 (3×500 mL), and the aqueous layer was concentrated under reduced pressure followed by an extra purification by reverse phase column chromatography to give H-D,L-Lys(Boc)-OH as a white solid 24 g (78% yield). ^{1}H NMR (400 MHz, D_2O, Supplementary Figure 10): δ 3.67 (t, J = 6.1 Hz, 1H), 3.04 (t, J = 6.8 Hz, 2H), 1.87–

1.76 (m, 2H), 1.52–1.44 (m, 2H), 1.39–1.34 (m, 11H). ^{13}C NMR (100 MHz, D_2O, Supplementary Figure 11): δ 174.92, 158.32, 80.84, 54.69, 39.65, 30.16, 28.59, 27.65, 21.64.

Synthesis of Nε-*tert*-butyloxycarbonyl-D,L-lysine NCA (Boc-D,L-Lys NCA)

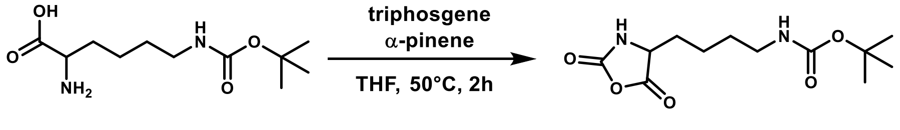

Boc-D,L-Lys NCA was prepared by following a previously reported method with slight modification.[3, 4] To a solution of H-D,L-Lys(Boc)-OH (12.3 g, 50 mmol) and α-pinene (20.5 mL, 132 mmol) in anhydrous THF (0.3 M) in a round bottom flask, was added a solution of triphosgene (6.5 g, 22 mmol) in anhydrous THF under N_2 cooled with an ice-water bath. The reaction was stirred under N_2 at 50 °C for 2 hours, and then the solvent was removed under reduced pressure. The reaction mixture was dissolved in EtOAc (100 mL) and washed with ice-water (100 mL) and ice-brine (100 mL). The collected organic layer was dried over anhydrous $MgSO_4$ and concentrated under reduced pressure to give a crude NCA. The crude NCA was purified by recrystallization in glovebox three times using a mixture of dry EtOAc and hexane to give a white powder 7.2 g (53% yield). ^1H NMR (400 MHz, $CDCl_3$, Supplementary Figure 12): δ 7.33 (br, 1H), 4.70 (br, 1H), 4.33 (dd, J = 6.7, 5.0 Hz, 1H), 3.12 (s, 2H), 2.05-1.91 (m, 1H), 1.89–1.77 (m, 1H), 1.61–1.47 (m, 4H), 1.43 (s, 9H). ^{13}C NMR (100 MHz, $CDCl_3$, Supplementary Figure 13): δ 170.04, 156.77, 152.36, 80.02, 57.63, 39.74, 30.94, 29.38, 28.57, 21.38.

Synthesis of Nε-*tert*-butyloxycarbonyl-L-lysine NCA (Boc-L-Lys NCA)

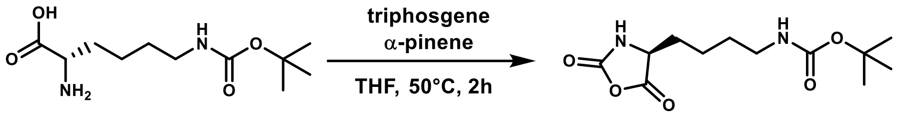

Boc-L-Lys NCA

Boc-L-Lys NCA was prepared using a procedure identical to that for Boc-D,L-Lys NCA mentioned above.[3, 4] ^{1}H NMR (400 MHz, $CDCl_3$, Supplementary Figure 14): δ 6.86 (br, 1H), 4.67 (br, 1H), 4.33 (t, *J* = 5.4 Hz, 1H), 3.13 (s, 2H), 2.10-1.95 (m, 1H), 1.91-1.77 (m, 1H), 1.63-1.49 (m, 4H), 1.45 (s, 9H).

Synthesis of Nδ-*tert*-butyloxycarbonyl-L-ornithine NCA (Boc-L-Orn NCA)

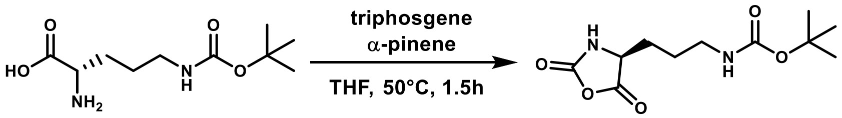

Boc-L-Orn NCA

Boc-L-Orn NCA[5] was prepared as a white crystal in 60% yield, from H-L-Orn(Boc)-OH using a similar method described above for Boc-D,L-Lys NCA synthesis. ^{1}H NMR (400 MHz, CD_3CN, Supplementary Figure 15): *δ* 6.92 (s, 1H), 5.36 (s, 1H), 4.33 (t, *J* = 5.77 Hz, 1H), 3.05 (q, *J* = 6.5 Hz, 2H), 1.90–1.77 (m, 1H), 1.76–1.65 (m, 1H), 1.62–1.46 (m, 2H), 1.40 (s, 9H). ^{13}C NMR (100 MHz, CD_3CN, Supplementary Figure 16): *δ* 172.09, 157.08, 152.89, 79.24, 58.20, 40.15, 29.43, 28.59, 26.29.

Synthesis of O-(tert-butyl)-L-serine NCA (*t*Bu-L-Ser NCA)

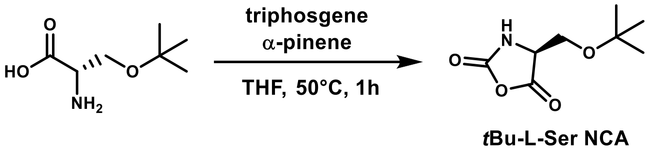

*t*Bu-L-Ser NCA[6] was prepared as a white crystal in 70% yield, from O-(tert-butyl)-L-serine using the similar method described above for Boc-D,L-Lys NCA. ^{1}H NMR (400 MHz, $CDCl_3$): ^{1}H NMR (400 MHz, $CDCl_3$, Supplementary Figure 17): δ 6.36 (br, 1H), 4.42 (dd, *J* = 4.6, 3.3 Hz, 1H), 3.80-3.60 (m, 2H), 1.18 (s, 9H). ^{13}C NMR (100 MHz, $CDCl_3$, Supplementary Figure 18): *δ* 168.32, 153.21, 74.50, 60.67, 59.01, 27.31.

Synthesis of L-Leucine NCA (L-Leu–NCA)

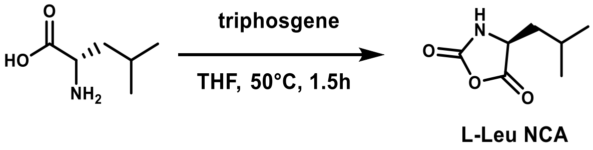

L-Leu–NCA[7, 8] was prepared as a white crystal in 75% yield, from L-leucine using a similar method described above for BLG NCA. ^{1}H NMR (400 MHz, $CDCl_3$, Supplementary Figure 19): δ 7.13 (s, 1H), 4.35 (dd, *J* = 9.0, 4.0 Hz, 1H), 1.90-1.73 (m, 2H), 1.72-1.61 (m, 1H), 0.98 (dd, *J* = 8.8, 6.2 Hz, 6H). ^{13}C NMR (100 MHz, $CDCl_3$, Supplementary Figure 20): *δ* 170.15, 153.31, 56.33, 40.90, 25.13, 22.83, 21.60.

Synthesis of β-tert-butyl-L-aspartate NCA (*t*Bu-L-Asp NCA)

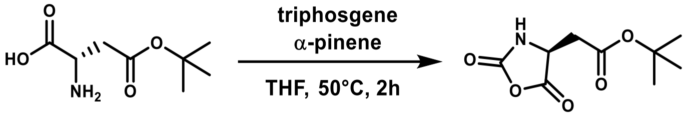

*t*Bu-L-Asp NCA[9] was prepared as a white crystal in 55% yield, from L-aspartic acid β-tert-butyl ester using a similar method described above for Boc-D,L-Lys NCA. ^{1}H NMR (400 MHz, $CDCl_3$, Supplementary Figure 21): ^{1}H NMR (400 MHz, $CDCl_3$) δ 6.51 (s, 1H), 4.54 (dd, J = 8.0, 3.4 Hz, 1H), 2.93 (dd, J = 17.5, 3.6 Hz, 1H), 2.79 (dd, J = 17.5, 8.1 Hz, 1H), 1.46 (s, 9H). ^{13}C NMR (100 MHz, $CDCl_3$, Supplementary Figure 22): δ 168.86, 168.48, 152.06, 83.58, 54.22, 37.10, 28.06.

Preparation of bipyNi(COD)[10, 11]

Supplementary Table 1. Validity test of synthesized bipyNi(COD) for BLG NCA polymerization.

monomer	initiator	M:I	Mn	Đ	DP	Solvent
BLG NCA	bipyNi(COD)	100	28360	1.22	129	DMF
BLG NCA	bipyNi(COD)	500	120500	1.19	550	DMF

The bipyNi(COD) solution (0.1M) in THF was prepared freshly prior to the polymerization reaction; ^{1}H NMR was collected using this solution after diluted with C_6D_6 (Supplementary Figure 23). In a glove box, $Ni(COD)_2$ (27.5 mg, 0.1 mmol) was mixed with 2,2'-bipyridine (15.6 mg, 0.1 mmol) in anhydrous THF (1 mL) at room temperature and the reaction mixture was stirred overnight. To verify validity of bipyNi(COD) solution for NCA polymerization, poly-BLG (target chain length of 100 and 500 mers) were synthesized from a mixture of BLG

NCA (52.6 mg, 0.2 mmol) and 0.02 mL or 0.01 mL of the bipyNi(COD) solution (0.1M). Both polymerizations were conducted in anhydrous DMF at room temperature for 1 day in a glove box. Both polymers were obtained as expected with controlled polymer length (obtained 129 and 550 mer respectively) and a narrow dispersity (1.22 and 1.19 respectively). GPC traces of these polymers are shown in Supplementary Figure 96 and 97 below.

Polymer synthesis and characterization

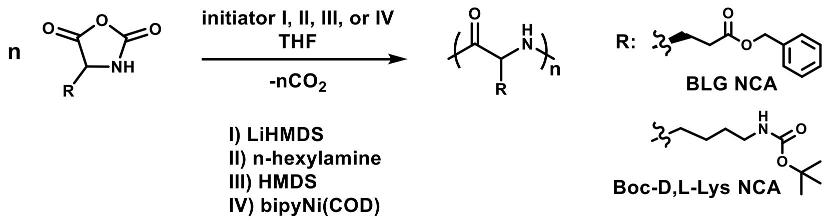

A general synthesis of poly-BLG is described below and all others polymers were synthesized similarly. The polymer length was controlled via the BLG NCA:initiator ratio. In a nitrogen purged glovebox, BLG NCA was weighed out and dissolved in anhydrous THF to final concentration at 0.1 M to 0.5 M, depending on target polypeptide chain length, in a dried reaction vial equipped with a magnetic stir bar. Then a solution of LiHMDS in THF (0.02 M or 0.1 M) was added to the reaction vial quickly. The reaction mixture was stirred at room temperature for 5 min to 2 hours depending on the targeting polypeptide chain length. The poly-BLG_{34}, poly-BLG_{74}, and poly-BLG_{124} were synthesized from a mixture of BLG NCA (26.3 mg, 0.1 mmol) and a 0.1M solution of LiHMDS in THF for 0.2 mL, 0.1 mL and 0.05 mL respectively to reach a final BLG NCA concentration at 0.1 M. The poly-BLG_{357} and poly-BLG_{510} were synthesized from a mixture of BLG NCA (52.6 mg, 0.2 mmol) and a 0.02M solution of LiHMDS in THF for 0.1 mL and 0.02 mL respectively to reach a final BLG NCA

concentration at 0.5 M. NCA monomer conversion was monitored by HPLC using triphenylmethane as the internal standard. After reaction completion, the reaction mixture was removed out of the glovebox followed by quenching with a few drops of HCOOH. After diluting the solution of poly-BLG to 5 mg/mL using DMF (containing 0.01 M LiBr), the obtained solution was analyzed by GPC to measure the absolute molecular weight of poly-BLG. The remaining poly-BLG solution was poured into cold petroleum ether (40 mL) to precipitate out a white fluffy solid. The precipitate was collected by centrifugation and dried under air flow. The collected solid was dissolved in THF (1.5 mL) and then was precipitated out again by adding petroleum ether (40 mL) to the solution. This dissolution-precipitation process was repeated two more times to provide the side-chain protected polymer. Polymerization of Boc-D,L-Lys NCA using LiHMDS as an initiator and characterization of resulting polypeptides were performed by following the protocol similar to that of BLG NCA described above. HMDS and n-hexylamine initiated polymerization of NCA (BLG NCA or Boc-D,L-Lys NCA) and characterization of resulting polypeptides were operated by following the protocol similar to that of LiHMDS initiated polymerization aforementioned. To remove the sidechain O-benzyl protecting group of poly-BLG for specific rotation studies, poly-BLG was dissolved in TFA (1 mL) followed by addition of 33 wt% solution of HBr in AcOH (2 mL). After gentle shaking overnight at room temperature, the reaction mixture was poured into Et_2O (40 mL) to get a white precipitate. The precipitate was collected after centrifugation and dried under N_2 flow. ^1H NMR was collected to confirm full deprotection (Supplementary Figure 24).

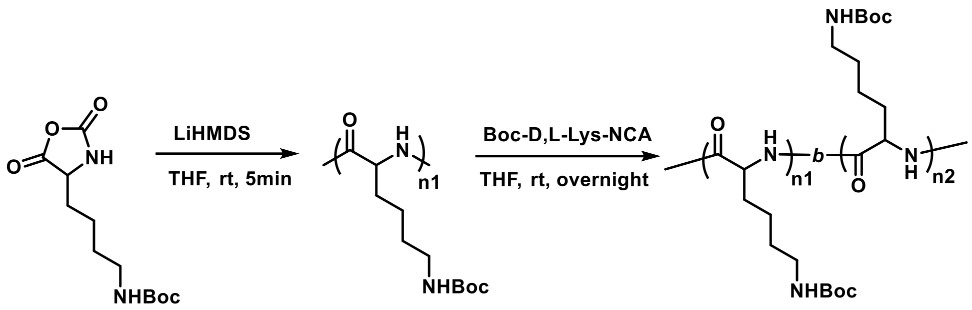

For the synthesis of block co-polymer, Boc-D,L-Lys NCA (27.2 mg, 0.1 mmol) was first dissolved in anhydrous THF (0.7 mL) followed by addition of 0.1 M solution of LiHMDS in THF (0.1 mL). The reaction was performed at room temperature for 5min to get complete conversion of the monomers. Then, half of the reaction mixture was diluted the concentration of to 5 mg/mL for GPC analysis of first block. Boc-D,L-Lys NCA (13.6 mg, 0.05 mmol) in anhydrous THF (0.2 mL) was then added to the reaction mixture and the polymerization of the second block continued overnight to give the block polymer poly(Boc-D,L-Lys)-*b*-poly(Boc-D,L-Lys). These polypeptides were characterized by GPC using DMF as the mobile phase.

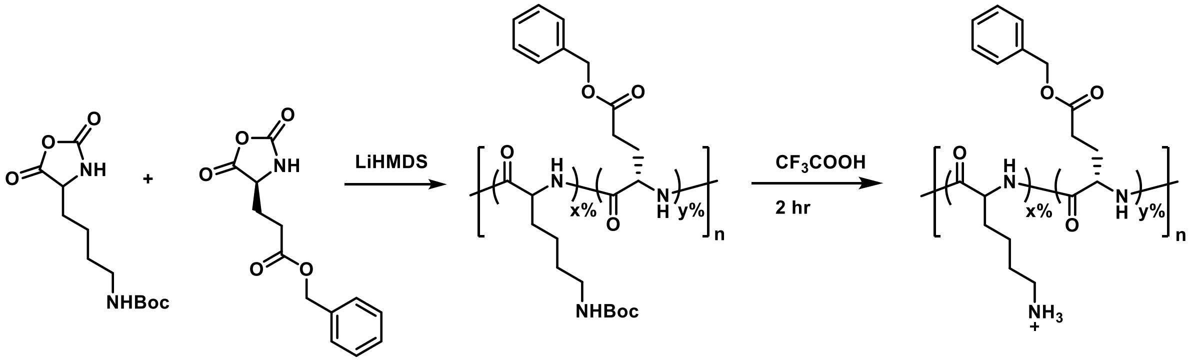

For the synthesis of amphiphilic random co-polymers, a representative synthesis of poly(Boc-D,L-Lys)$_{0.5}$-r-poly(BLG)$_{0.5}$ is described below in a N_2 purged glovebox. Boc-D,L-Lys NCA (40.8 mg, 0.15 mmol) and BLG NCA (39.5 mg, 0.15 mmol) were weighed out and dissolved in anhydrous THF (2 mL) in a dried reaction vial equipped with a magnetic stir bar.

Then a 0.1 M solution of LiHMDS in THF (0.6 mL) was added to the reaction vial. The reaction mixture was stirred at room temperature for 5 min and then was removed out of the glovebox, followed by quenching the reaction with a few drops of HCOOH. The resulting solution was poured into cold petroleum ether (40 mL) to give a white precipitation that was collected by centrifugation and dried under air flow. The collected solid was dissolved in THF (1.5 mL) and precipitated again by adding petroleum ether (40 mL) to the solution. This dissolution-precipitation process was repeated two more times to get poly(Boc-D,L-Lys)$_{0.5}$-r-poly(BLG)$_{0.5}$ in the sidechain protected stage as a white solid. These polypeptides at the sidechain amine protected stage were characterized by GPC using DMF as the mobile phase.

N-Boc protecting groups were removed by treating the side-chain protected polymers with neat trifluoroacetic acid (TFA, 2 mL) at room temperature for 2 hours under gentle shaking. Then TFA was removed under air flow to afford a viscous liquid. The liquid was dissolved in MeOH (0.5 mL) followed by addition of Et_2O (40 mL) to precipitate out the deprotected polymer. The solid was collected after centrifugation and dried under N_2 flow. After three cycles of dissolution/precipitation process, the collected solid was dissolved in milli-Q and subjected to lyophilization. The final polypeptides were obtained as a fluffy white solid and used for further antibacterial activity studies. NMR characterization of deprotected polymers and GPC traces of protected polymers are shown in Supplementary Figure 25-36 below.

NCA stability assay.

In a nitrogen purged glovebox, a mixture of BLG NCA (52.6 mg, 0.2 mmol) and the internal standard triphenylmethane (12.2 mg, 0.05 mmol) was dissolved in anhydrous THF (2

mL) or anhydrous DMF (2 mL) under stir at room temperature without adding any polymerization initiator. Samples were taken from the solution periodically for HPLC analysis to calculate the remaining NCA within the solution. To analyze the stability of Boc-D,L-Lys NCA, a mixture of Boc-D,L-Lys NCA (54.4 mg, 0.2 mmol) and the internal standard triphenylmethane (2.5 mg, 0.01 mmol) was dissolved in anhydrous THF (2 mL) or anhydrous DMF (2 mL) under stir at room temperature without adding any polymerization initiator. The HPLC analysis on Boc-D,L-Lys NCA was similar to the method for BLG NCA mentioned above. Boc-D,L-Lys NCA and BLG NCA were analyzed by the integration of the absorbance peak at 210 nm and 254 nm respectively.

Circular Dichroism (CD) spectroscopy analysis

Poly-BLG at variable chain length were dissolved in hexafluoroisopropanol (HFIP) to a concentration of 0.25 mg/mL and the polymer solutions were measured repeatedly at 20 °C in a quartz cuvette with optical path length of 0.1 cm. Mean residue ellipticity [θ] was calculated using the equation:

$$[\theta] = \frac{\theta \cdot M_{\text{repeating unit}}}{10 \cdot C \cdot L}\ [\text{deg}\cdot\text{cm}^2\cdot\text{dmol}^{-1}] \qquad \text{(Supplementary Equation 1)}$$

with $M_{\text{repeating unit}}$ = 219.1 g/mol, C = 0.25 g/L, L = 0.1 cm. CD spectra of poly-BLG_{34}, poly-BLG_{74}, poly-BLG_{124} show a positive band at 195 nm and two negative bands at 208 nm and 220 nm, which are characteristic bands of α-helix.

Polarimetry measurement

Chirality of the deprotected polymers was measured at λ=589 nm using the Autopol® V automatic polarimeter. Deprotected polymers were prepared in milli-Q at final concentration of 10 mg/mL and measured at pH=8 and at 25 °C. Specific rotation ($[\alpha]\frac{T}{\lambda}$) was calculated using the following equation:

$$[\alpha]\frac{T}{\lambda} = \frac{100\alpha}{l \cdot c} \qquad \text{(Supplementary Equation 2)}$$

Where α is the angle of rotation, l is the length of the polarimeter tube (dm), c is polymer concentration (g/100 mL).

Terminal functionalization for polypeptides prepared from LiHMDS-initiated polymerization

After completion of the polymerization reaction, the poly-Boc-D,L-Lys at sidechain amine protected (NHBoc) stage was characterized by GPC for absolute molecular weight measurement. GPC result indicated the poly-Boc-D,L-Lys have an average polymer length of 20 subunits (DP=20). 4-tert-butylbenzylamine was added as a nucleophilic reagent to the reaction mixture in THF and the reaction was allowed to stir overnight. The reaction mixture was then poured into petroleum ether to precipitate out the resulting polymer as a white solid and the solid was collected after centrifugation. This dissolution-precipitation process was repeated two more times and the white solid was dried under vacuum to get the C-terminal functionalized polypeptide. The polymer (25 mg, 5.5 μmol)) was dissolved in THF (1 mL), then N-succinimidyl 3-maleimidopropionate (30 mg, 0.11 mmol) was added to the solution. After overnight reaction, the reaction mixture was poured into petroleum ether (40 mL) to precipitate out a white solid.

The white solid was collected after centrifugation and then was washed with acetonitrile (1 mL × 3) to make sure all N-succinimidyl 3-maleimidopropionate was removed from the polymer. The precipitated polypeptide was dried under vacuum to obtain N-terminal functionalization for polypeptides that have dual functionalization at the C- and N-termini of the polypeptide (Supplementary Figure 37). The N-terminus functionalization for poly-BLG (Supplementary Figure 38) was conducted similarly to that of poly-Boc-D,L-Lys.

Analysis of subunit distribution within amphiphilic co-polymers

To evaluate the subunit distribution within an amphiphilic co-polymer from LiHMDS initiated NCA polymerization, consumption of NCA was monitored by reverse phase HPLC. A mixture of 1:1 Boc-D,L-Lys : BLG in THF was used for the analysis and the polymerization was initiated with LiHMDS. A solution of BLG NCA (26.3 mg, 0.1 mmol) and Boc-D,L-Lys NCA (27.2 mg, 0.1 mmol) in THF (7.6 mL) was mixed with a 0.1 M solution of LiHMDS (0.4 mL) at room temperature with stirring. Triphenylmethane (TPM) was used as the internal standard. Samples of the reaction mixture at different intervals were taken for HPLC analysis on remaining NCA in the reaction mixture. Boc-D,L-Lys NCA and BLG NCA were both analyzed by integration of the absorbance peak at 210 nm. Boc-D,L-Lys subunit composition within polymer chains (y axis) was calculated from the equation below.

$$y = \left(\frac{1-\frac{\left(A_{\text{Boc-D,L-Lys NCA}}/A_{\text{TPM}}\right)_{t}}{\left(A_{\text{Boc-D,L-Lys NCA}}/A_{\text{TPM}}\right)_{t=0}}}{\left(1-\frac{\left(A_{\text{Boc-D,L-Lys NCA}}/A_{\text{TPM}}\right)_{t}}{\left(A_{\text{Boc-D,L-Lys NCA}}/A_{\text{TPM}}\right)_{t=0}}\right)+\left(1-\frac{\left(A_{\text{BLG NCA}}/A_{\text{TPM}}\right)_{t}}{\left(A_{\text{BLG NCA}}/A_{\text{TPM}}\right)_{t=0}}\right)}\right)\times 100\% \quad \text{(Supplementary Equation 3)}$$

Total NCA monomer conversion (x axis) was calculated from the equation below.

$$x = \left(1 - \frac{\left((A_{\mathrm{Boc-D,L-Lys\,NCA}} + A_{\mathrm{BLG\,NCA}})/A_{\mathrm{TPM}}\right)_t}{\left((A_{\mathrm{Boc-D,L-Lys\,NCA}} + A_{\mathrm{BLG\,NCA}})/A_{\mathrm{TPM}}\right)_{t=0}}\right) \times 100\%. \qquad \text{(Supplementary Equation 4)}$$

Antibacterial activity studies

The fresh cultured bacteria in LB (Luria-Bertani) medium at 37 °C was diluted in MH (Mueller-Hinton) medium to a cell density of 2×10^5 CFU/mL as the working suspension. Two-fold serial dilution of polypeptides was performed in a 96-well plate using MH medium to provide each well of 50 µL solution with polypeptide concentration ranging from 3.13 to 400 µg/mL. An aliquot of 50 µL above bacteria cell working suspension was added to each well, followed by gentle shaking of the plate for 10 second. The plate was incubated at 37 °C for 9 hours, and then the optical density (OD) of each well was measured at 600 nm using a Molecular Devices SpectraMax M2 precision microplate reader. Wells containing only MH medium was used as the blank and wells containing cells in MH without polymer was used as the positive control on the same plate. Measurements were performed in duplicates, and the experiments were repeated at least twice on different days. The percentage of bacteria cell growth in each well was calculated from the equation below and plotted against polypeptide concentration to give the dose-response curves of antibacterial activity for these polypeptides.

$$\text{cell growth} = \left(\frac{\mathbf{A}_{600}^{\text{polymer}} - \mathbf{A}_{600}^{\text{blank}}}{\mathbf{A}_{600}^{\text{control}} - \mathbf{A}_{600}^{\text{blank}}}\right) \times 100\% \qquad \text{(Supplementary Equation 5)}$$

The MIC value is defined as the minimum concentration of a polypeptide to inhibit bacterial growth.

Supplementary Discussion

NCA stability in THF vs. DMF.

Most of the NCA polymerizations in literatures continued for 2-3 days using DMF or THF as the solvent. However, many NCAs are quite unstable and the decomposition can bring side reactions.[12, 13] We analyzed the stability of widely used γ-benzyl-L-glutamate NCA (BLG NCA) and Nε-tert-butyloxycarbonyl-D,L-lysine NCA (Boc-D,L-Lys NCA) in dry DMF and THF at room temperature without addition of any polymerization initiator. We found that BLG NCA is stable in dry THF for at least 22 hours, but its concentration reduced quickly in DMF from the very beginning (Supplementary Figure 1a). Boc-D,L-Lys NCA is stable in dry THF for at least 96 hours, but observable loss of Boc-D,L-Lys NCA starts after 50 hours in dry DMF (Supplementary Figure 1b). In general, both BLG NCA and Boc-D,L-Lys NCA are more stable in dry THF than in dry DMF. To Figure out the reason for this observation we checked the content of chloride ions within BLG NCA and Boc-D,L-Lys NCA because chloride ions are able to initiate NCA polymerization in DMF.[14] High Resolution Inductively Coupled Plasma Mass Spectrometry (ICP-MS) analysis indicated a very low level of chloride ions at 1.5 ppm and 5.5 ppm respectively for BLG NCA and Boc-D,L-Lys NCA. This result implies that impurity of chloride ions in NCA is likely not a major reason for observed stability issue of NCA in DMF. Another possible reason for observed difference in NCA stability is that dry DMF generally may have more water content than does dry THF. Therefore, we carefully measured the water content of anhydrous DMF and THF using a Karl Fisher titrator. The actual water content was found to be 80 ppm and 46 ppm respectively for our DMF and THF. This result implies moisture content within solvents may have effect on NCA stability in different DMF and THF. The water content of anhydrous solvent can vary widely by manufacturer, lot number, and in laboratory handling.

Solvent still is also subject to the technique of the chemist. DMF is particularly challenging to dry and it may be contaminated with other impurities like dimethylamine and cause side reactions. These observations and analysis encouraged us to develop a fast polymerization of NCAs and choose THF as the reaction solvent to avoid possible NCA decomposition and side reactions associated with DMF.

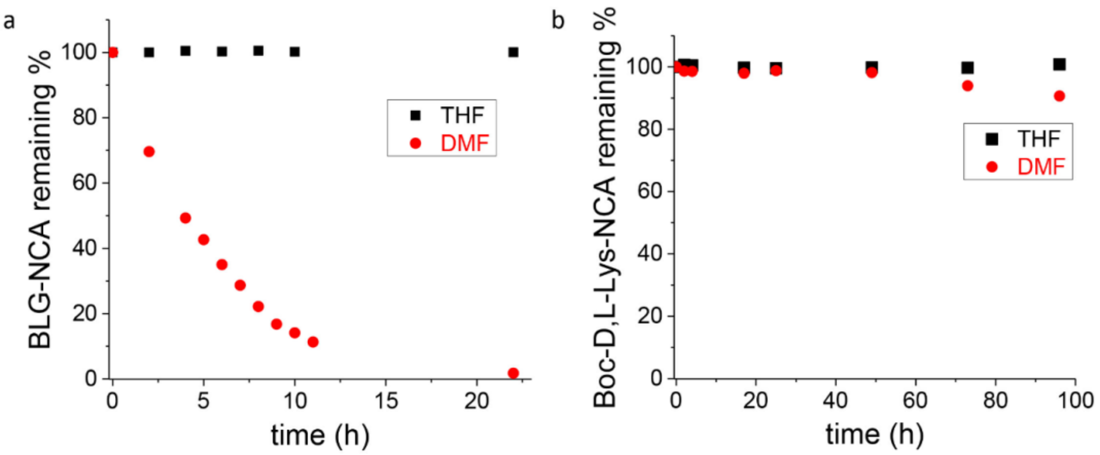

Supplementary Figure 1: NCA stability in anhydrous THF or DMF. a) stability of BLG NCA in anhydrous THF or DMF without any polymerization initiator; b) stability of Boc-D,L-Lys NCA in anhydrous THF or DMF without any polymerization initiator.

Tests on different commercial LiHMDS.

To examine the validity of LiHMDS from different commercial sources, we did the demonstration to synthesize poly-BLG_{350} via BLG NCA polymerization using different LiHMDS as the initiator. These chemicals include LiHMDS derived from two different synthetic methods (synthesized from lithium metal or n-BuLi), LiHMDS from three different commercial suppliers (Sigma-Aldrich, HWRK Chem and Adamas Reagent), and LiHMDS with four different lot numbers from Sigma-Aldrich. LiHMDS from different commercial resources all gave desired polypeptides with similar results regarding reaction time, reaction yield, number average molecular weight (Mn), dispersity index (Đ) and degree of polymerization (DP).

a

entry	initiator	commercial source	lot number	preparation method	GPC characterization		
					Mn	Đ	DP
1	LiHMDS	Sigma-Aldrich	SHBH2360V	N/A	76660	1.20	350
2	LiHMDS	Sigma-Aldrich	SHBH9931	N/A	77520	1.20	354
3	LiHMDS	Sigma-Aldrich	SHBH9029	N/A	76950	1.20	351
4	LiHMDS	Sigma-Aldrich	SHBH8213	N/A	76860	1.20	351
5	LiHMDS	HWRK Chem	SPBGH38A	Li metal process	75990	1.21	347
6	LiHMDS	Adamas reagent	P1353836	n-BuLi and $HN(TMS)_2$	77750	1.20	355

b

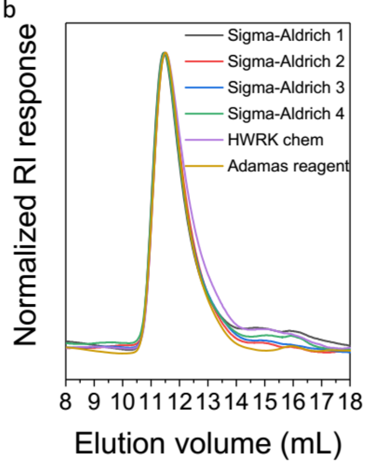

Supplementary Figure 2: NCA polymerization initiated with different commercial LiHMDS. a) Characterization of poly-BLG from NCA polymerization initiated by different commercial LiHMDS. N/A = not available (trade secret); b) GPC traces of resulting poly-BLG. All full scale GPC traces were included in the Supplemental Figure 90-95.

HPLC analysis on NCA stability and polymerization progress

Reverse phase HPLC analysis on NCA stability and reaction progress used the combination of water (eluent A) and acetonitrile (eluent B) as the mobile phase. For isocratic elution, 90% B was used as the mobile phase to run for 6 min at a flow rate of 1 mL/min. For gradient elution, 60-100% B was used as the mobile to run for 16 min at a flow rate of 1 mL/min. BLG NCA were analyzed by integration of the absorbance peak at 254 nm. Boc-D,L-Lys NCA and Boc-L-Lys NCA were analyzed by integration of the absorbance peak at 210 nm. To examine if the water in the HPLC eluent can have significant effect on the result of analysis during the short time of HPLC elution, we did stability studies on NCA in 90% and 60% acetonitrile. Since BLG NCA is much less stable than the Boc-D,L-Lys NCA, we examined if the HPLC condition is compatible with BLG NCA. According to HPLC result, the retention time for the BLG NCA is less than 3 min using 90% acetonitrile as the isocratic mobile phase, and the retention time for the BLG

NCA is 4 min using 60-100% acetonitrile as the gradient mobile phase. The BLG NCA was dissolved in either 90% or 60% acetonitrile and kept for a series of chosen time. Then the NCA solution was injected into a HPLC using 100% acetonitrile as the mobile phase to calculate the remaining BLG NCA using an internal standard and a calibration curve. Based on our studies (Supplementary Figure 3), 99.8% and 98.9% of BLG NCA remains after the compound was kept for 4 mins in 90% and 60% acetonitrile respectively. The retention time for the BLG NCA is less than 3 min using 90% acetonitrile as the isocratic mobile phase, and the retention time for the BLG NCA is 4 min using 60-100% acetonitrile as the gradient mobile phase. That means the loss of NCA under our HPLC analysis condition is trivial and the data and result won't be skewed by possible NCA hydrolysis.

The calibration curve for calculating remaining NCA within the reaction was prepared by HPLC analysis of a series of mixture solutions composed of BLG NCA:triphenylmethane (TPM) (x:y), with x:y =0.625, 1.25, 2.5, 5, 10, 20, 40, and 80 (w/w). NCA were analyzed by integration of the absorbance peak at 254 nm and the collected data were plotted as A_{BLG}/A_{TPM} (Y axis) against M_{BLG}/M_{TPM} (X axis), where A_{BLG}/A_{TPM} is the integrated areas ratios of HPLC peaks for BLG NCA and TPM, and the M_{BLG}/M_{TPM} is the weight ratios of BLG NCA and TPM (Supplementary Figure 4).

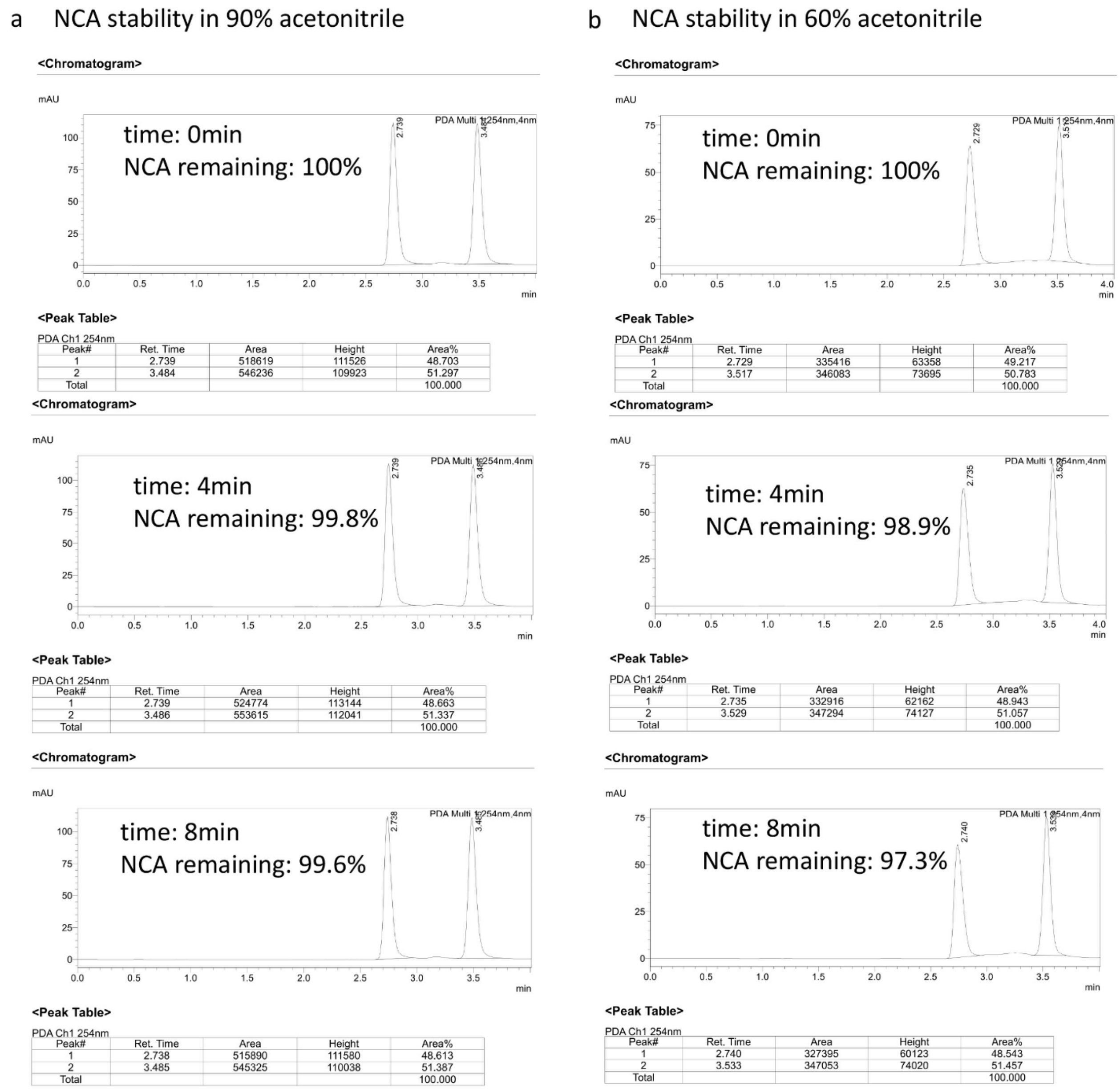

Supplementary Figure 3: Analysis on BLG NCA stability in 90% acetonitrile and 60% acetonitrile for different time. BLG NCA solution kept in either 90% acetonitrile or 60% acetonitrile for variable time was injected into a reverse phase HPLC and was analyzed using 100% acetonitrile as the mobile phase.

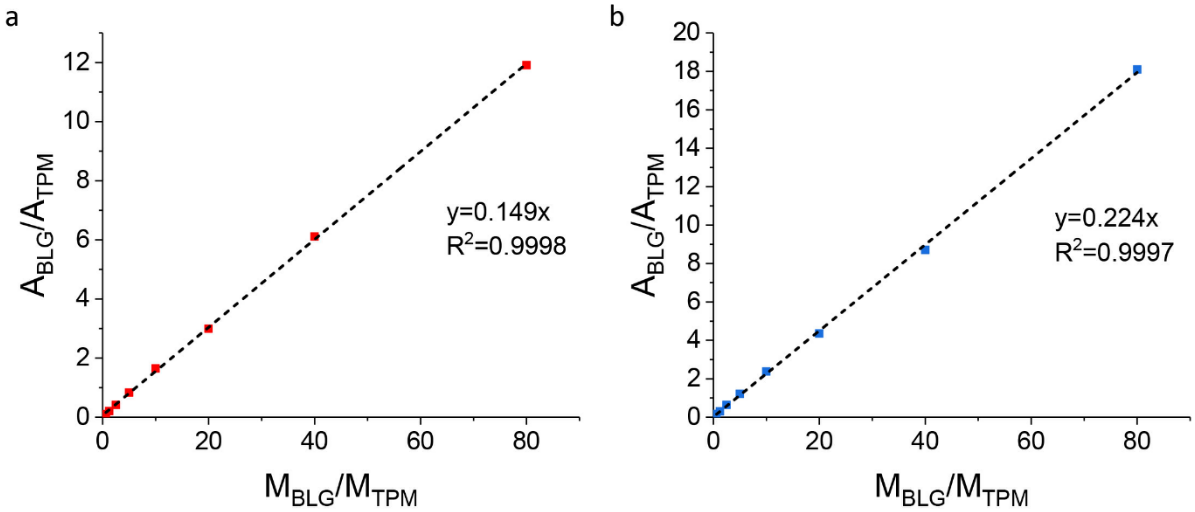

Supplementary Figure 4: Calibration curve of the BLG/triphenylmethane (TPM) where A_{BLG}/A_{TPM} is the integrated areas ratios of HPLC peaks for BLG NCA and triphenylmethane; the M_{BLG}/M_{TPM} is the weight ratios of BLG NCA and triphenylmethane. a) Calibration using isocratic mobile phase (90% acetonitrile) as the mobile phase; b) calibration using gradient mobile phase (60%-100% acetonitrile).

NCA polymerization kinetic studies

A solution of BLG NCA (52.6 mg, 0.2 mmol) in anhydrous THF (1.9 mL) was mixed with a 0.02 M solution of LiHMDS (100 μL) or a 0.02 M solution of n-hexylamine (100 μL) respectively at room temperature under stirring. The real-time monitoring of remaining NCA was performed using FT-IR spectroscopy by measuring the relative ratio of peak area between the NCA carbonyl at 1785 cm^{-1} and the side chain benzyl-group at 1685 cm^{-1} to calculate $\ln\frac{[M]_0}{[M]}$ from the equation below.

$$\ln\frac{[M]_0}{[M]} = \ln\frac{(A_{1785cm^{-1}}/A_{1685cm^{-1}})_{t=0}}{(A_{1785cm^{-1}}/A_{1685cm^{-1}})_{t}}. \qquad \text{Supplementary Equation 6}$$

HPLC was also used to measure remaining NCA within the reaction mixture by calculating the relative ratio of peak area between the NCA and the internal standard. The value of $\ln\frac{[M]_0}{[M]}$ was calculated from the equation below.

$$\ln\frac{[M]_0}{[M]} = \ln\frac{(A_{NCA}/A_{TPM})_{t=0}}{(A_{NCA}/A_{TPM})_t} \qquad \text{Supplementary Equation 7}$$

Then the polymerization rate was calculated from plotting the natural logarithm of NCA concentration vs. reaction time using below equation.

$$-\frac{d[M]}{dt} = k_p[I][M] \qquad \text{Supplementary Equation 8}$$

$$\ln\frac{[M]_0}{[M]} = k_p[I]t \qquad \text{Supplementary Equation 9}$$

Whereas, [I] is the concentration of initiator that equals to total concentration of propagating species and k_p is the rate constant for chain propagation. [M] is residual concentration of the monomer and $[M]_o$ is initial concentration of the monomer. $k_p[I]$ represents the rate of polymerization and was calculated from a linear fitting of the plot that depicts natural logarithm of NCA concentration vs. reaction time.

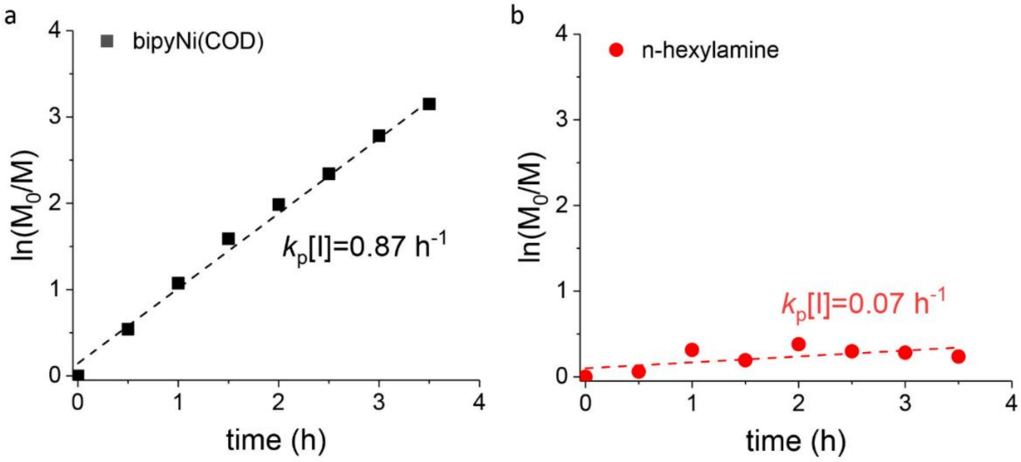

Supplementary Figure 5: Reaction rate of bipyNi(COD) and n-hexylamine initiated BLG NCA polymerization in THF with NCA:initiator ratio of 100:1 and initial NCA concentration at 0.2 M. a) The reaction rate of bipyNi(COD) initiated BLG NCA polymerization monitoring by HPLC; b) the reaction rate of n-hexylamine initiated BLG NCA polymerization monitoring by FT-IR.

Polypeptide backbone racemization evaluation

To mimic polypeptides prepared with variable degree of backbone racemization, we synthesized a series of polypeptides from a mixture of γ-benzyl-L-glutamate NCA (BLG NCA) and γ-benzyl-D-glutamate NCA (BDG NCA) by increasing the content of BDG NCA incrementally from 0% to 50% within the NCA mixture. The obtained polypeptides will have incrementally increased ratio of D-amino acid subunit to mimic the polypeptides with variable degree of backbone racemization. We found a linear relationship between the specific rotation (Y axis) and the percentage of BLG NCA (the X axis) within deprotected polypeptides at pH 8.0 (Supplementary Figure 6). According to this result, the specific rotation of deprotected polypeptide is very sensitive to backbone racemization. Therefore, possible backbone racemization of LiHMDS initiated polymerization of BLG NCA can be evaluated from the measured specific rotation of deprotected polypeptides. Three batches of polypeptides were synthesized from LiHMDS initiated polymerization of BLG NCA and the resulting polypeptides were deprotected to measure their specific rotation at pH 8.0. These three deprotected polypeptides have specific rotation in the range of – 78 to – 83 deg·dm-1·g-1·mL that falls into the expected specific rotation range of poly-BLG.

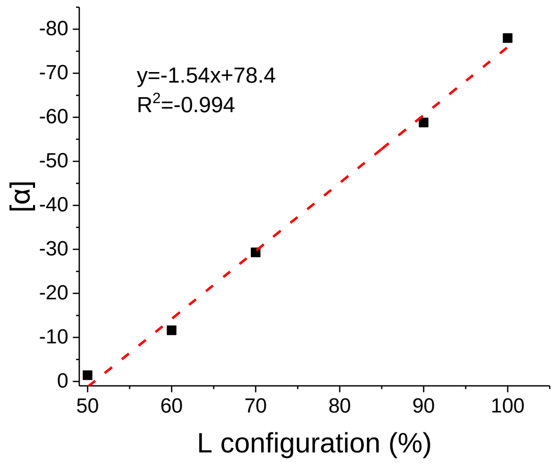

Supplementary Figure 6: Plot of specific rotation against the content of BLG subunit (L configuration) within the polypeptides. Specific rotation of the deprotected polypeptide in milli-Q water was measured at pH 8.0 and room temperature.

Polymerization mechanism studies

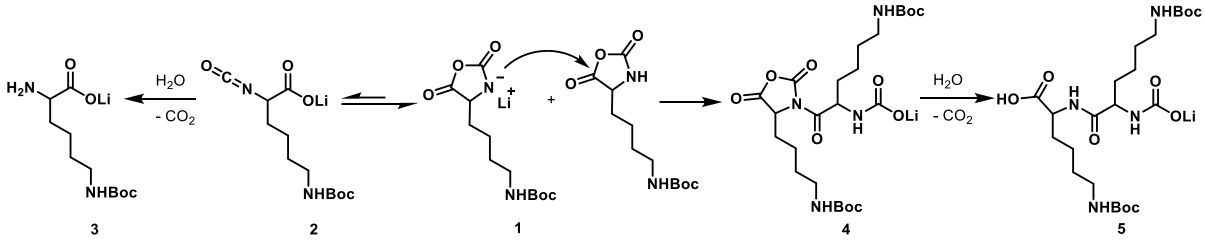

For FT-IR analysis on equal molar mixture of Boc-D,L-Lys NCA and LiHMDS, a Boc-D,L-Lys NCA (27.2 mg, 0.1 mmol) solution in anhydrous THF (500 μL) was added dropwise to a LiHMDS (16.7 mg, 0.1 mmol) solution in anhydrous THF (500 μL) under stir. The FT-IR spectrum was collected using a KBr salt plate on a Nicolet 6700 FT-IR spectrophotometer. The disappeared anhydride peaks of Boc-D,L-Lys NCA at 1875 cm^{-1} and 1785 cm^{-1} indicated the ring opening of NCA. The peak at 1685 cm^{-1} belongs to the side chain carbonyl and exists all the time. The peak at 2231 cm^{-1} indicates the formation of isocyanate and the peak at 1598 cm^{-1} is attributed to the carbonyl absorption of (C=O)OLi.

For ESI-MS analysis on equal molar mixture of Boc-D,L-Lys NCA and LiHMDS, a Boc-D,L-Lys NCA (27.2 mg, 0.1 mmol) solution in anhydrous THF (500 μL) was added dropwise to a LiHMDS (16.7 mg, 0.1 mmol) solution in anhydrous THF (500 μL) under stir. An aliquot of 10 μL reaction solution was added to anhydrous acetonitrile (1 mL) and the diluted solution was kept with care to avoid exposure to moisture. HRESI-MS was collected on a Waters XEVO G2 TOF mass spectrometer. Fragments at m/z 279.1532 and 253.1729 indicates the formation of isocyanate intermediate **2** from NH-deprotonated Boc-D,L-Lys NCA **1** and the following conversion of the intermediate **2** to free amine fragment **3** respectively. In addition, the observation of fragments at m/z 551.2904 and 525.3106 indicates the formation of dimer **4** and following conversion of **4** to fragment **5** respectively.

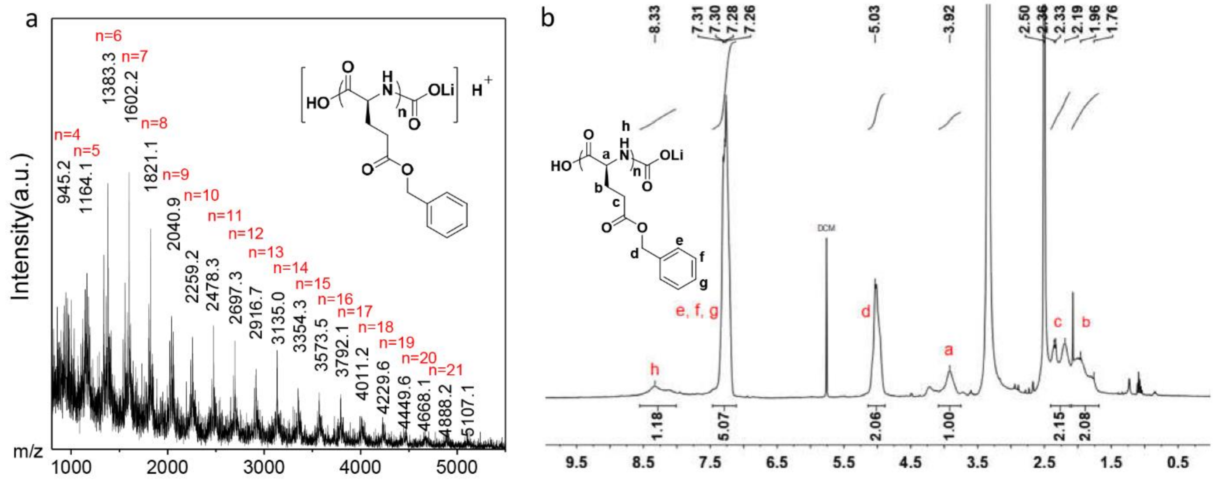

Supplementary Figure 7: Characterization of poly-BLG synthesized from LiHMDS initiated BLG NCA polymerization. a) MALDI-TOF mass spectrum of poly-BLG_{35} using 2,5-Dihydroxybenzoic acid as the matrix; b) ^{1}H NMR of poly-BLG solution in DMSO-*d*6.

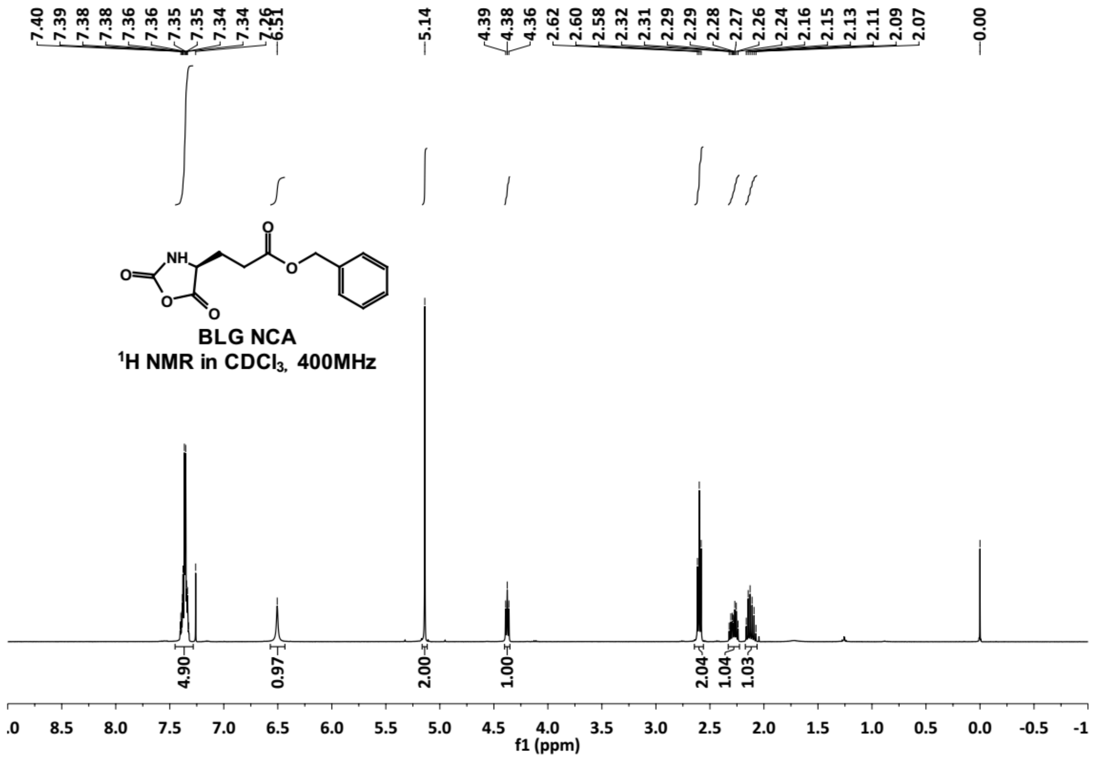

Supplementary Figure 8: ^{1}H NMR for BLG NCA in $CDCl_3$, 400MHz .

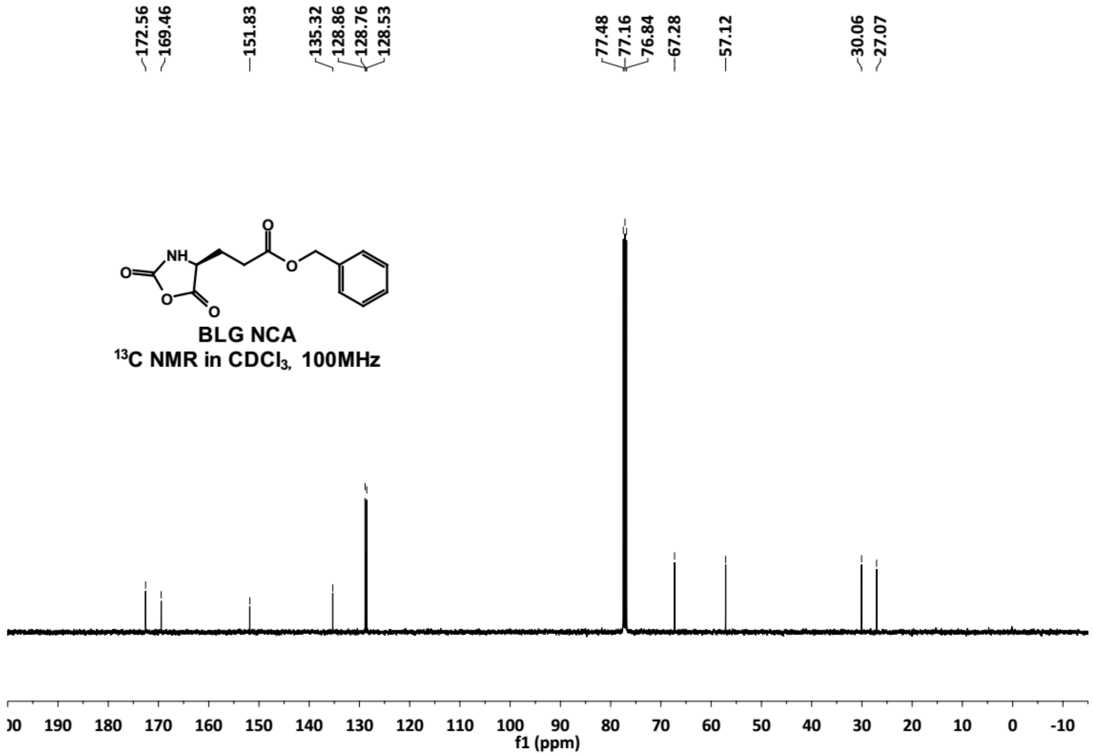

Supplementary Figure 9: ^{13}C NMR for BLG NCA in $CDCl_3$, 100MHz.

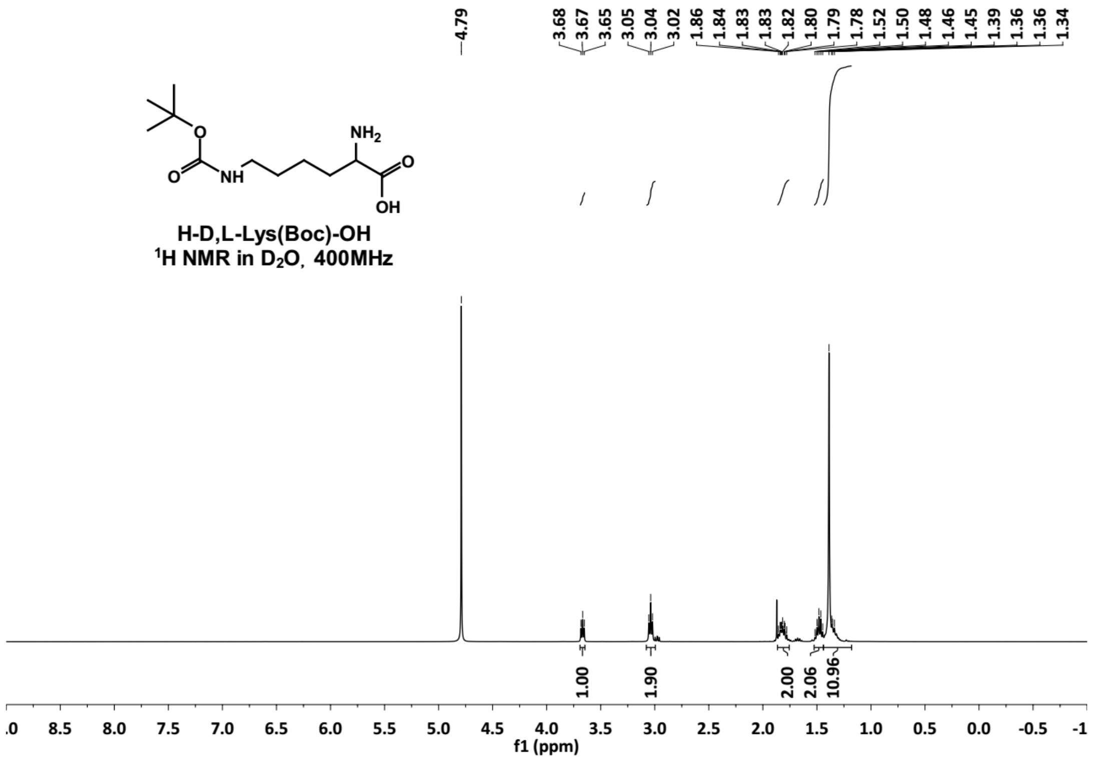

Supplementary Figure 10: ^{1}H NMR for H-D,L-Lys(Boc)-OH in D_2O, 400MHz.

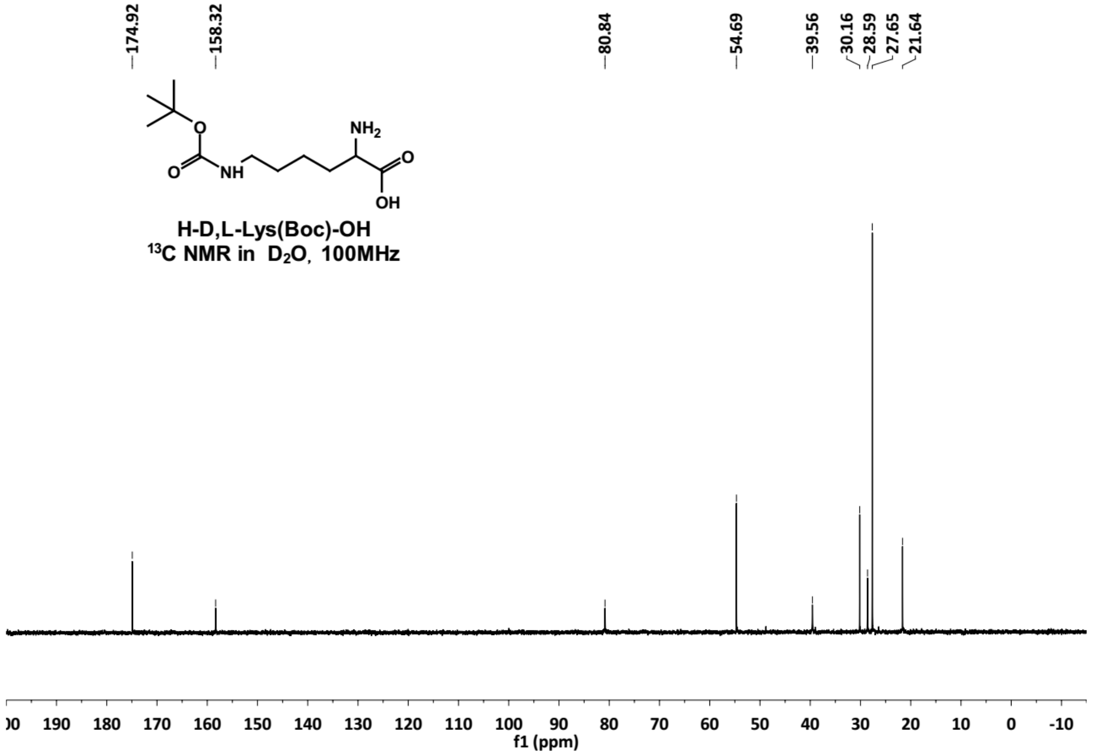

Supplementary Figure 11: ^{13}C NMR for H-D,L-Lys(Boc)-OH in D_2O, 100MHz.

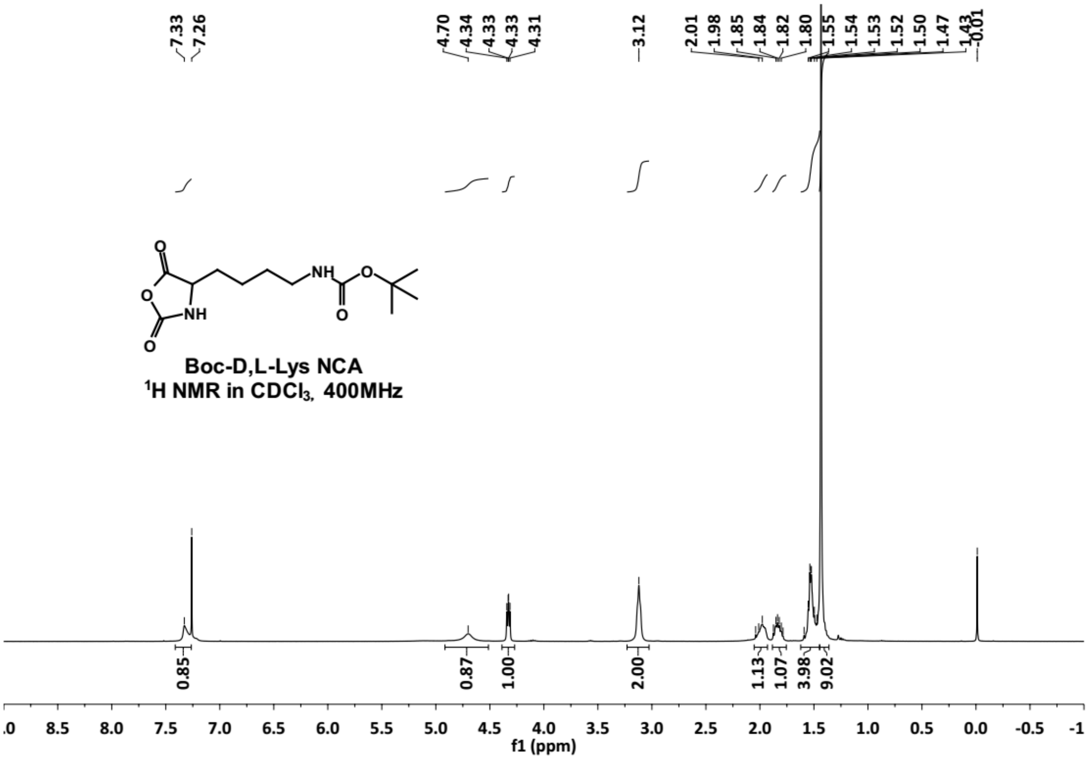

Supplementary Figure 12: ^{1}H NMR for Boc-D,L-Lys NCA in $CDCl_3$, 400MHz.

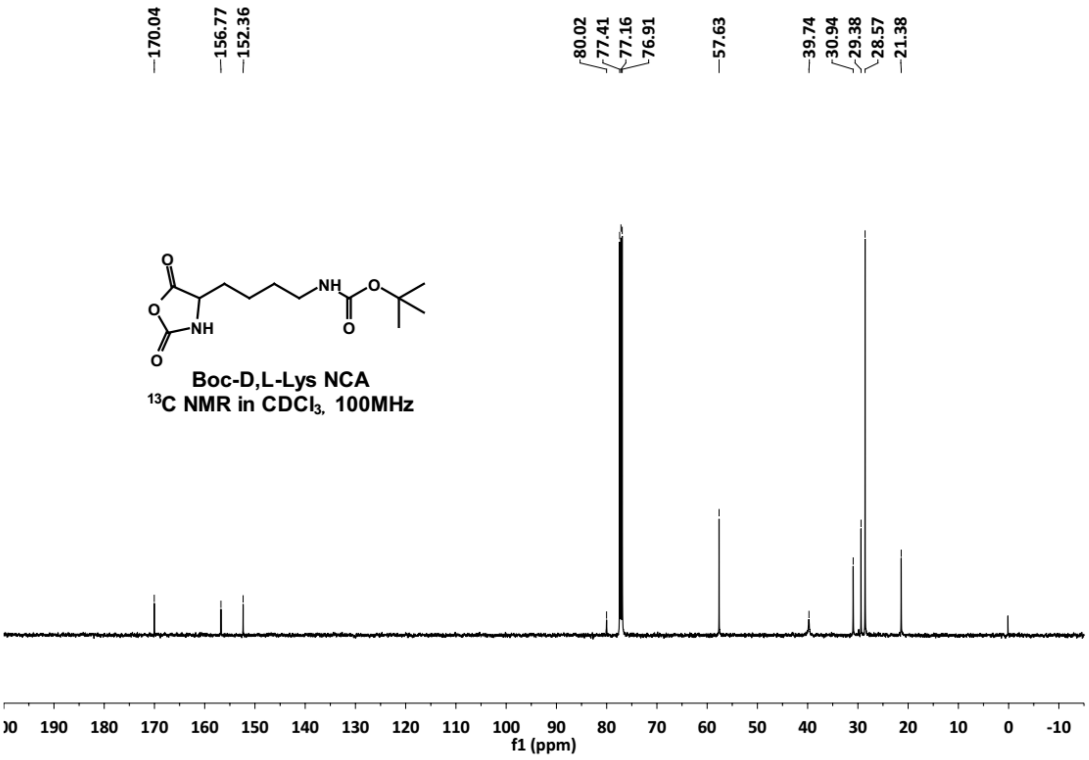

Supplementary Figure 13: ^{13}C NMR for Boc-D,L-Lys NCA in $CDCl_3$, 100MHz.

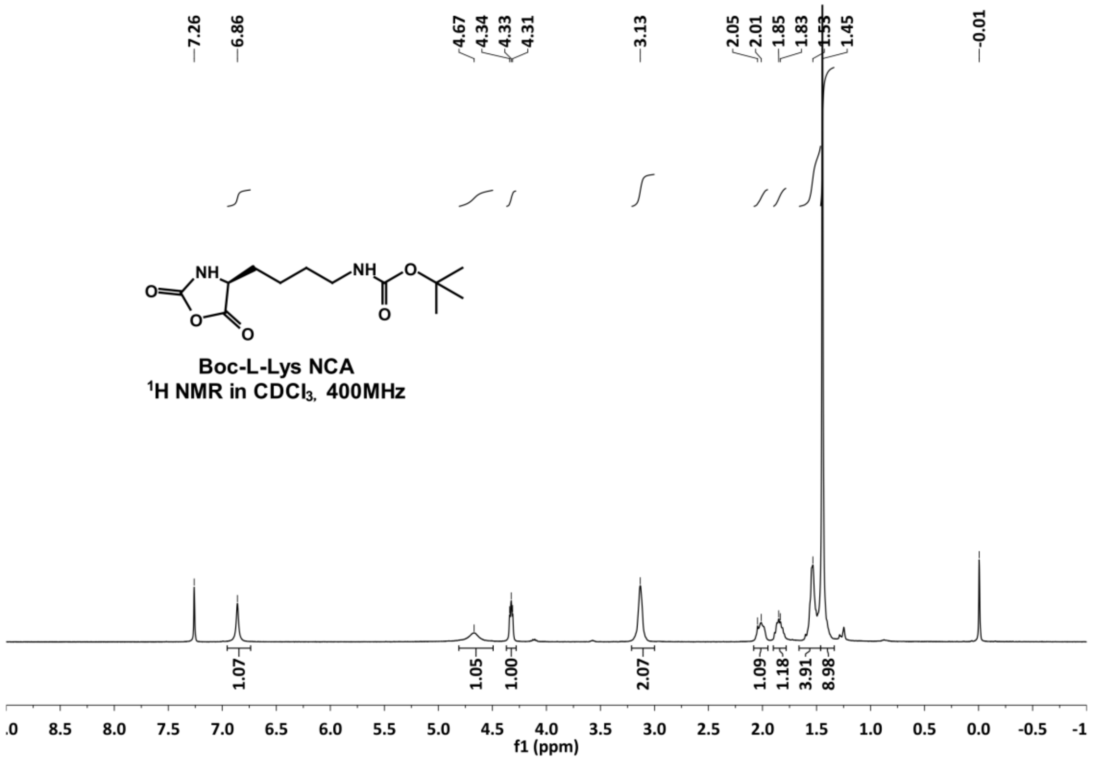

Supplementary Figure 14: ^{1}H NMR for Boc-L-Lys NCA in $CDCl_3$, 400MHz.

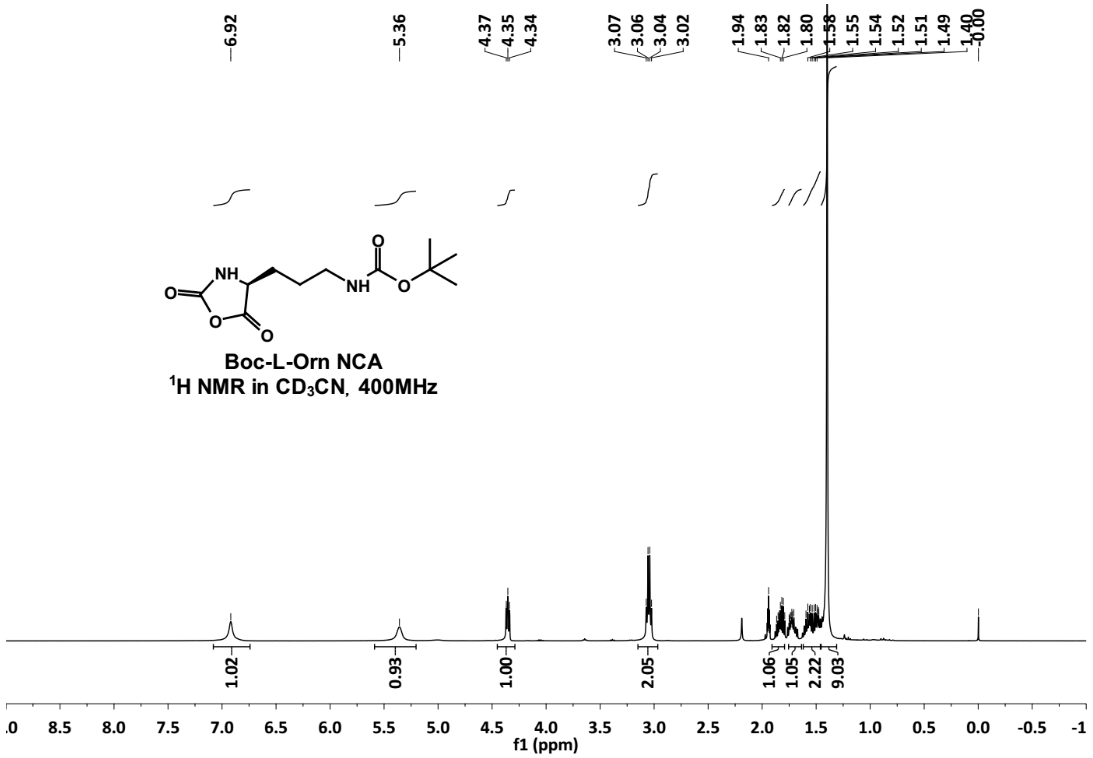

Supplementary Figure 15: ^{1}H NMR for Boc-L-Orn NCA in CD_3CN, 400MHz.

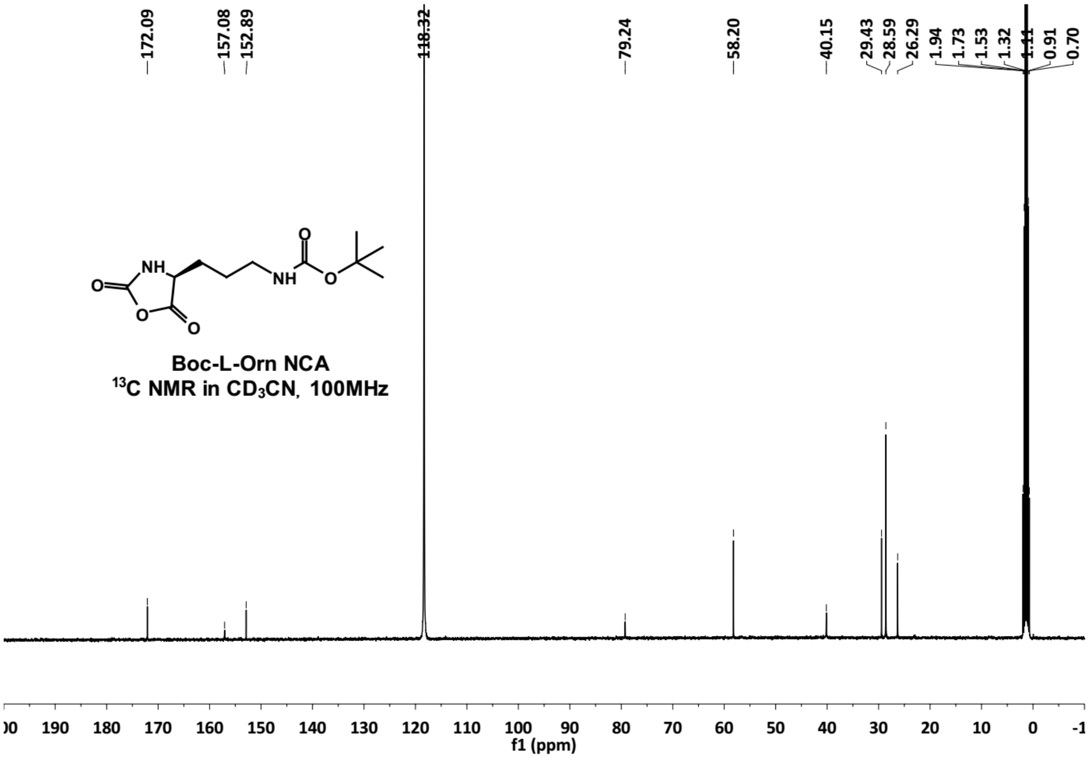

Supplementary Figure 16: ^{13}C NMR for Boc-L-Orn NCA in CD_3CN, 100MHz

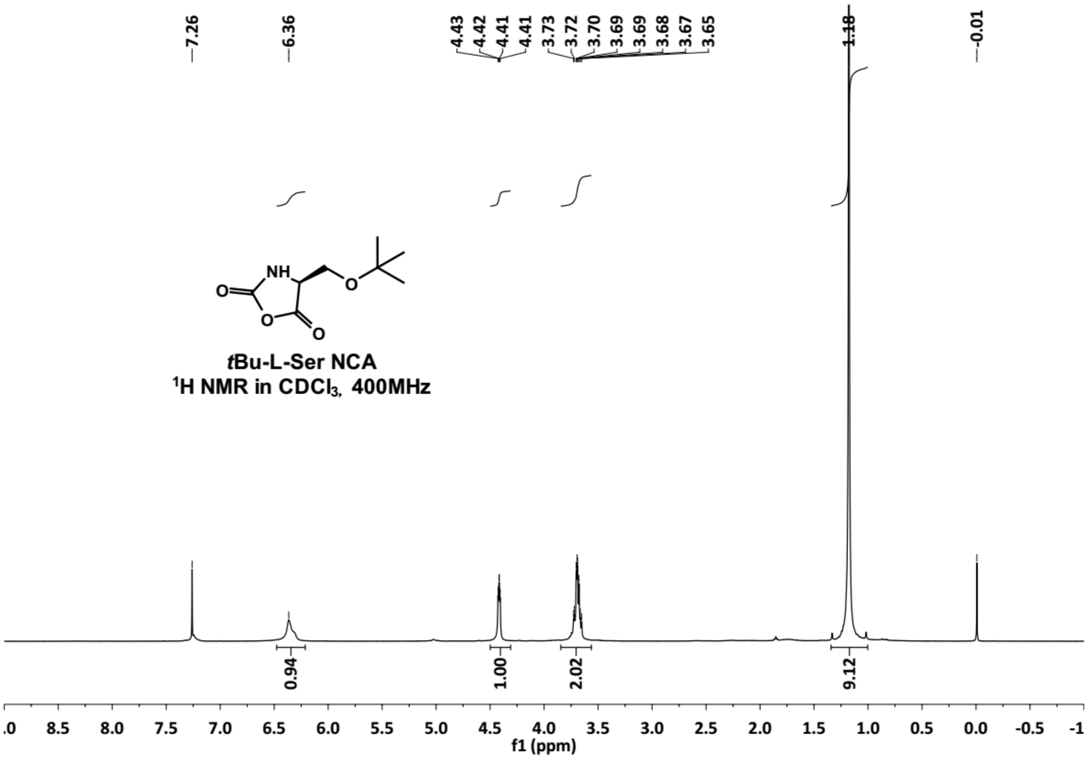

Supplementary Figure 17: ^{1}H NMR for *t*Bu-L-Ser NCA in $CDCl_3$, 400MHz.

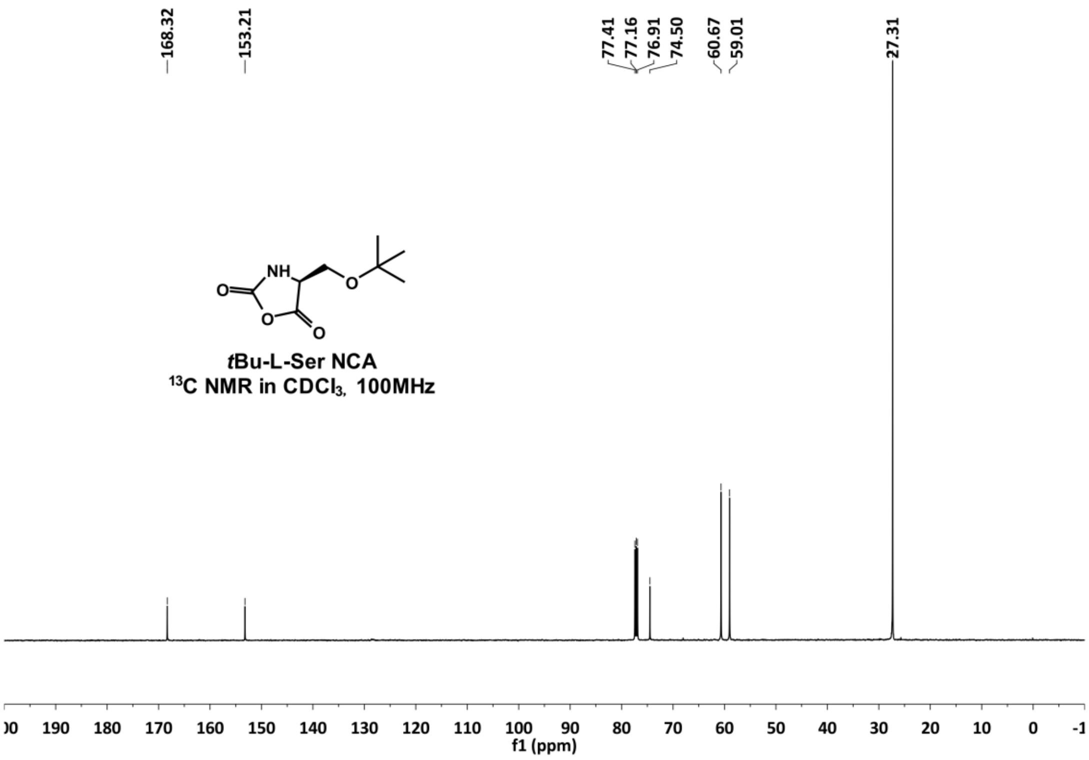

Supplementary Figure 18: ^{13}C NMR for *t*Bu-L-Ser NCA in $CDCl_3$, 100MHz.

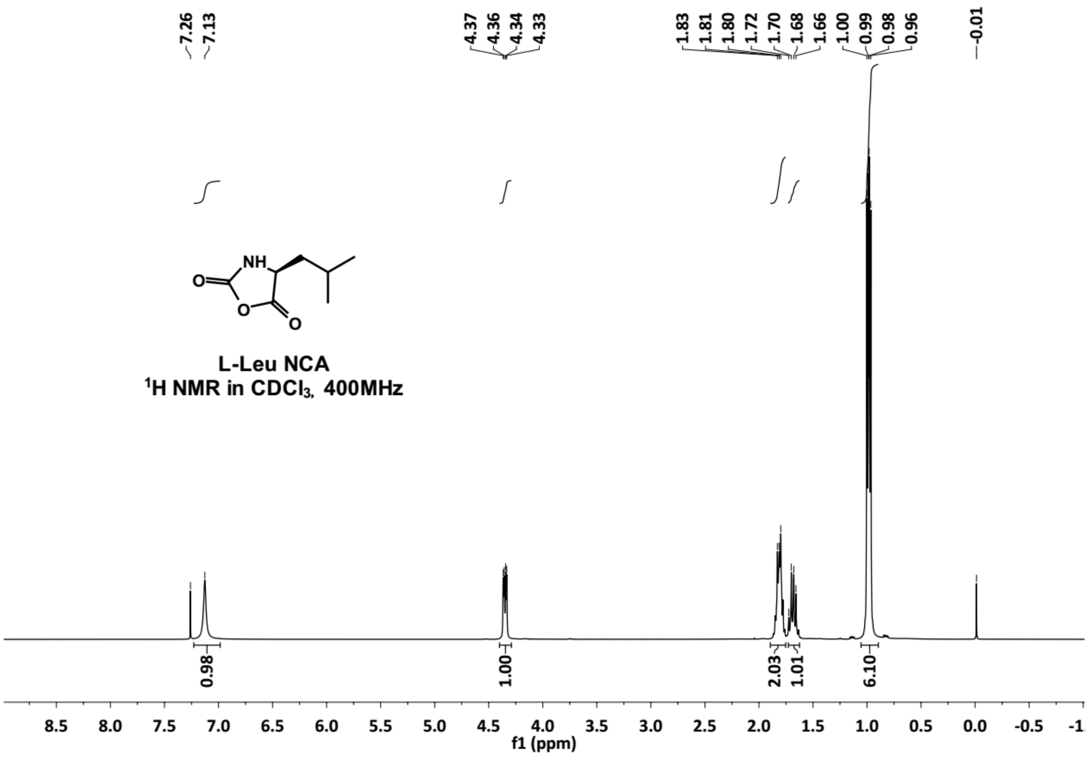

Supplementary Figure 19: ^{1}H NMR for L-Leu NCA in $CDCl_3$, 400MHz.

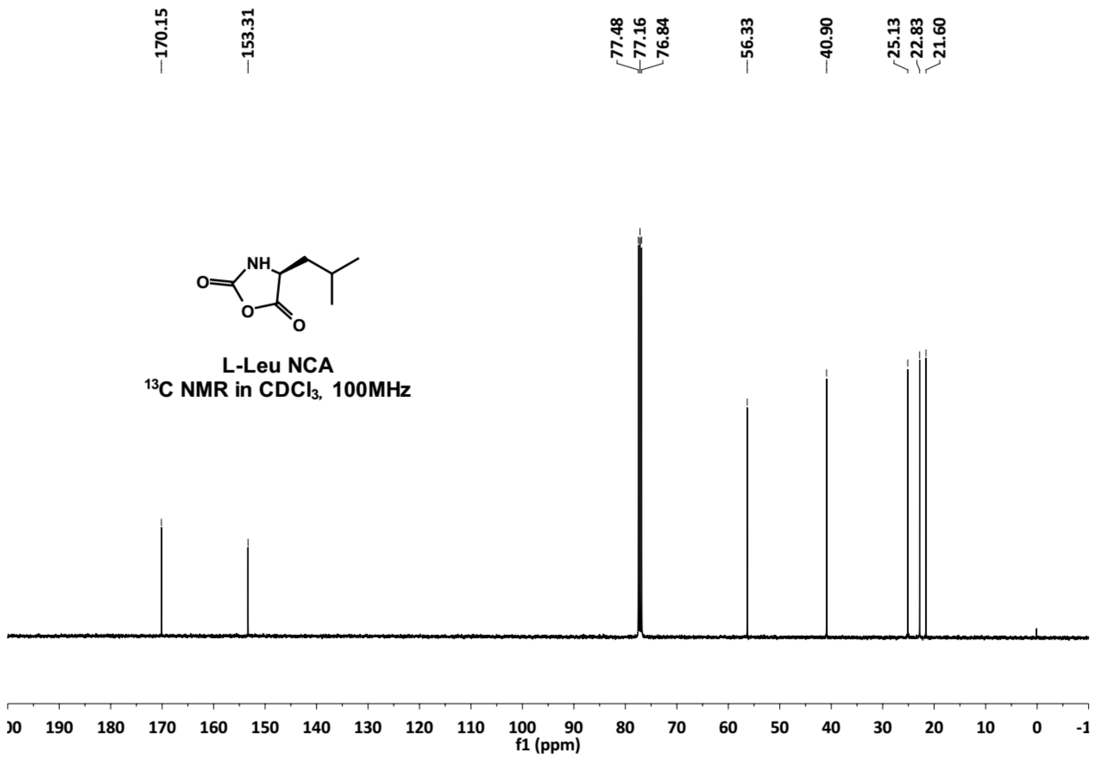

Supplementary Figure 20: ^{13}C NMR for L-Leu NCA in $CDCl_3$, 100MHz.

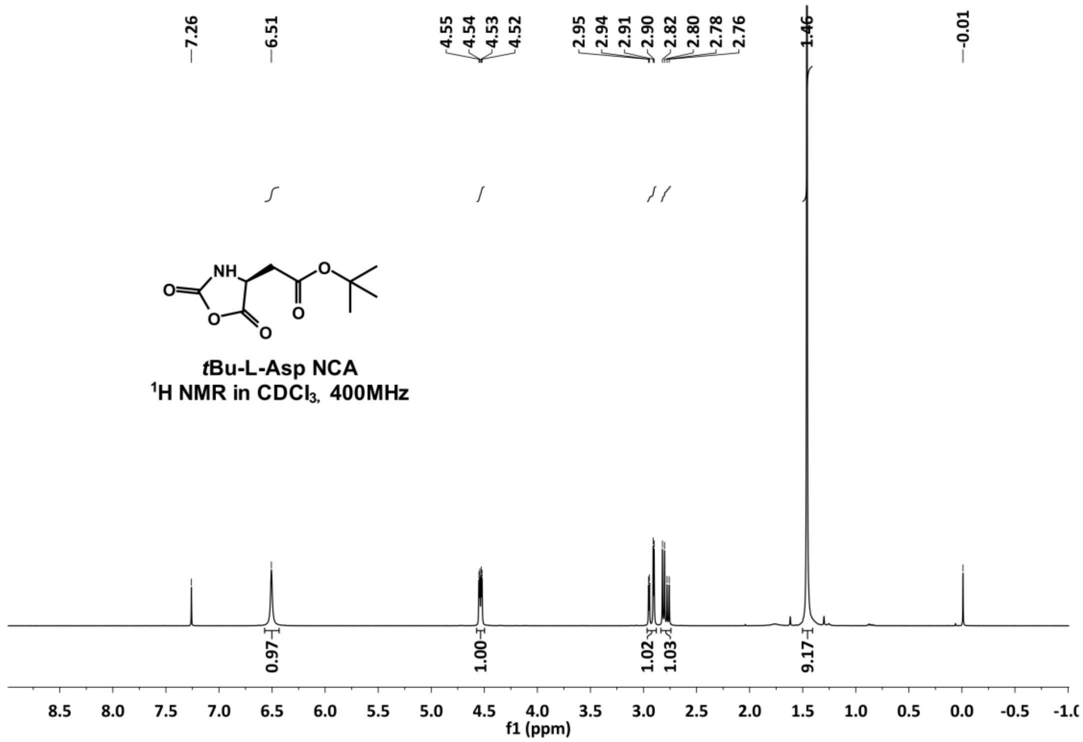

Supplementary Figure 21: ^{1}H NMR for *t*Bu-L-Asp NCA in $CDCl_3$, 400MHz.

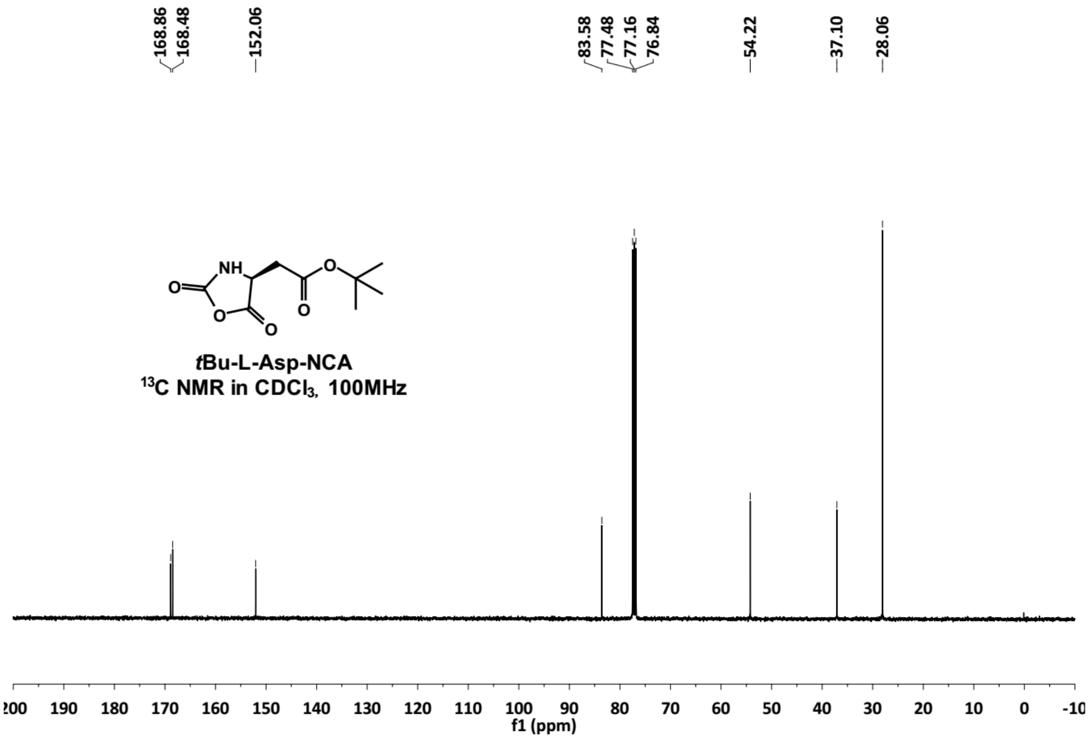

Supplementary Figure 22: ^{13}C NMR for *t*Bu-L-Asp NCA in $CDCl_3$, 100MHz.

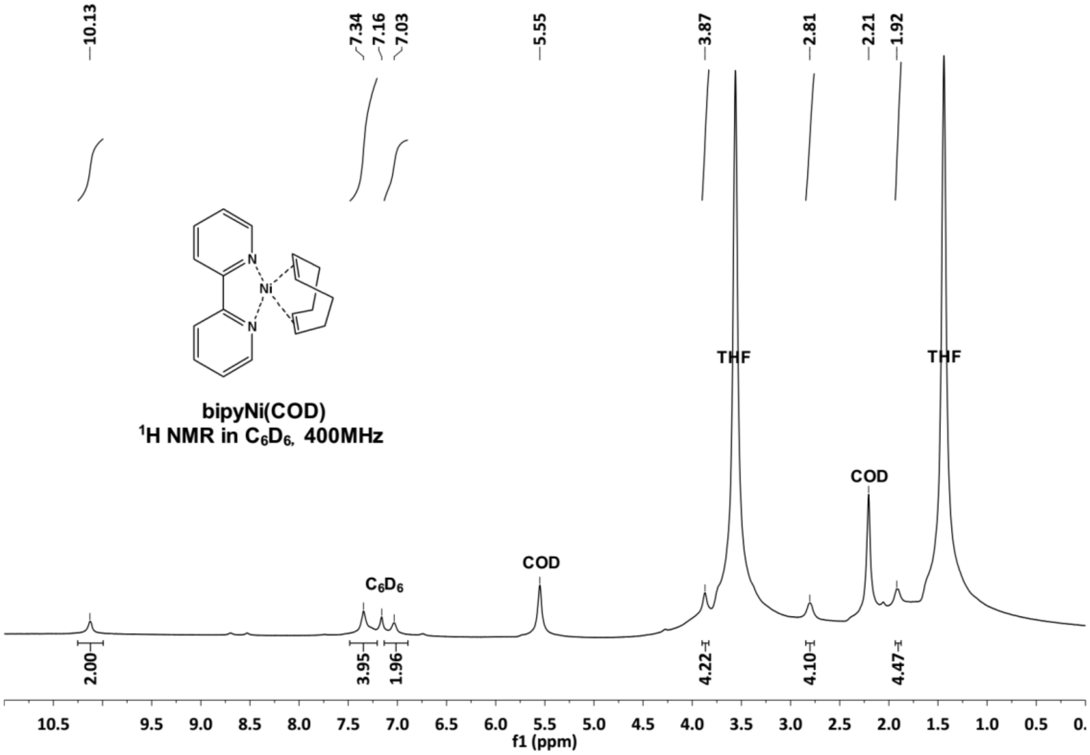

Supplementary Figure 23: ^{1}H NMR for bipyNi(COD) in THF (C_6D_6, 400MHz).

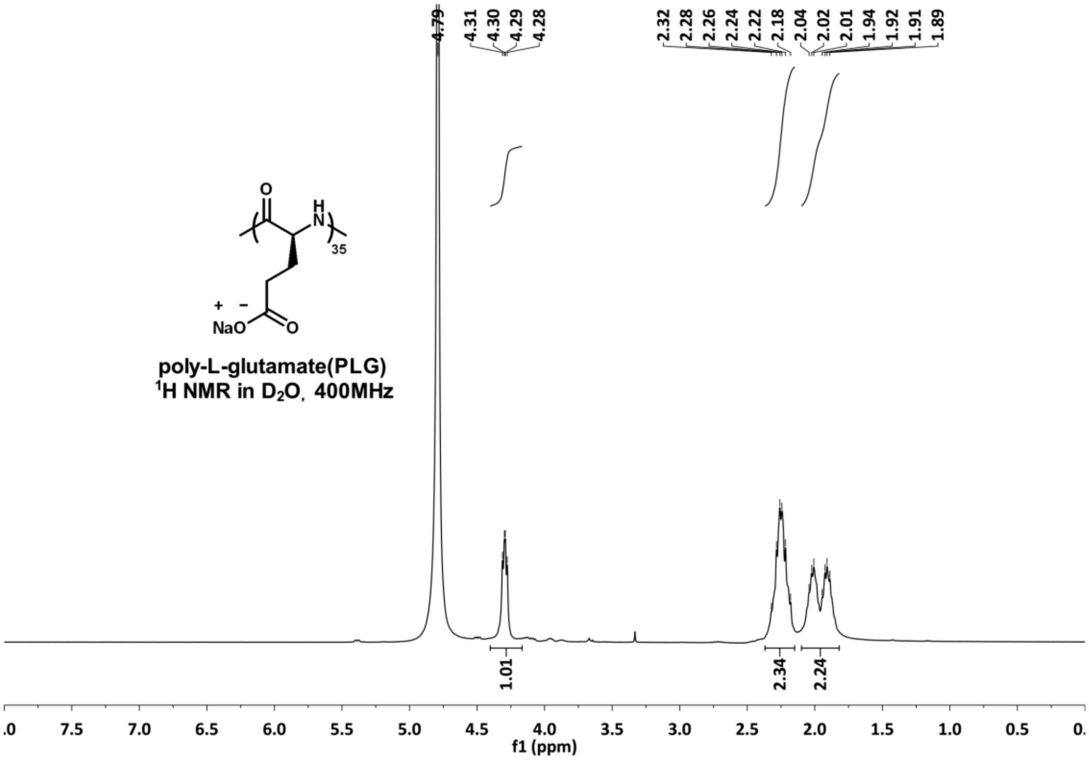

Supplementary Figure 24: representative ^{1}H NMR for poly-L-glutamate (PLG) in D_2O, 400MHz.

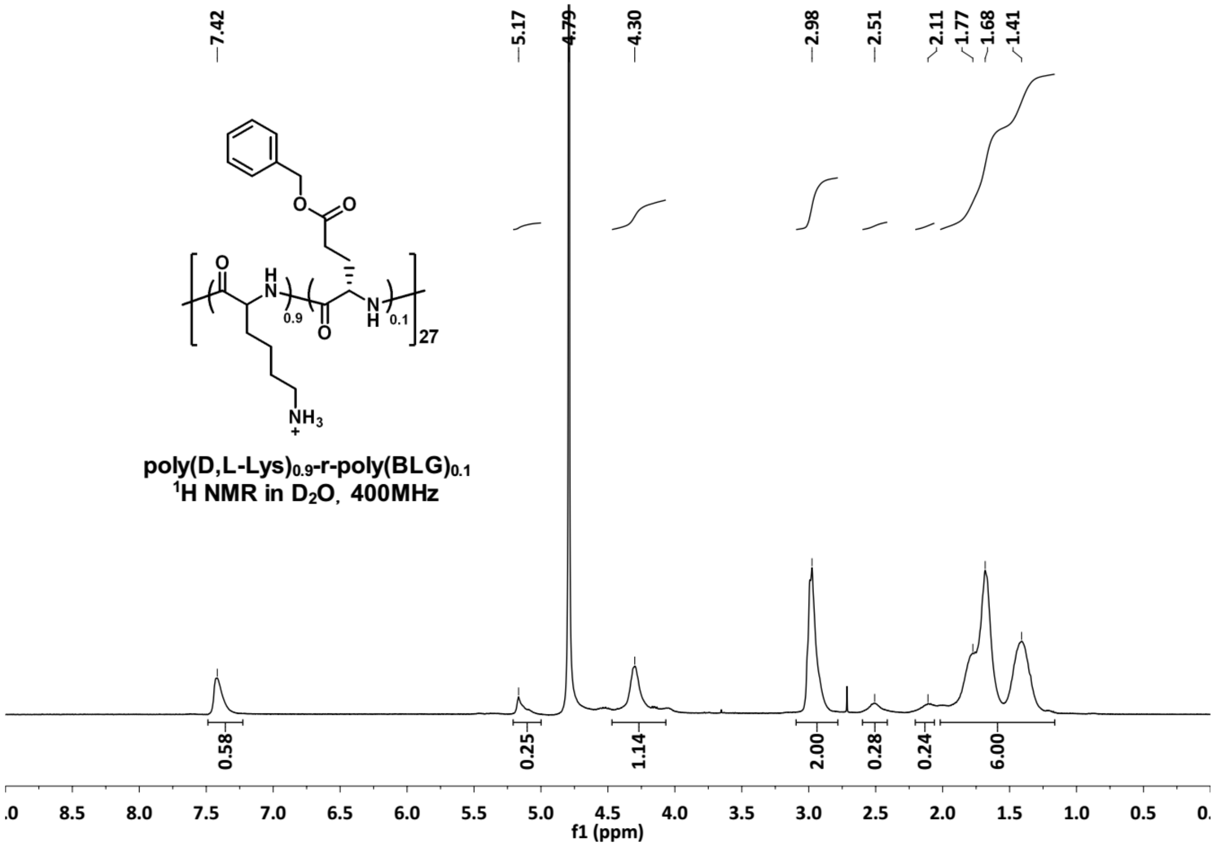

Supplementary Figure 25: ¹H NMR for poly(D,L-Lys)$_{0.9}$-r-poly(BLG)$_{0.1}$ prepared from LiHMDS-initiated polymerizaton of a mixture 9:1 D,L-Lys NCA:BLG NCA, (D_2O, 400MHz).

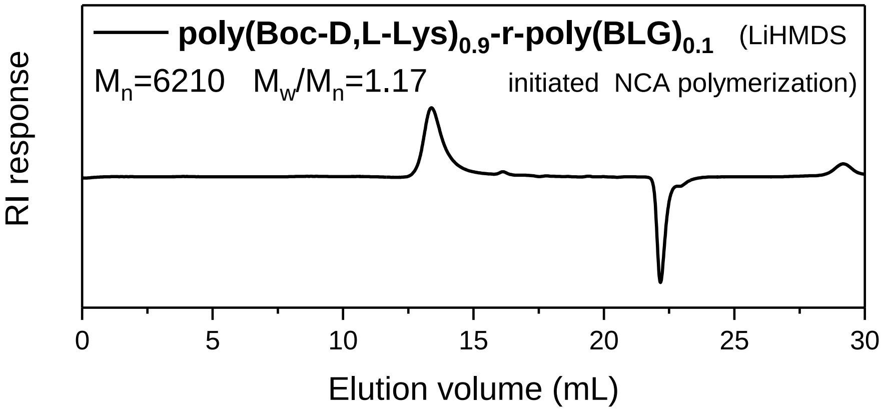

Supplementary Figure 26: GPC traces for poly(Boc-D,L-Lys)$_{0.9}$-r-poly(BLG)$_{0.1}$ prepared from LiHMDS-initiated polymerizaton of a mixture 9:1 Boc-D,L-Lys NCA:BLG NCA; DMF was used as the mobile phase.

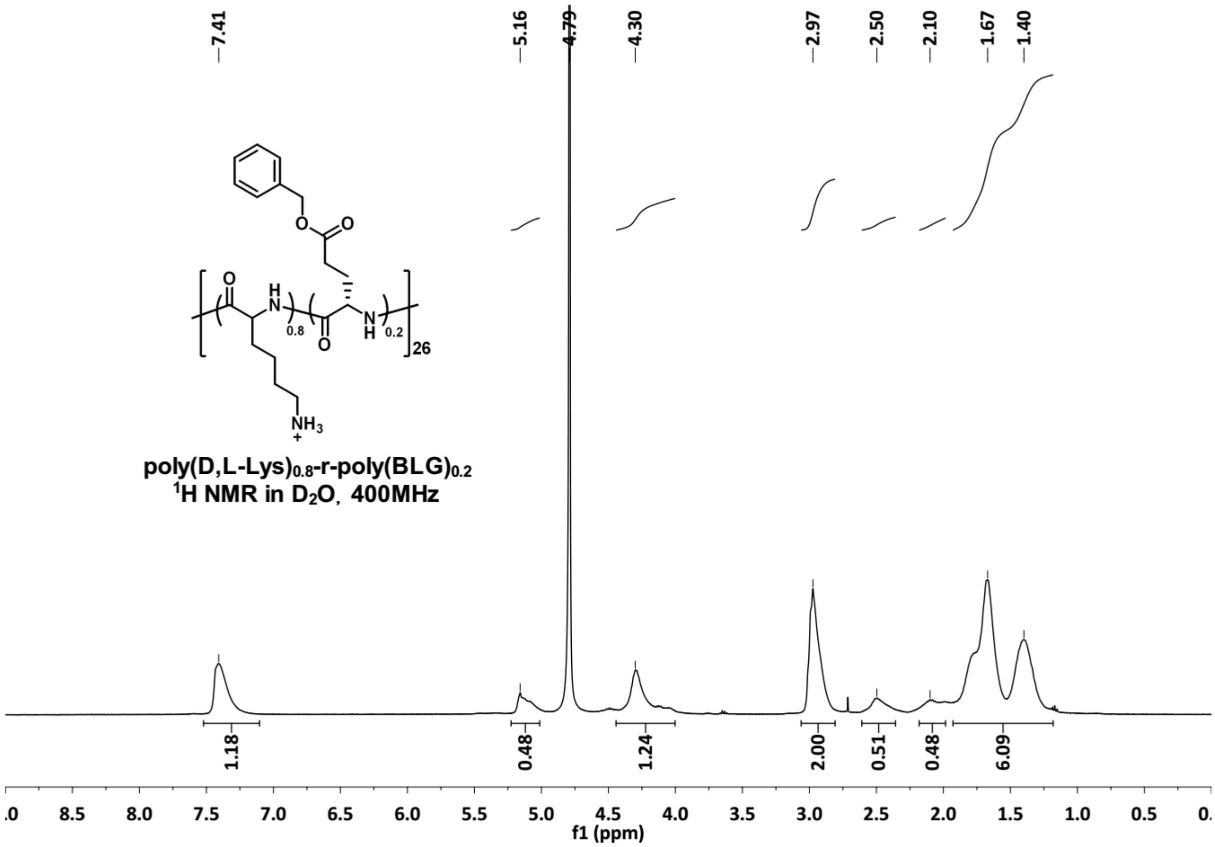

Supplementary Figure 27: ¹H NMR for poly(D,L-Lys)$_{0.8}$-r-poly(BLG)$_{0.2}$ prepared from LiHMDS-initiated polymerizaton of a mixture 8:2 D,L-Lys NCA:BLG NCA, (D_2O, 400MHz).

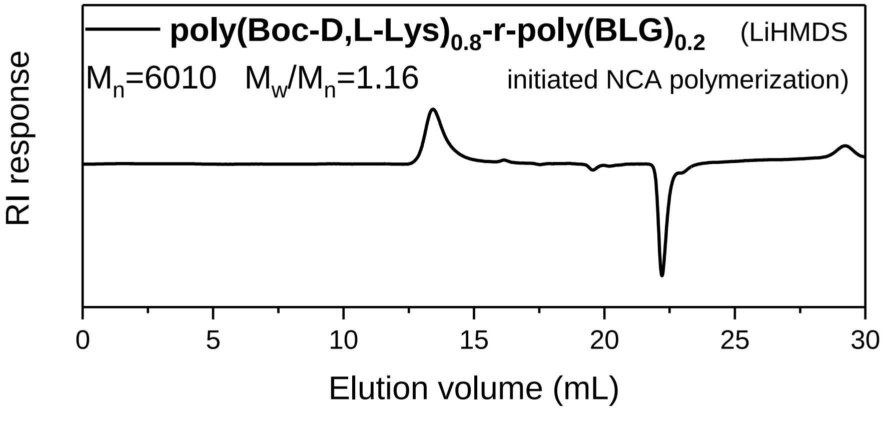

Supplementary Figure 28: GPC traces for poly(Boc-D,L-Lys)$_{0.8}$-r-poly(BLG)$_{0.2}$ prepared from LiHMDS-initiated polymerizaton of a mixture 8:2 Boc-D,L-Lys NCA:BLG NCA; DMF was used as the mobile phase.

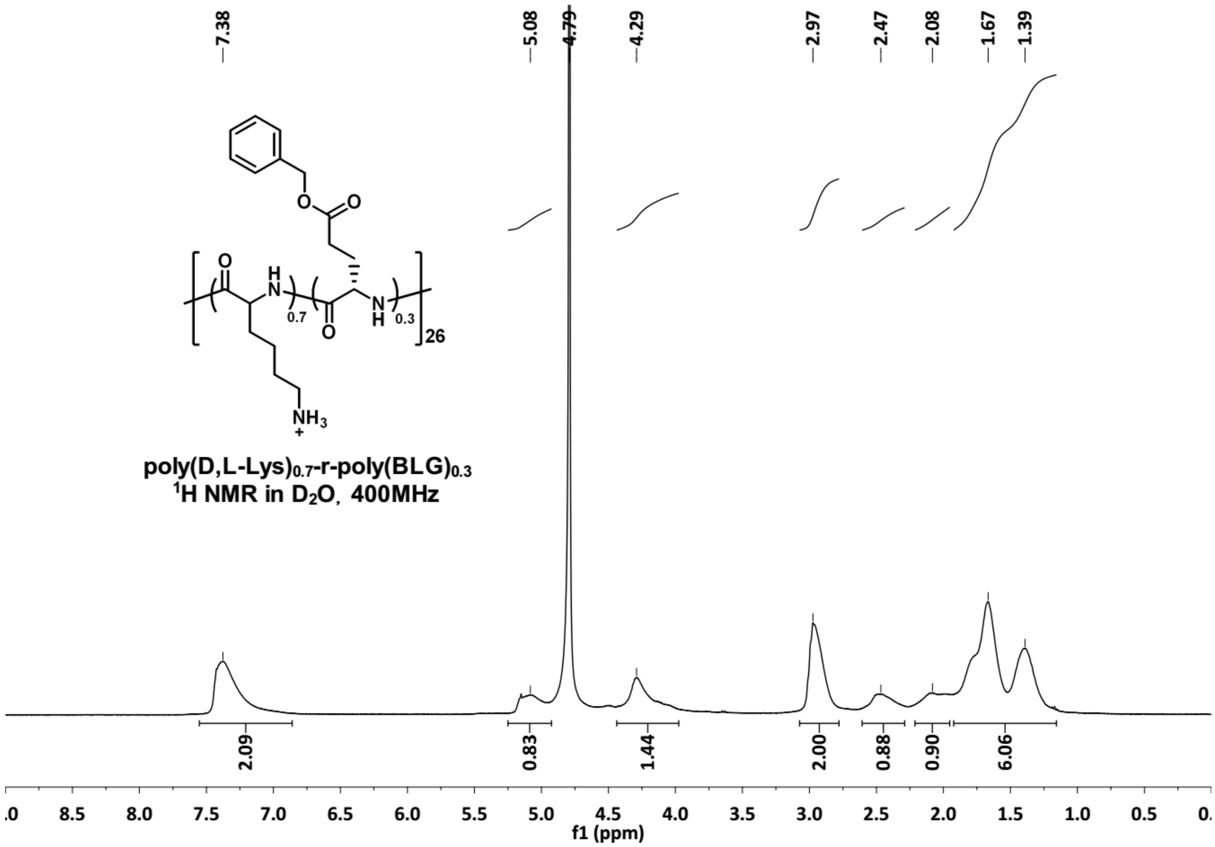

Supplementary Figure 29: ^{1}H NMR for poly(D,L-Lys)$_{0.7}$-r-poly(BLG)$_{0.3}$ prepared from LiHMDS-initiated polymerizaton of a mixture 7:3 D,L-Lys NCA:BLG NCA, (D_2O, 400MHz).

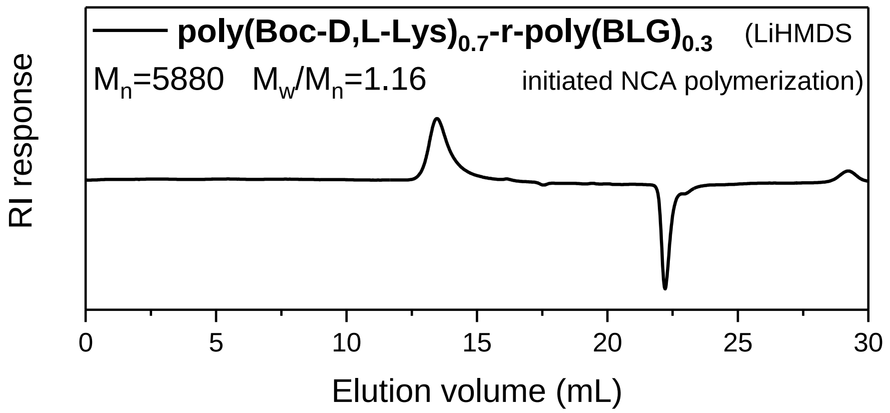

Supplementary Figure 30: GPC traces for poly(Boc-D,L-Lys)$_{0.7}$-r-poly(BLG)$_{0.3}$ prepared from LiHMDS-initiated polymerizaton of a mixture 7:3 Boc-D,L-Lys NCA:BLG NCA; DMF was used as the mobile phase.

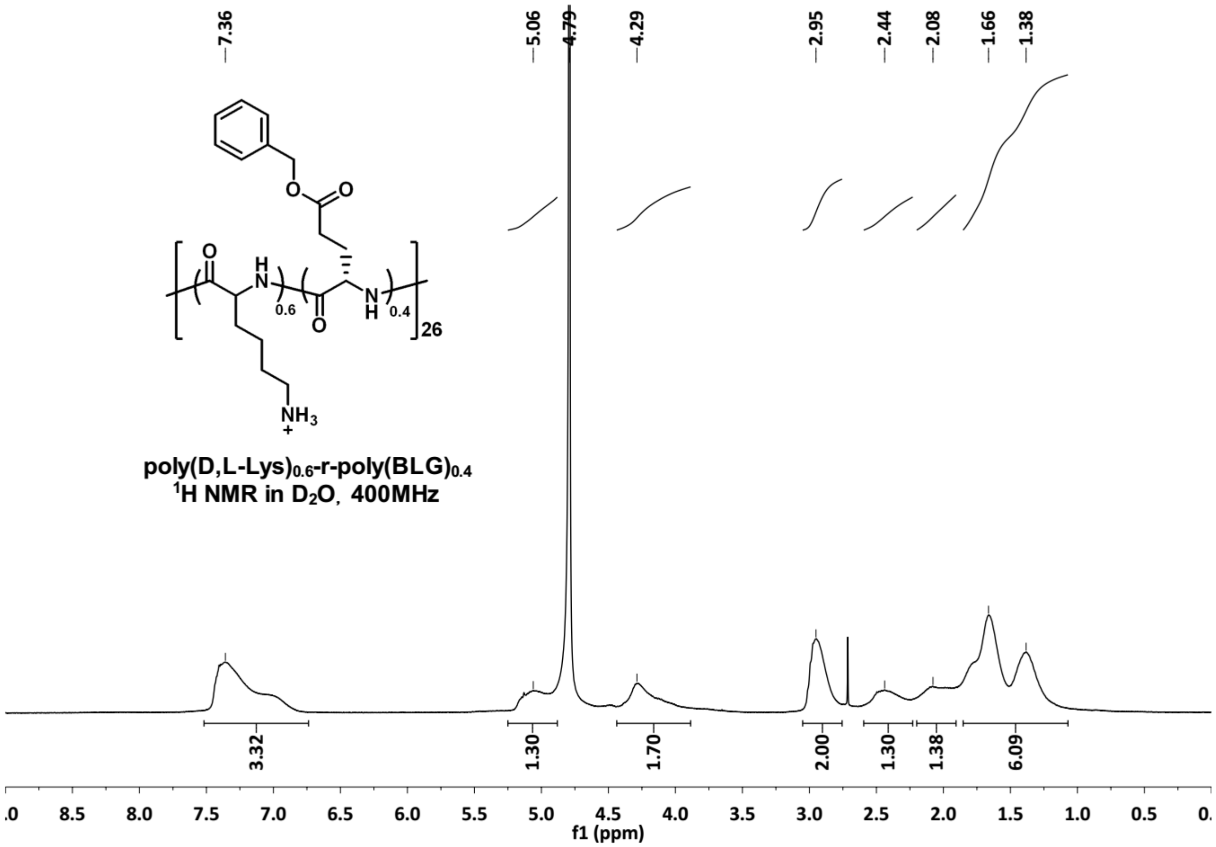

Supplementary Figure 31: ^{1}H NMR for poly(D,L-Lys)$_{0.6}$-r-poly(BLG)$_{0.4}$ prepared from LiHMDS-initiated polymerizaton of a mixture 6:4 D,L-Lys NCA:BLG NCA, (D_2O, 400MHz).

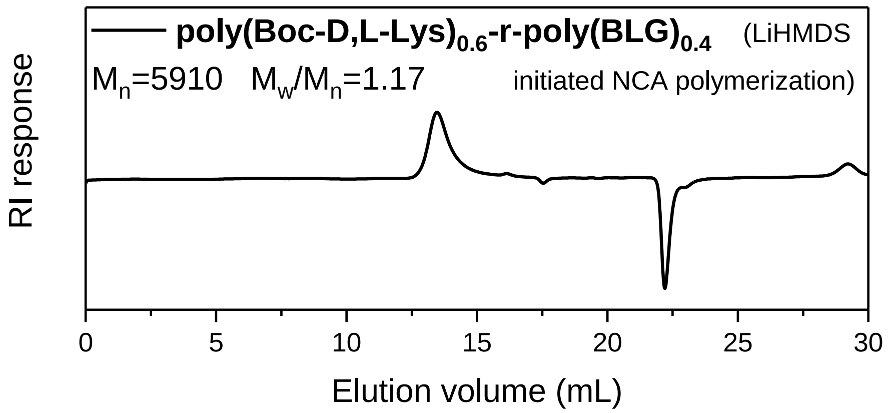

Supplementary Figure 32: GPC traces for poly(Boc-D,L-Lys)$_{0.6}$-r-poly(BLG)$_{0.4}$ prepared from LiHMDS-initiated polymerizaton of a mixture 6:4 Boc-D,L-Lys NCA:BLG NCA; DMF was used as the mobile phase.

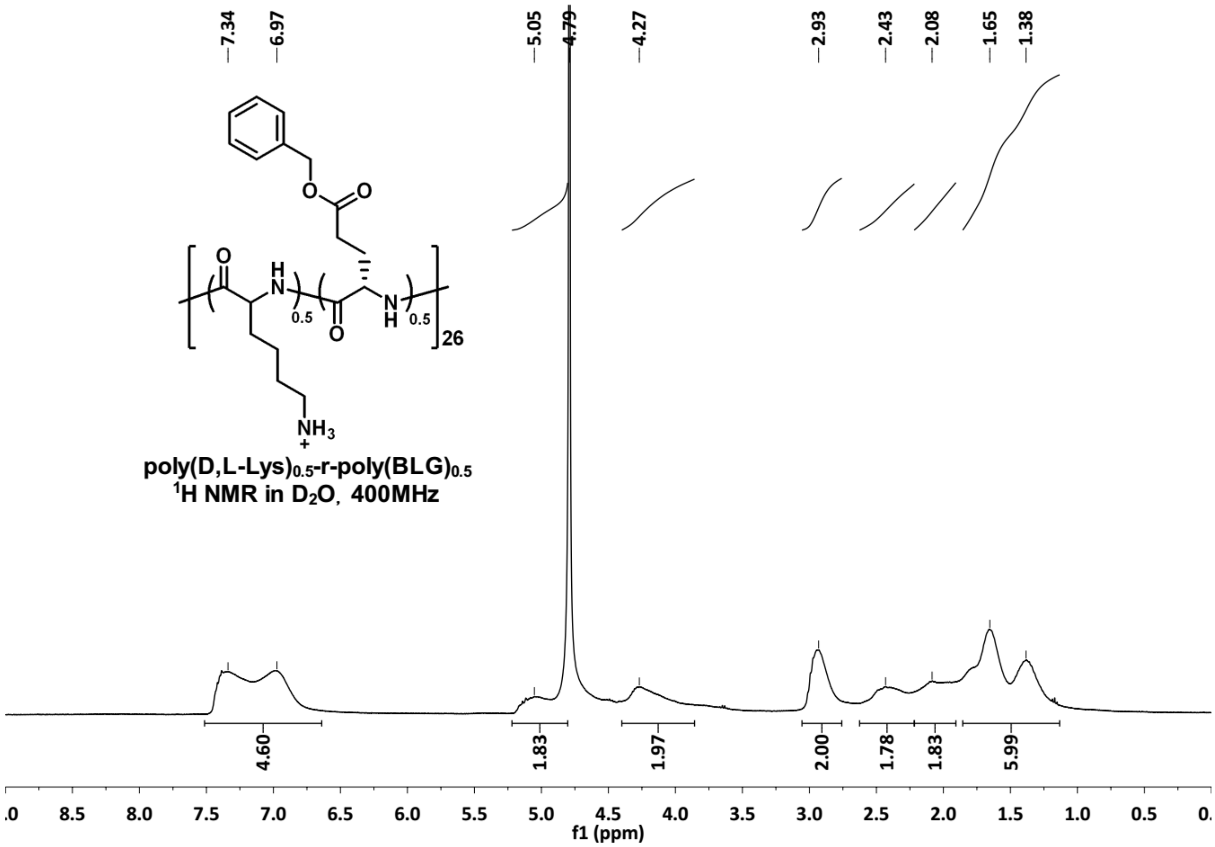

Supplementary Figure 33: ^{1}H NMR for poly(D,L-Lys)$_{0.5}$-r-poly(BLG)$_{0.5}$ prepared from LiHMDS-initiated polymerizaton of a mixture 5:5 D,L-Lys NCA:BLG NCA, (D_2O, 400MHz).

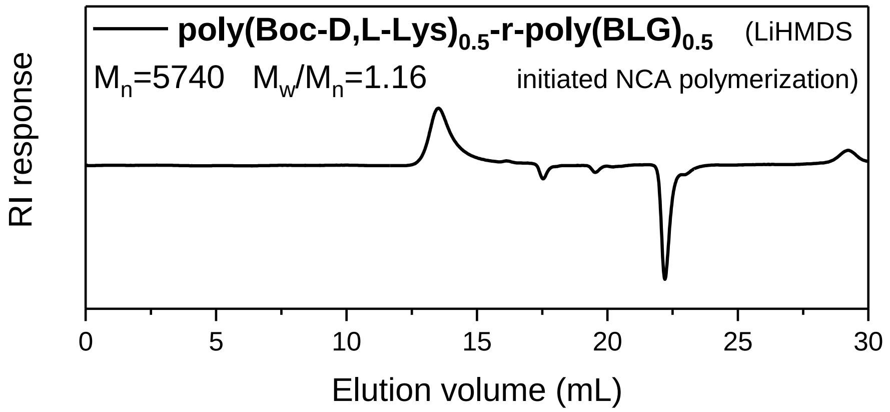

Supplementary Figure 34: GPC traces for poly(Boc-D,L-Lys)$_{0.5}$-r-poly(BLG)$_{0.5}$ prepared from LiHMDS-initiated polymerizaton of a mixture 5:5 Boc-D,L-Lys NCA:BLG NCA; DMF was used as the mobile phase.

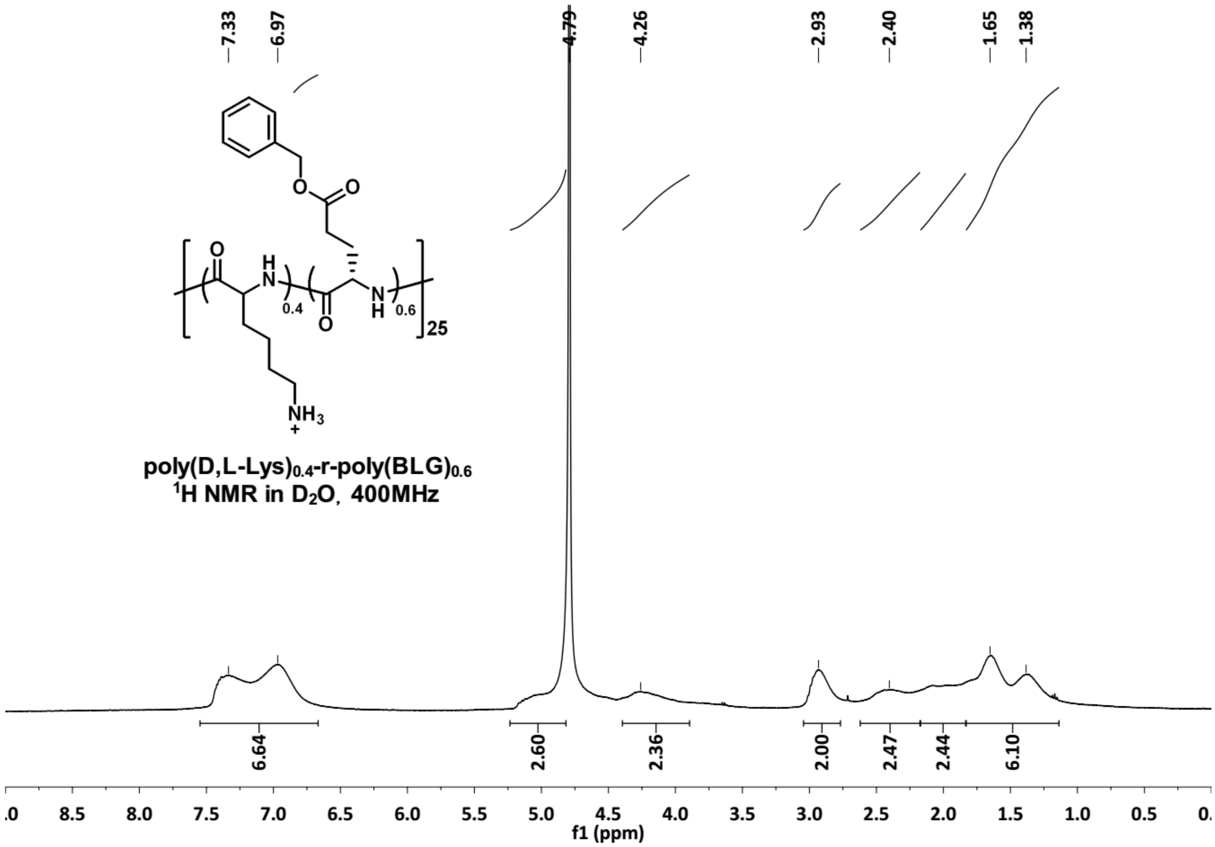

Supplementary Figure 35: ^{1}H NMR for poly(D,L-Lys)$_{0.4}$-r-poly(BLG)$_{0.6}$ prepared from LiHMDS-initiated polymerizaton of a mixture 4:6 D,L-Lys NCA:BLG NCA, (D_2O, 400MHz).

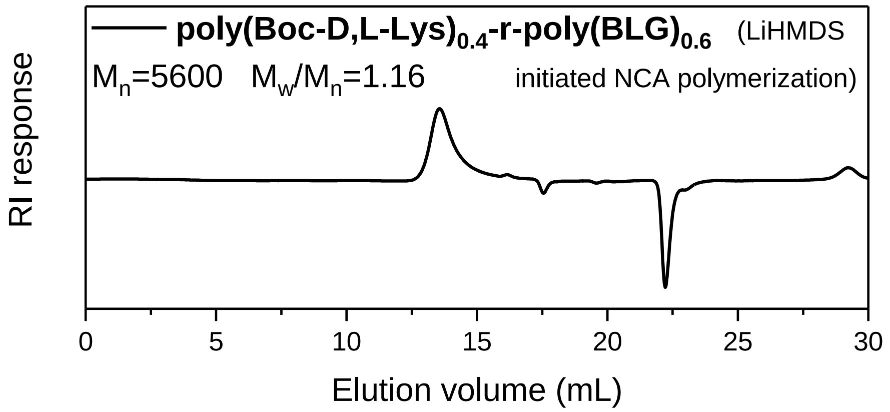

Supplementary Figure 36: GPC traces for poly(Boc-D,L-Lys)$_{0.4}$-r-poly(BLG)$_{0.6}$ prepared from LiHMDS-initiated polymerizaton of a mixture 4:6 Boc-D,L-Lys NCA:BLG NCA; DMF was used as the mobile phase.

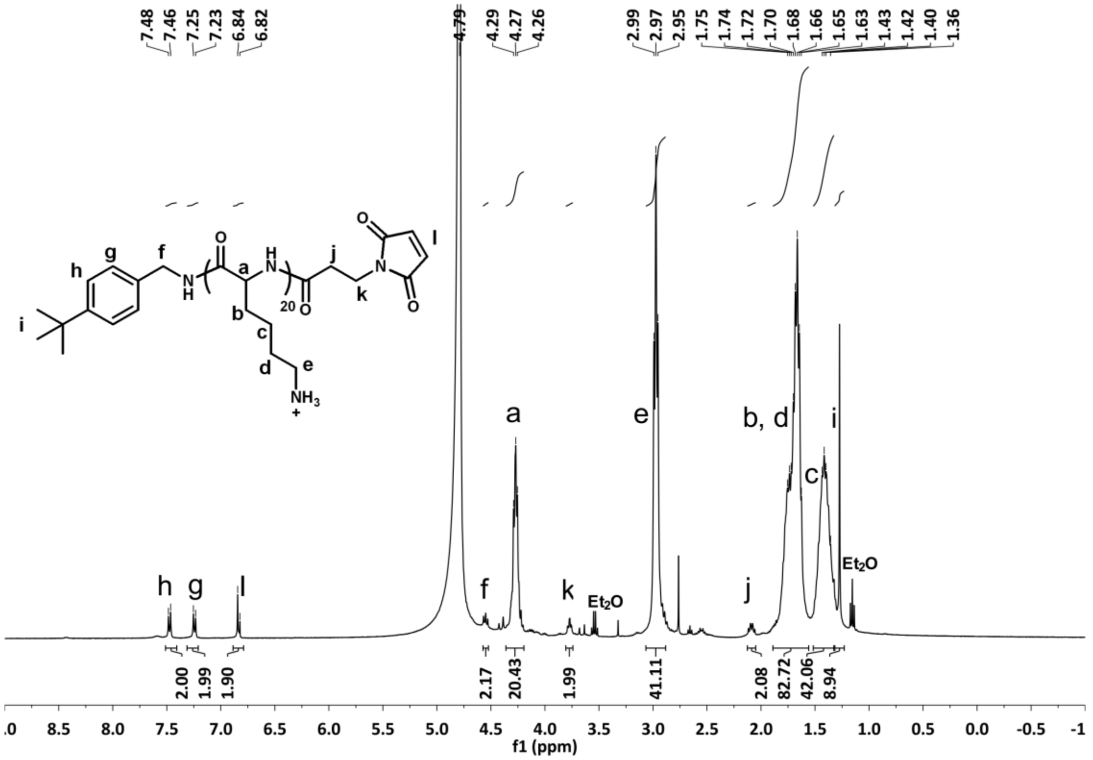

Supplementary Figure 37: ^{1}H NMR for C-terminus and N-terminus dual functionalized poly-D,L-Lys_{20} (D_2O, 400MHz).

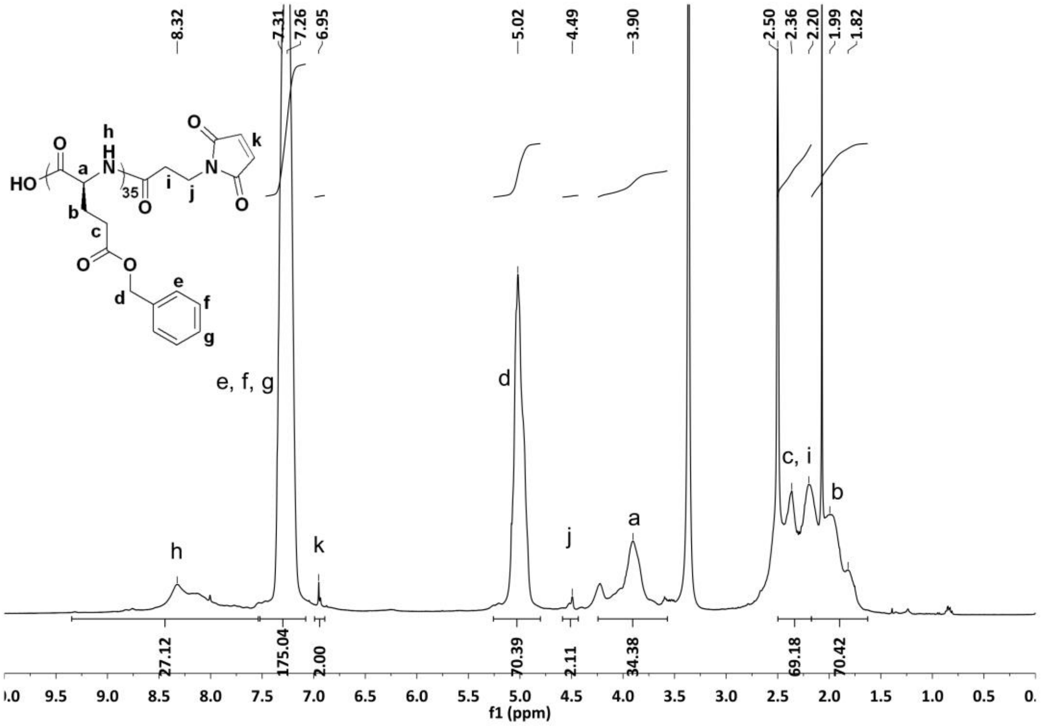

Supplementary Figure 38: ^{1}H NMR for N-terminus functionalized poly-BLG_{35} (DMSO-*d*6, 400MHz).

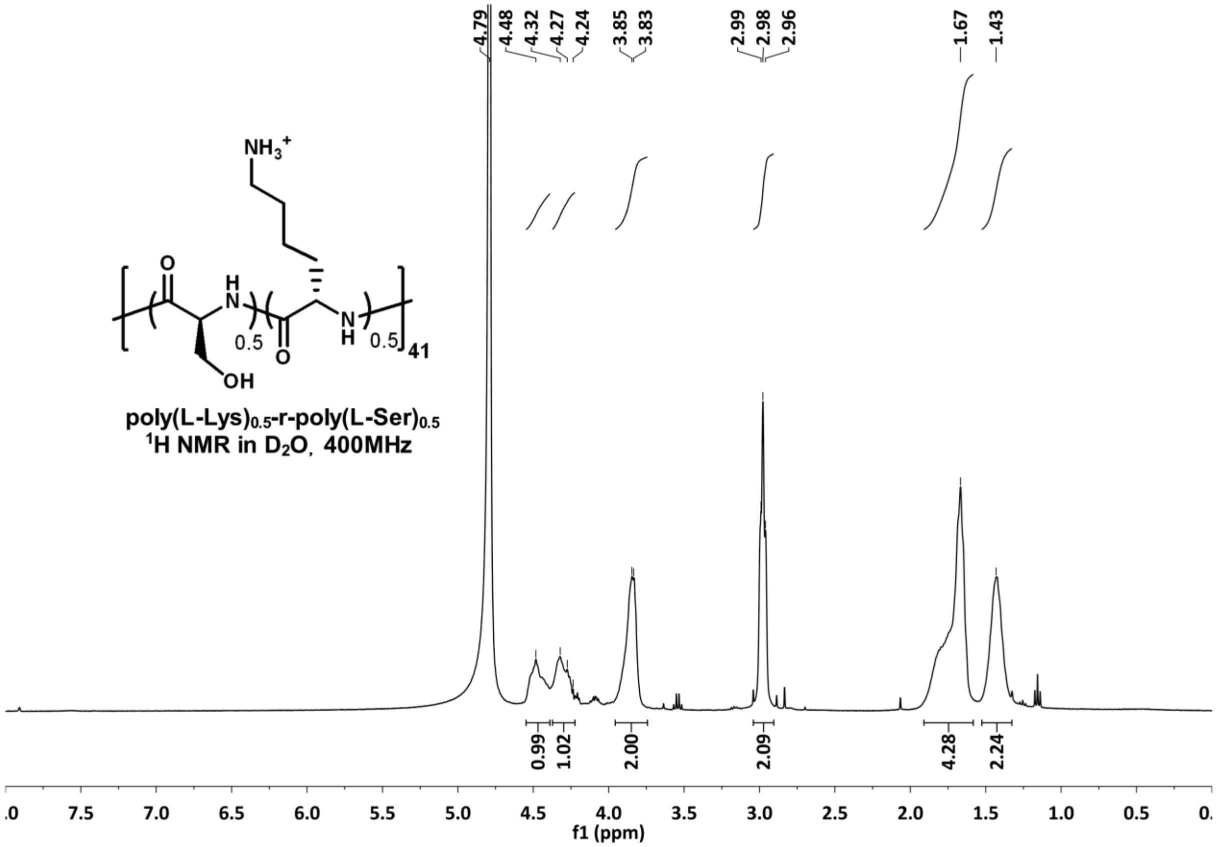

Supplementary Figure 39: ^{1}H NMR for poly(L-Lys)$_{0.5}$-r-poly(L-Ser)$_{0.5}$ (D_2O, 400MHz).

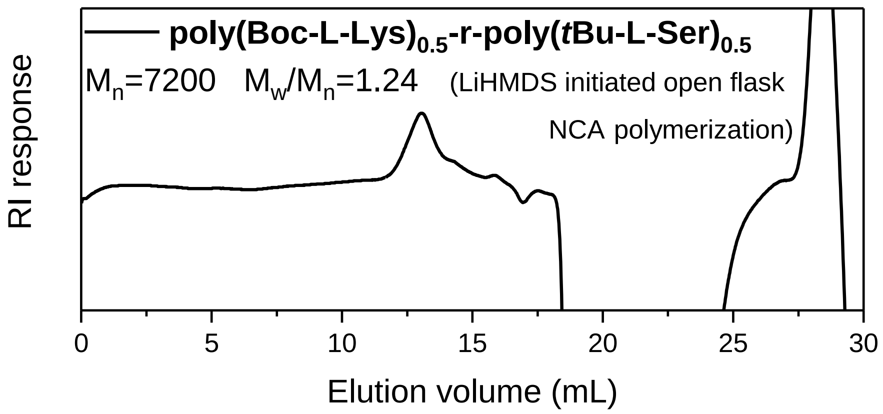

Supplementary Figure 40: GPC traces of poly(Boc-L-Lys)$_{0.5}$-r-poly(*t*Bu-L-Ser)$_{0.5}$ (LiHMDS-initiated open vessel NCA polymerization).

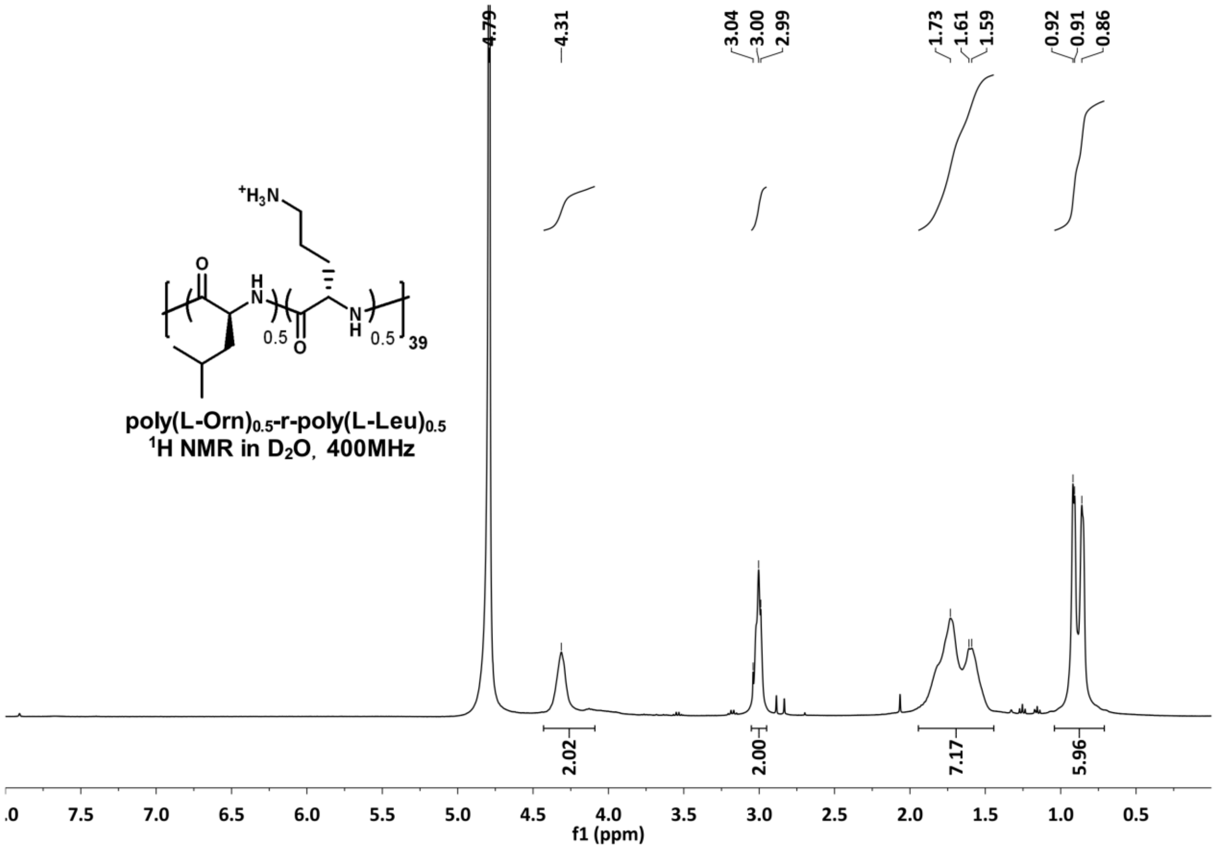

Supplementary Figure 41: ^{1}H NMR for poly(L-Orn)$_{0.5}$-r-poly(L-Leu)$_{0.5}$ (D_2O, 400MHz).

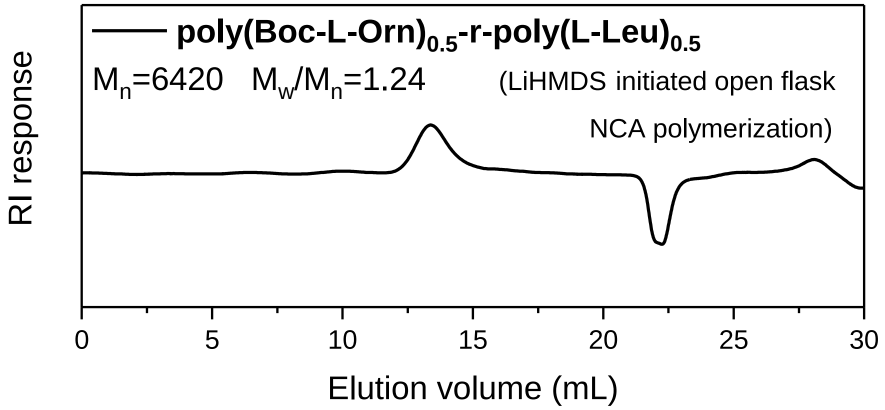

Supplementary Figure 42: GPC traces of 1:1 poly(Boc-L-Orn)$_{0.5}$-r-poly(L-Leu)$_{0.5}$ (LiHMDS-initiated open vessel NCA polymerization).

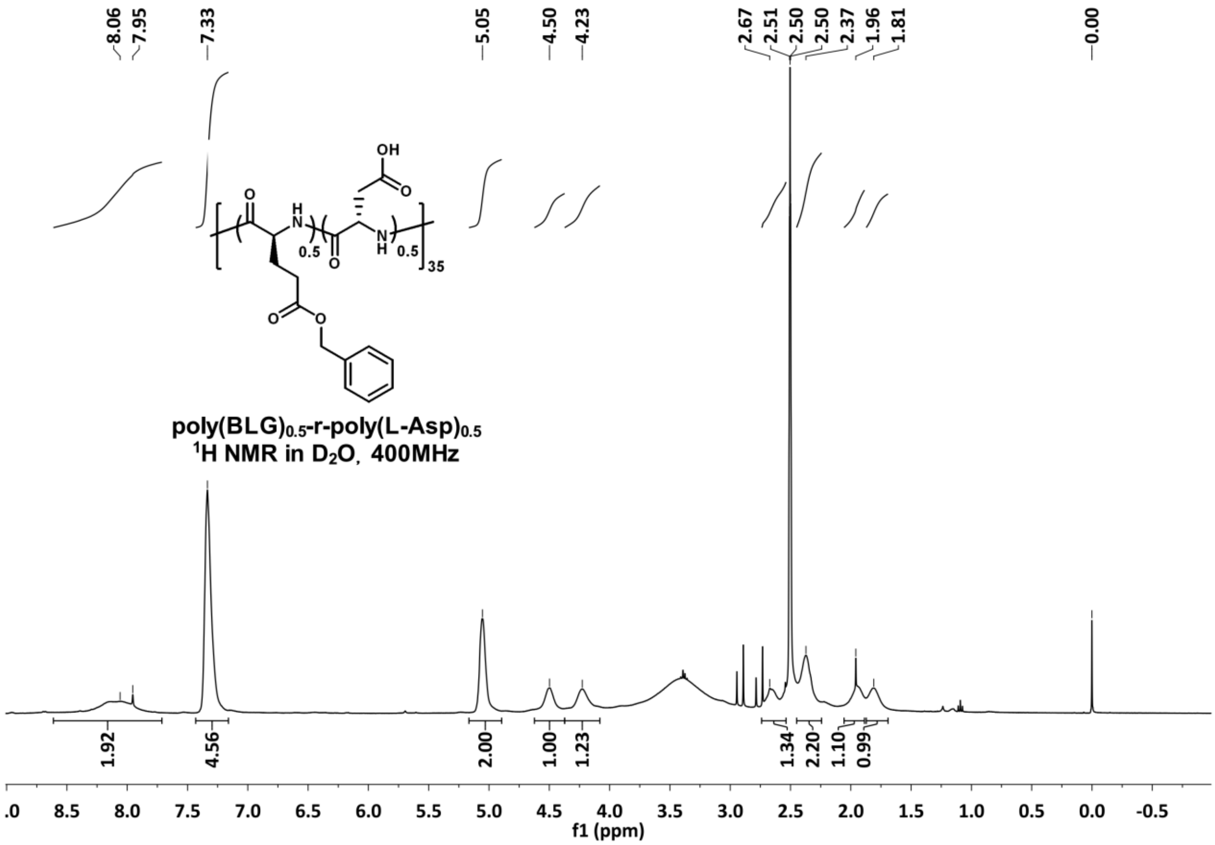

Supplementary Figure 43: ^{1}H NMR for poly(BLG)$_{0.5}$-r-poly(L-Asp)$_{0.5}$ (D_2O, 400MHz).

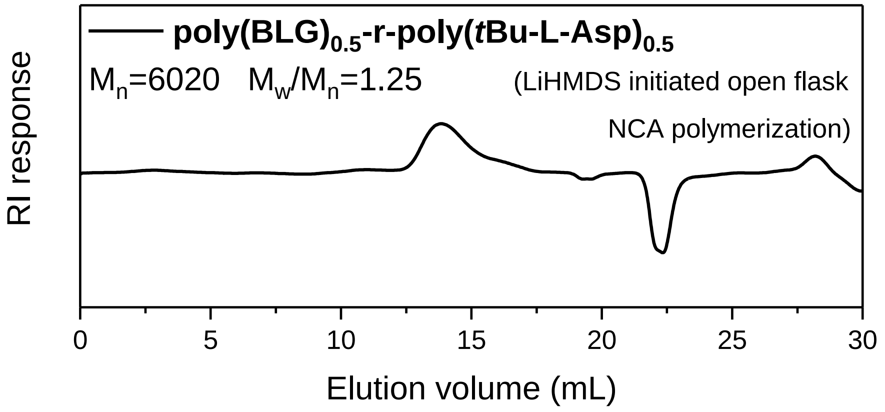

Supplementary Figure 44: GPC traces of poly(BLG)$_{0.5}$-r-poly(*t*Bu L-Asp)$_{0.5}$ (LiHMDS initiated open vessel NCA polymerization).

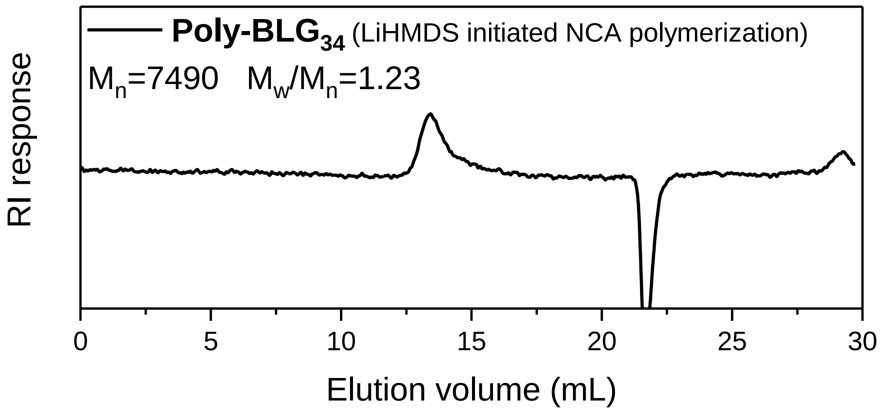

Supplementary Figure 45: GPC traces of poly-BLG_{34} (LiHMDS initiated NCA polymerization).

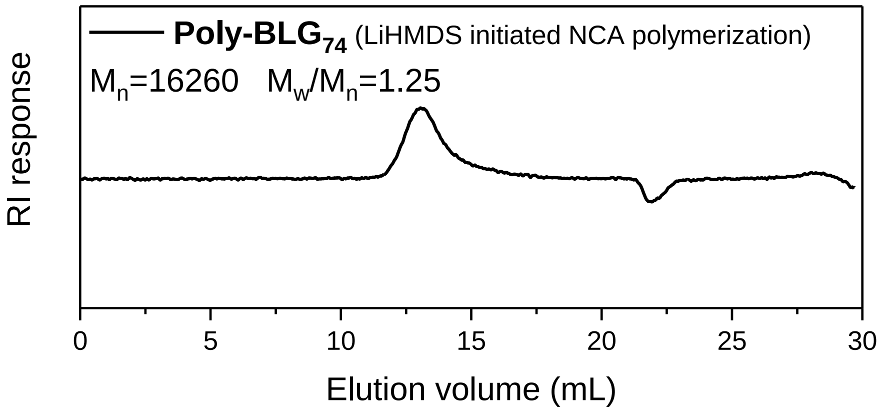

Supplementary Figure 46: GPC traces of poly-BLG_{74} (LiHMDS initiated NCA polymerization).

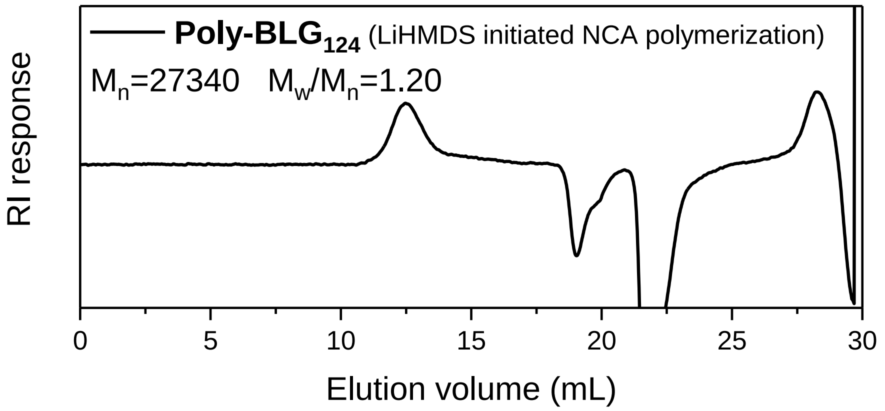

Supplementary Figure 47: GPC traces of poly-BLG_{124} (LiHMDS initiated NCA polymerization).

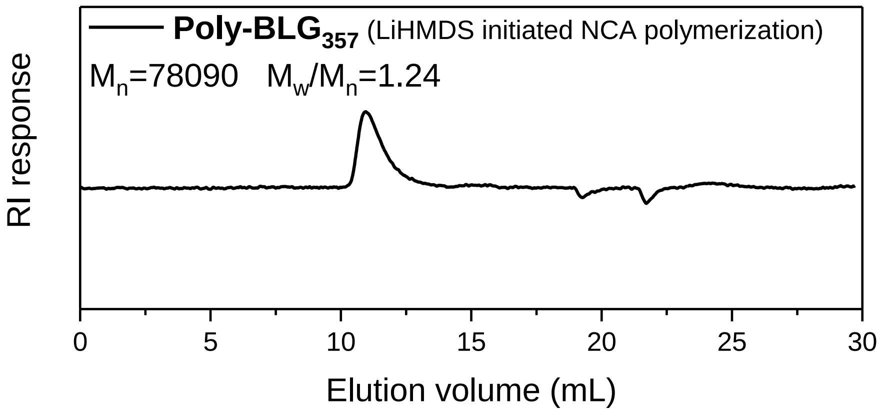

Supplementary Figure 48: GPC traces of poly-BLG_{357} (LiHMDS initiated NCA polymerization).

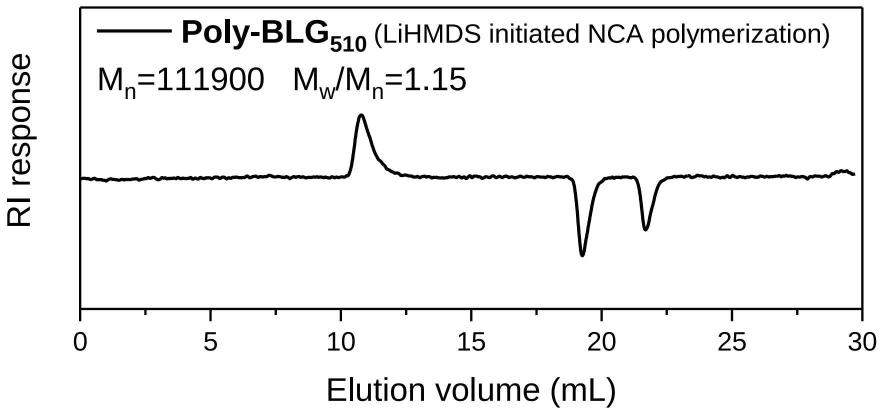

Supplementary Figure 49: GPC traces of poly-BLG_{510} (LiHMDS initiated NCA polymerization).

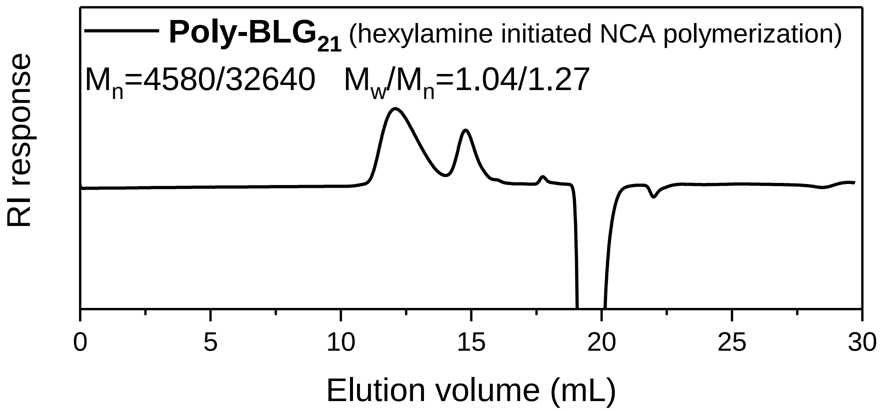

Supplementary Figure 50: GPC traces of poly-BLG_{21} (hexylamine initiated NCA polymerization).

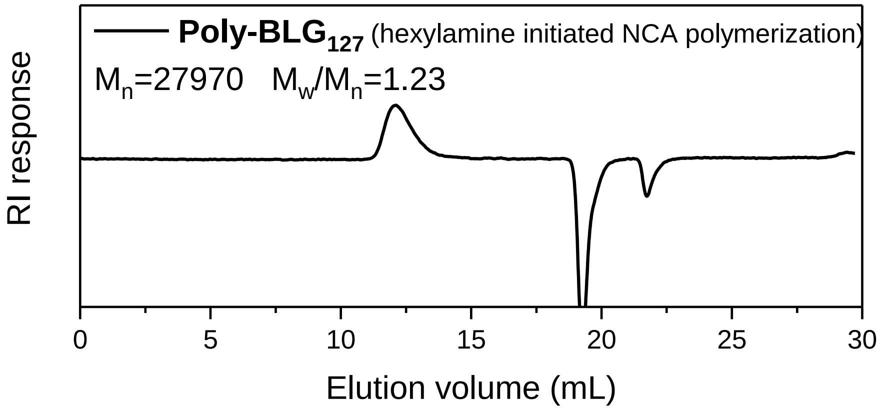

Supplementary Figure 51: GPC traces of poly-BLG_{127} (hexylamine initiated NCA polymerization).

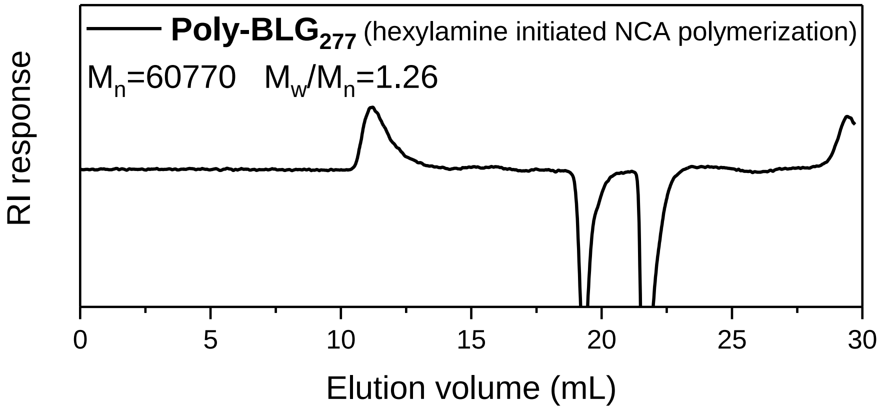

Supplementary Figure 52: GPC traces of poly-BLG_{277} (hexylamine initiated NCA polymerization).

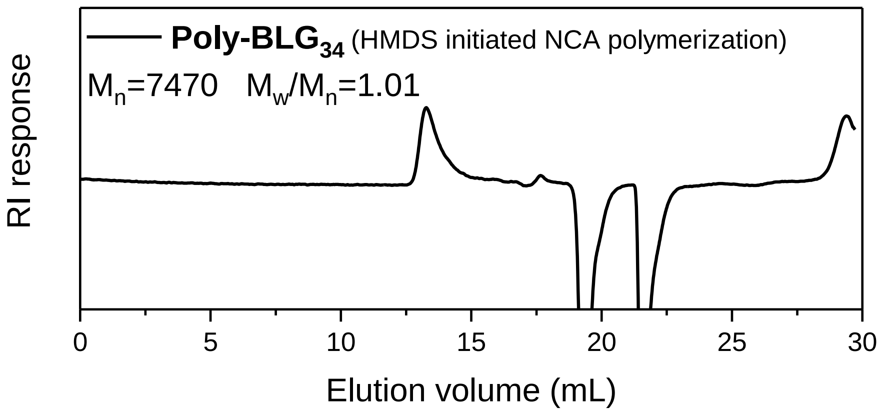

Supplementary Figure 53: GPC traces of poly-BLG_{34} (HMDS initiated NCA polymerization).

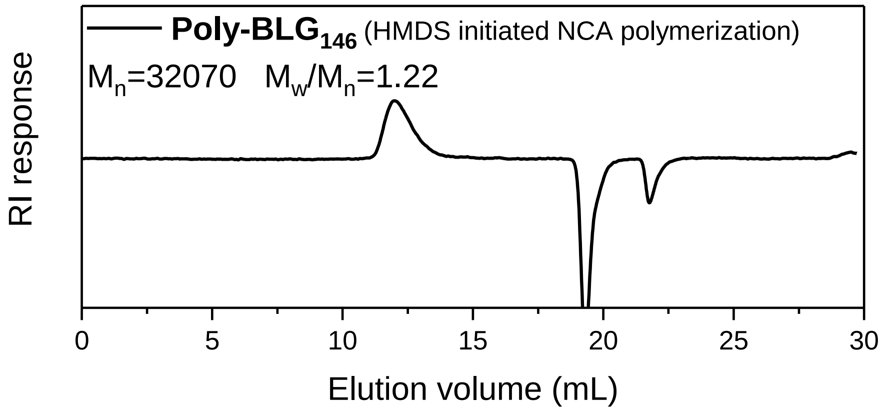

Supplementary Figure 54: GPC traces of poly-BLG_{146} (HMDS initiated NCA polymerization).

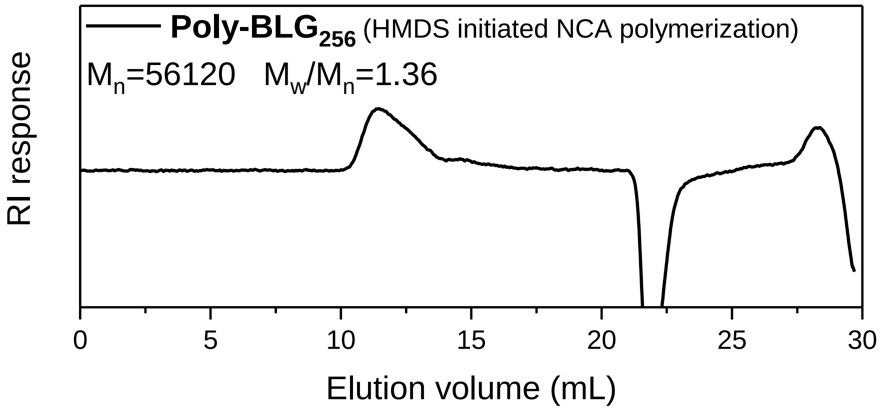

Supplementary Figure 55: GPC traces of poly-BLG_{256} (HMDS initiated NCA polymerization).

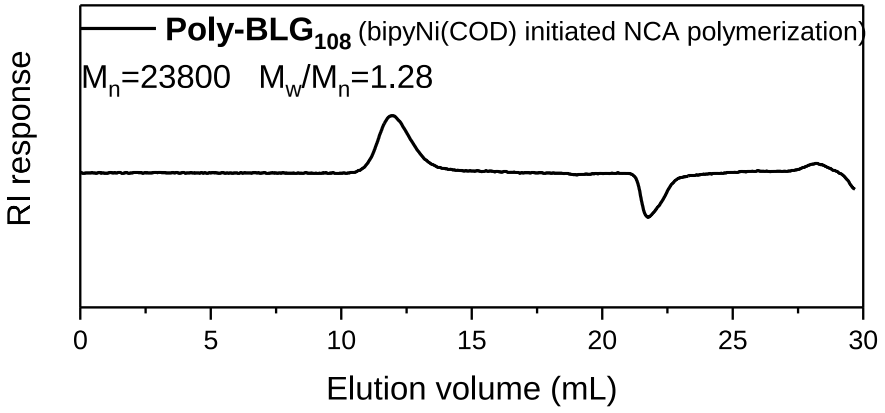

Supplementary Figure 56: GPC traces of poly-BLG_{108} (bipyNi(COD) initiated NCA polymerization).

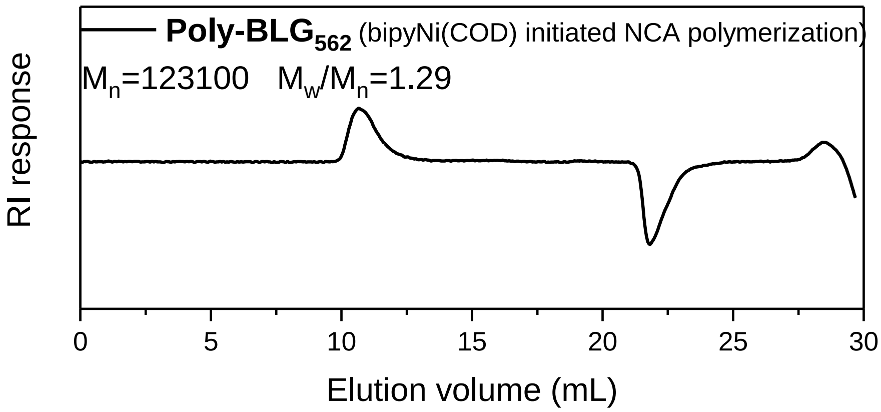

Supplementary Figure 57: GPC traces of poly-BLG_{562} (bipyNi(COD) initiated NCA polymerization).

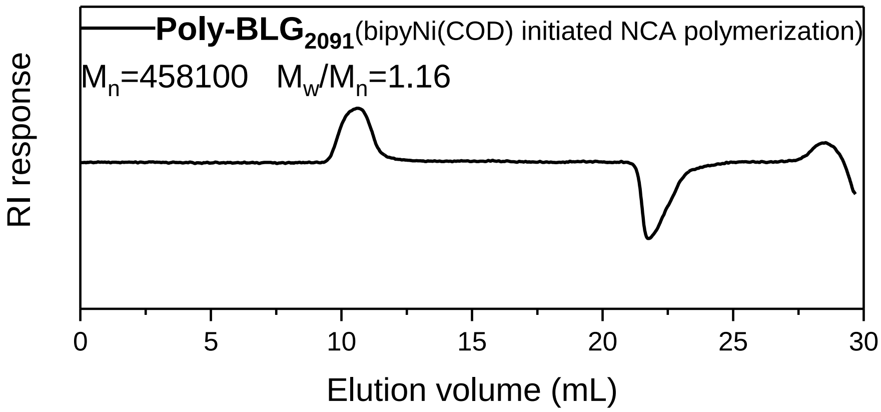

Supplementary Figure 58: GPC traces of poly-BLG_{2091} (bipyNi(COD) initiated NCA polymerization).

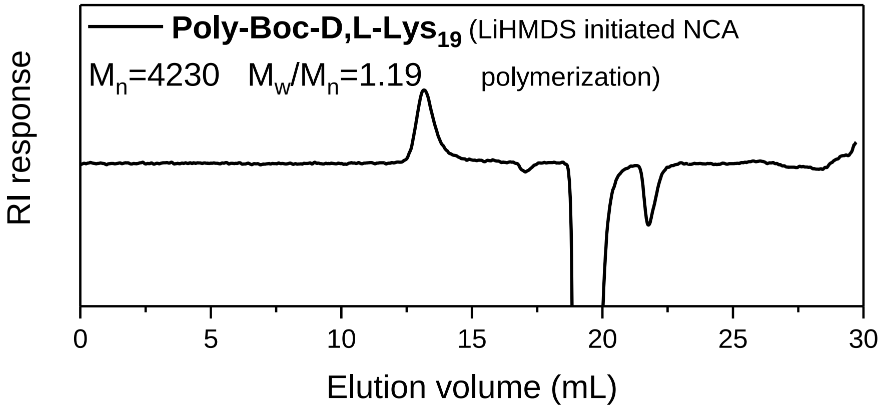

Supplementary Figure 59: GPC traces of poly-Boc-D,L-Lys_{19} (LiHMDS initiated NCA polymerization).

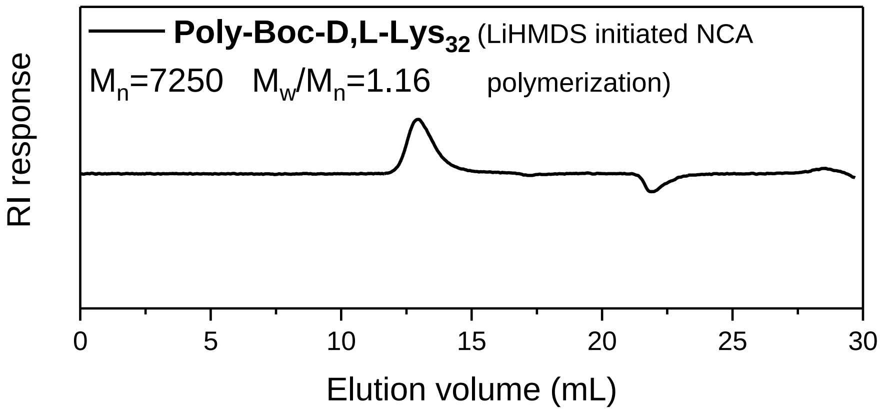

Supplementary Figure 60: GPC traces of poly-Boc-D,L-Lys_{32} (LiHMDS initiated NCA polymerization).

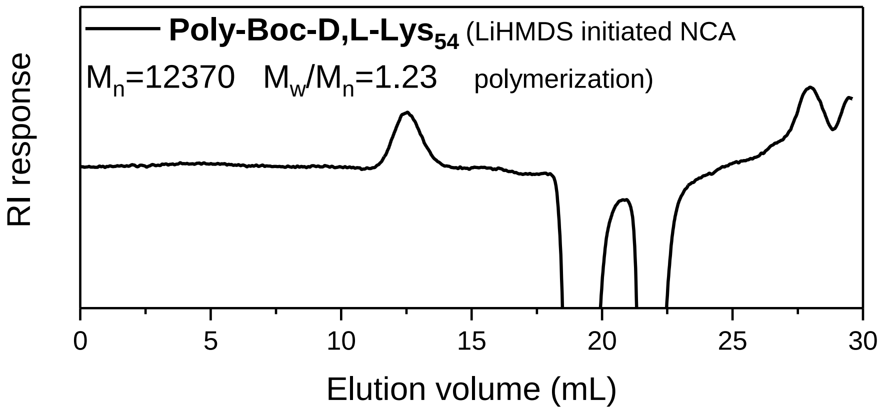

Supplementary Figure 61: GPC traces of poly-Boc-D,L-Lys_{54} (LiHMDS initiated NCA polymerization).

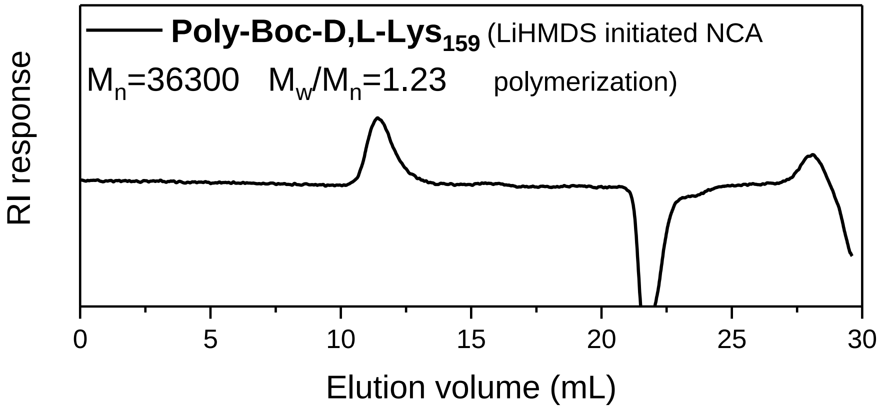

Supplementary Figure 62: GPC traces of poly-Boc-D,L-Lys_{159} (LiHMDS initiated NCA polymerization).

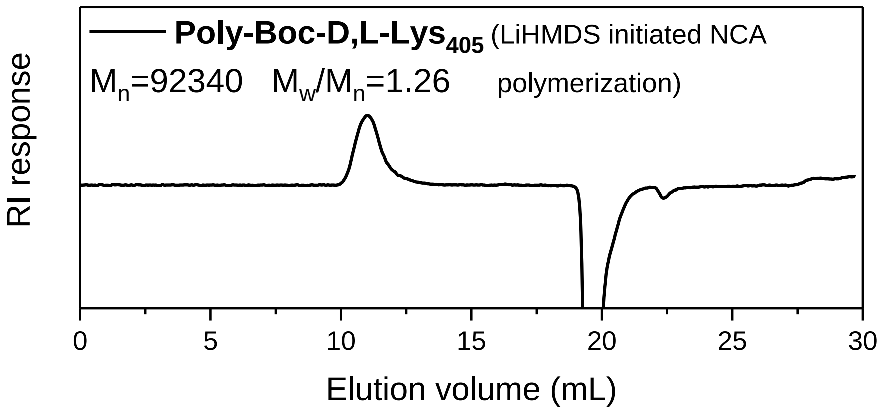

Supplementary Figure 63: GPC traces of poly-Boc-D,L-Lys_{405} (LiHMDS initiated NCA polymerization).

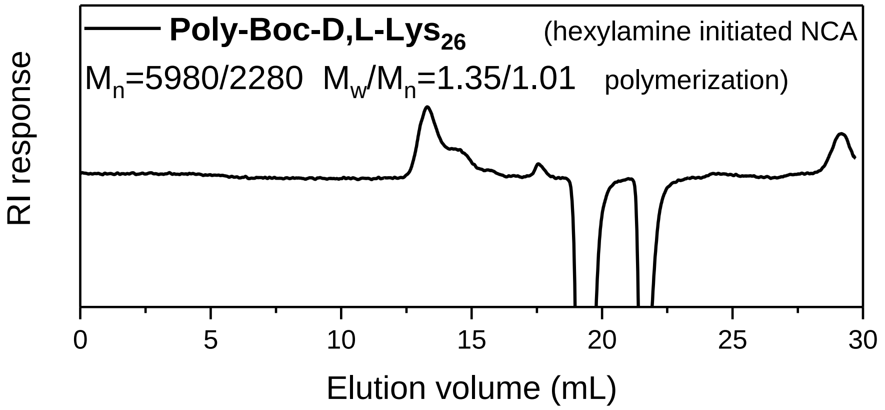

Supplementary Figure 64: GPC traces of poly-Boc-D,L-Lys_{26} (hexylamine initiated NCA polymerization).

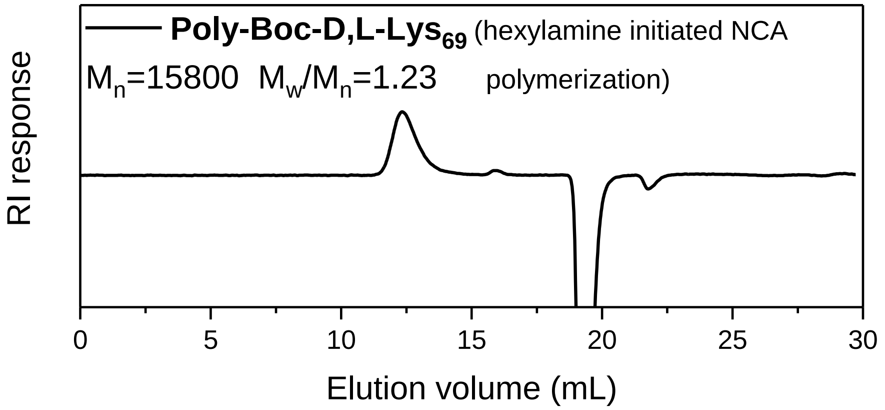

Supplementary Figure 65: GPC traces of poly-Boc-D,L-Lys_{69} (hexylamine initiated NCA polymerization).

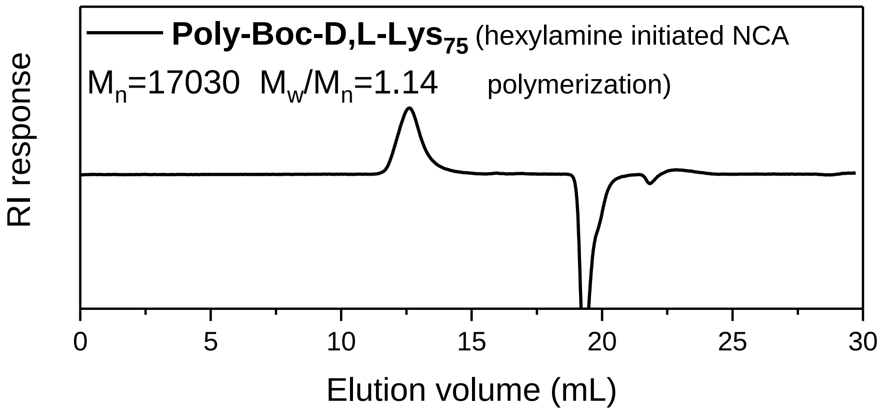

Supplementary Figure 66: GPC traces of poly-Boc-D,L-Lys$_{75}$ (hexylamine initiated NCA polymerization).

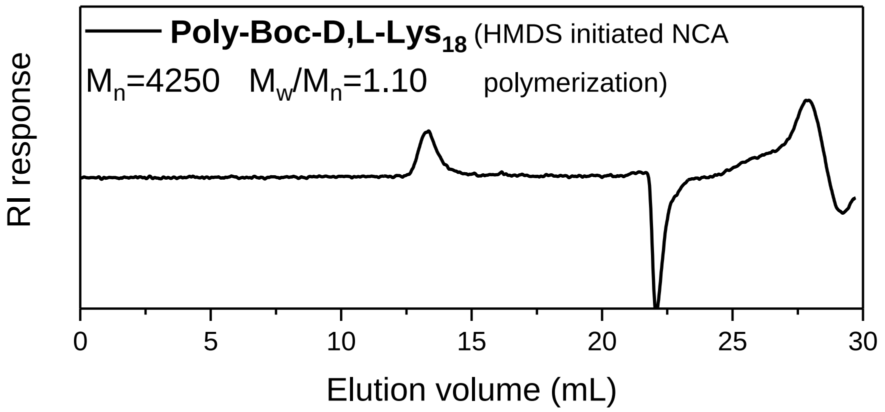

Supplementary Figure 67: GPC traces of poly-Boc-D,L-Lys$_{18}$ (HMDS initiated NCA polymerization).

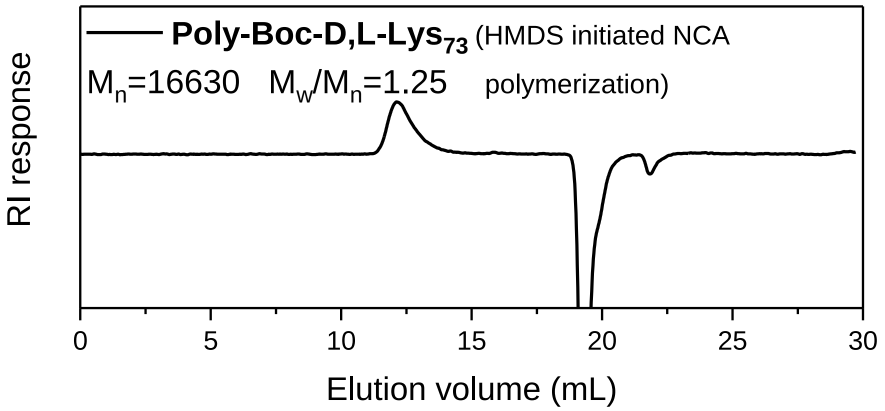

Supplementary Figure 68: GPC traces of poly-Boc-D,L-Lys$_{73}$ (HMDS initiated NCA polymerization).

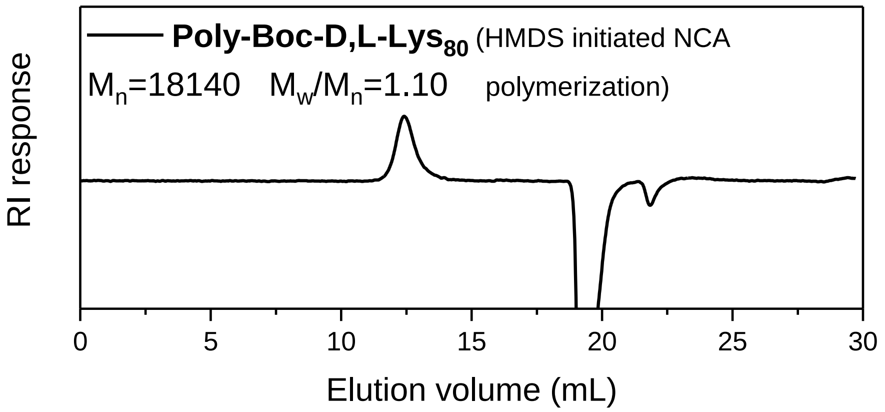

Supplementary Figure 69: GPC traces of poly-Boc-D,L-Lys$_{80}$ (HMDS initiated NCA polymerization).

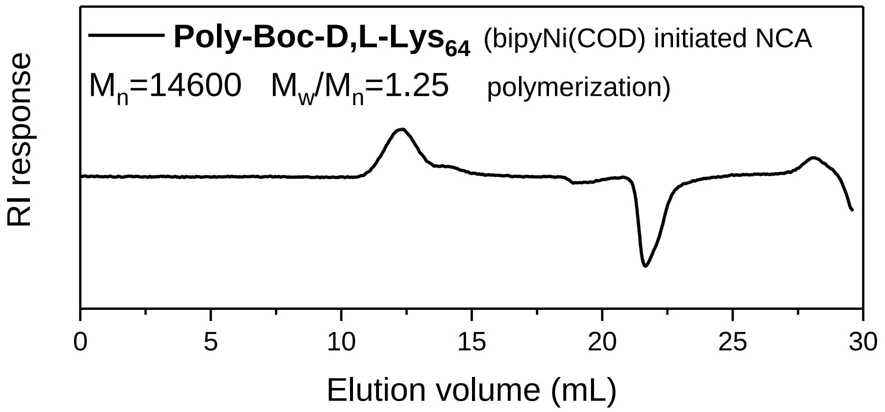

Supplementary Figure 70: GPC traces of poly-Boc-D,L-Lys$_{64}$ (bipyNi(COD) initiated NCA polymerization).

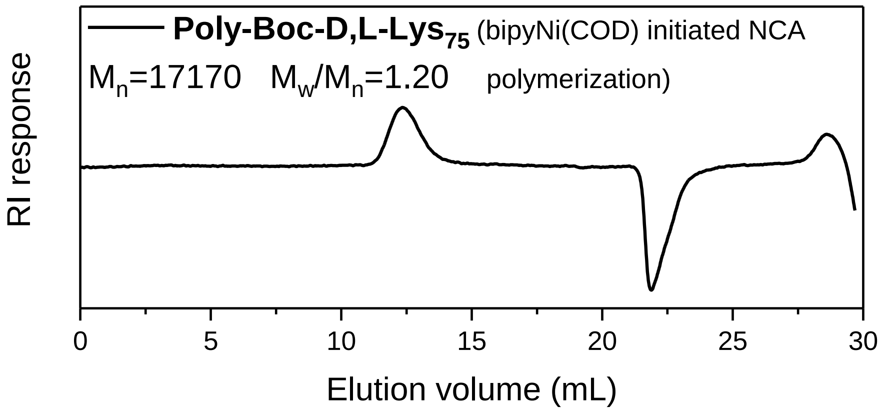

Supplementary Figure 71: GPC traces of poly-Boc-D,L-Lys$_{75}$ (bipyNi(COD) initiated NCA polymerization).

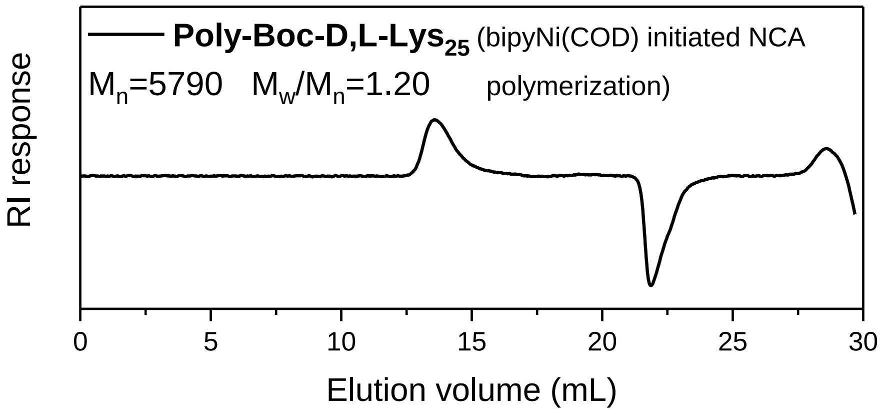

Supplementary Figure 72: GPC traces of poly-Boc-D,L-Lys_{25} (bipyNi(COD) initiated NCA polymerization).

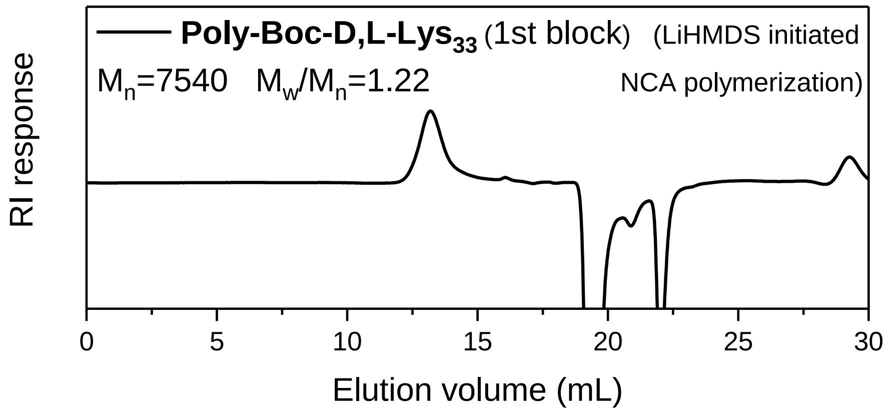

Supplementary Figure 73: GPC traces of poly-Boc-D,L-Lys_{33} (1st block) (LiHMDS initiated NCA polymerization).

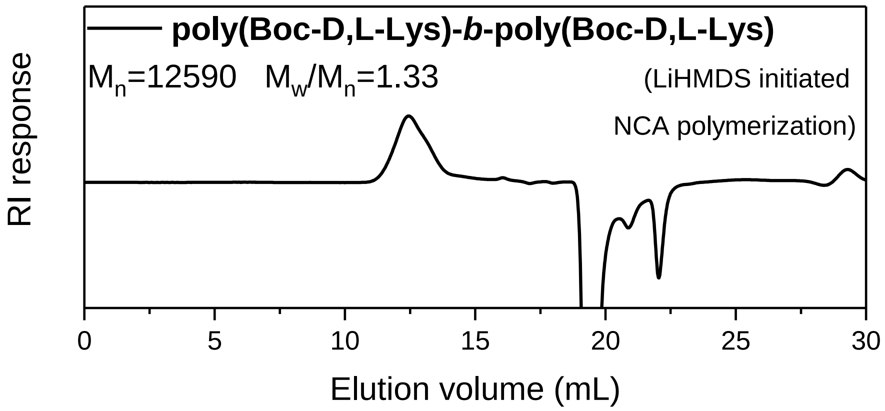

Supplementary Figure 74: GPC traces of poly(Boc-D,L-Lys)-*b*-poly(Boc-D,L-Lys) (LiHMDS initiated NCA polymerization).

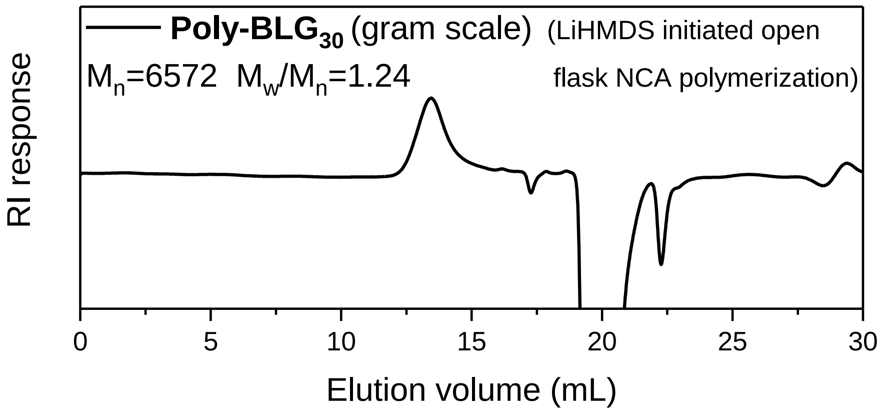

Supplementary Figure 75: GPC traces of poly-BLG_{30} (gram scale) (LiHMDS initiated open flask NCA polymerization).

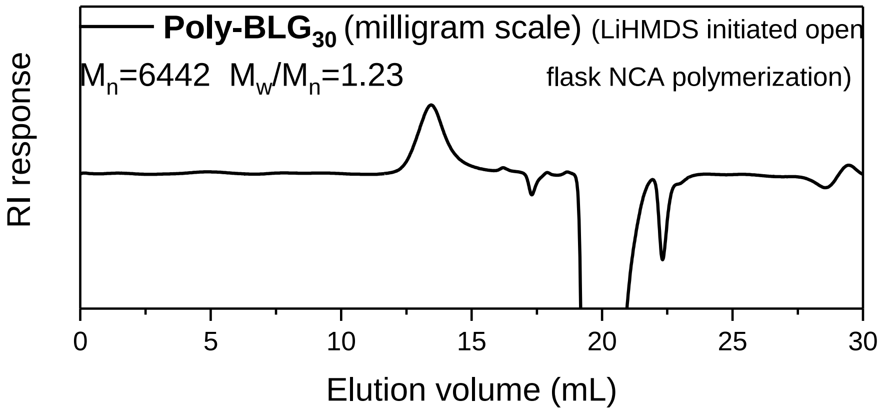

Supplementary Figure 76: GPC traces of poly-BLG_{30} (milligram scale) (LiHMDS initiated open flask NCA polymerization).

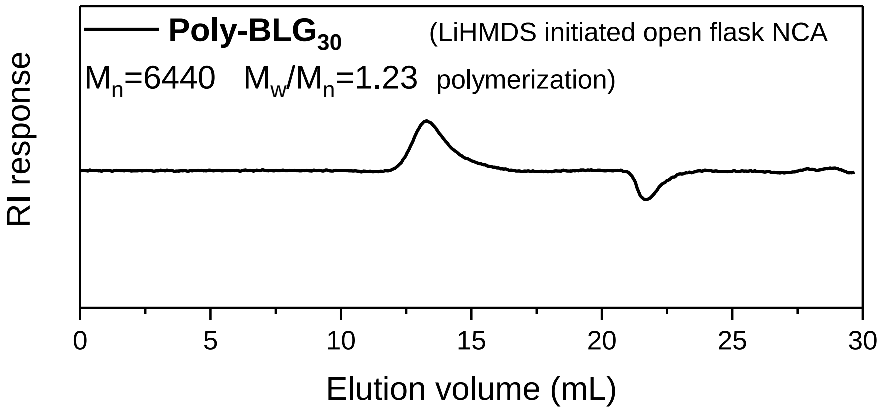

Supplementary Figure 77: GPC traces of poly-BLG_{30} (LiHMDS initiated open flask NCA polymerization).

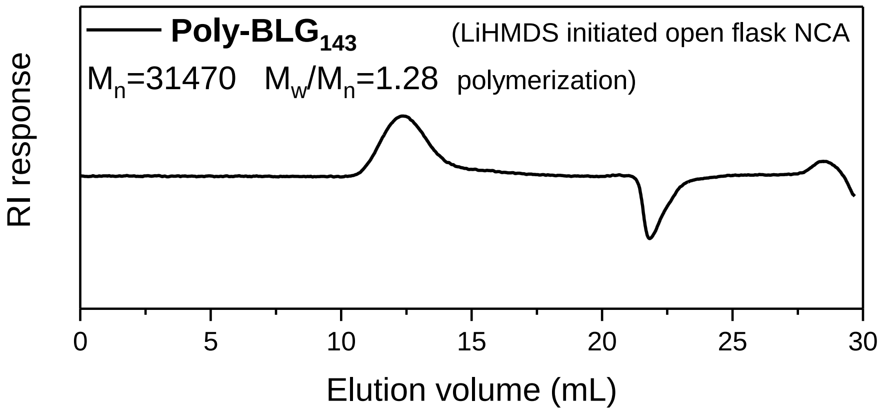

Supplementary Figure 78: GPC traces of poly-BLG$_{143}$ (LiHMDS initiated open flask NCA polymerization).

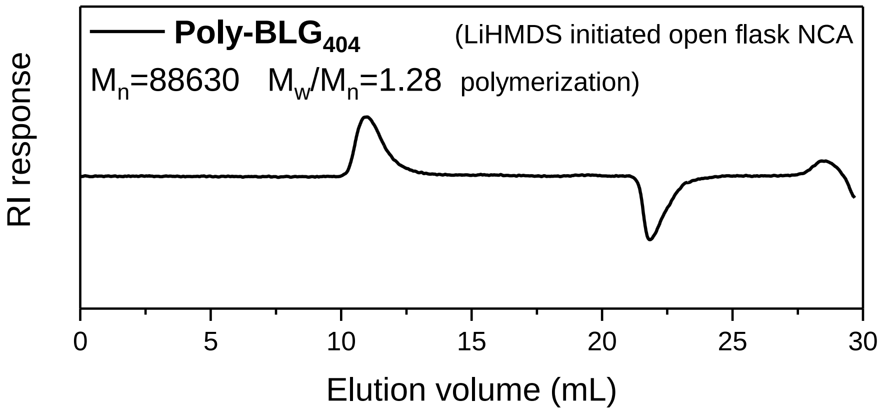

Supplementary Figure 79: GPC traces of poly-BLG$_{404}$ (LiHMDS initiated open flask NCA polymerization).

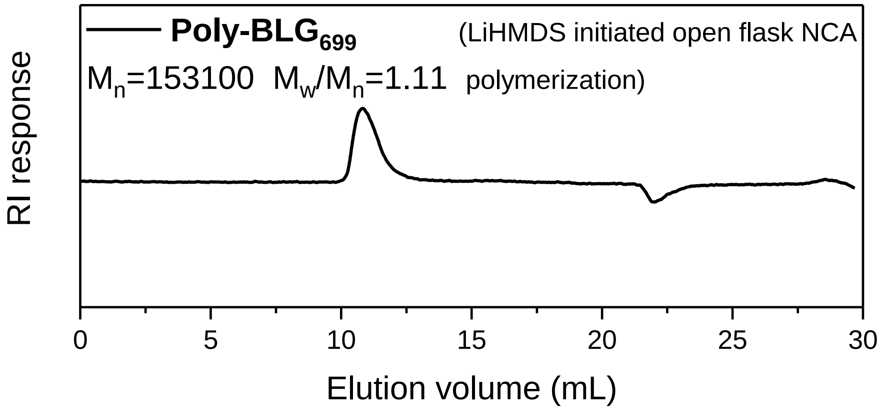

Supplementary Figure 80: GPC traces of poly-BLG$_{699}$ (LiHMDS initiated open flask NCA polymerization).

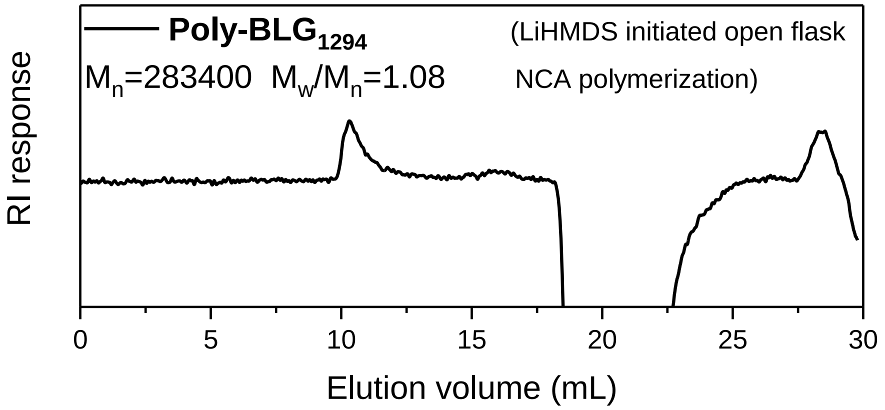

Supplementary Figure 81: GPC traces of poly-BLG$_{1294}$ (LiHMDS initiated open flask NCA polymerization).

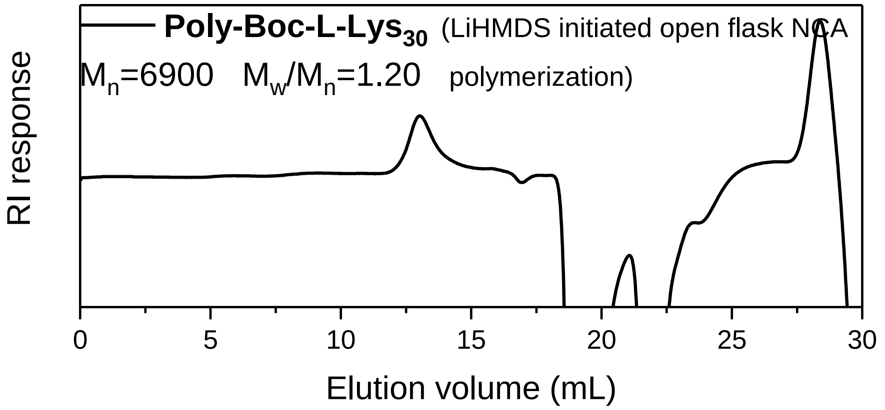

Supplementary Figure 82: GPC traces of poly-Boc-L-Lys$_{30}$ (LiHMDS initiated open flask NCA polymerization).

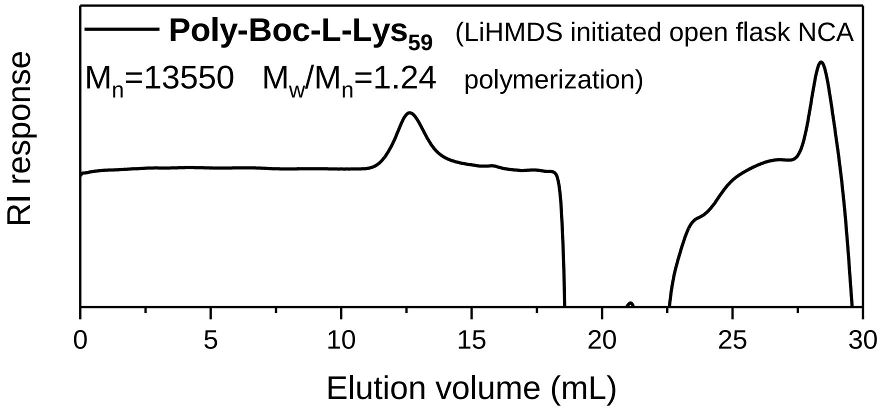

Supplementary Figure 83: GPC traces of poly-Boc-L-Lys$_{59}$ (LiHMDS initiated open flask NCA polymerization).

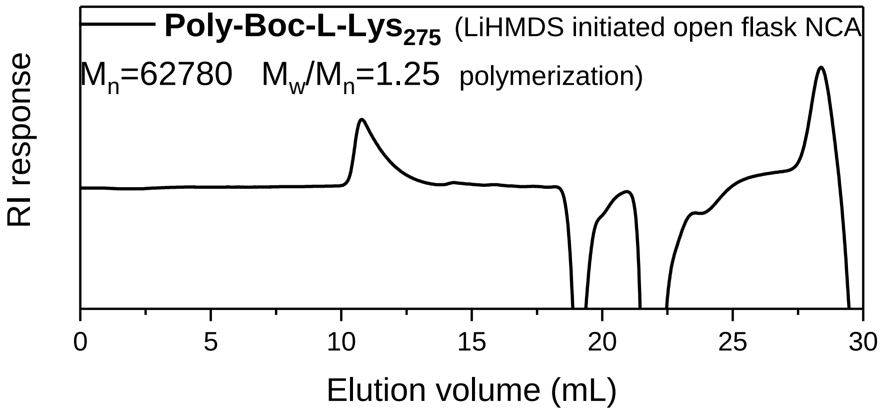

Supplementary Figure 84: GPC traces of poly-Boc-L-Lys$_{275}$ (LiHMDS initiated open flask NCA polymerization).

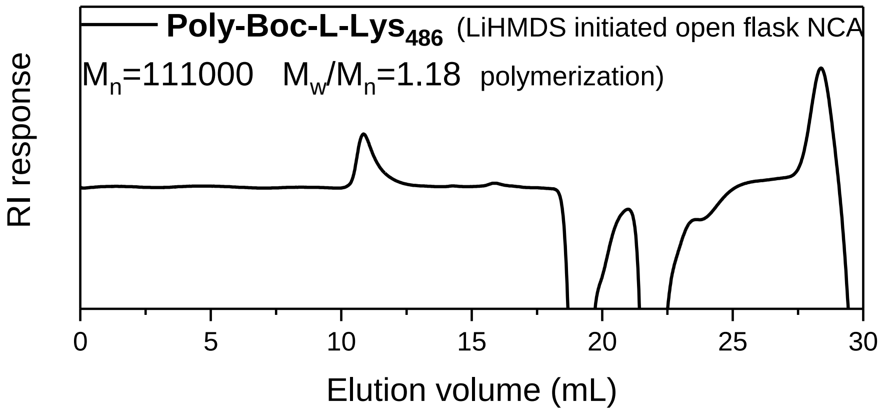

Supplementary Figure 85: GPC traces of poly-Boc-L-Lys$_{486}$ (LiHMDS initiated open flask NCA polymerization).

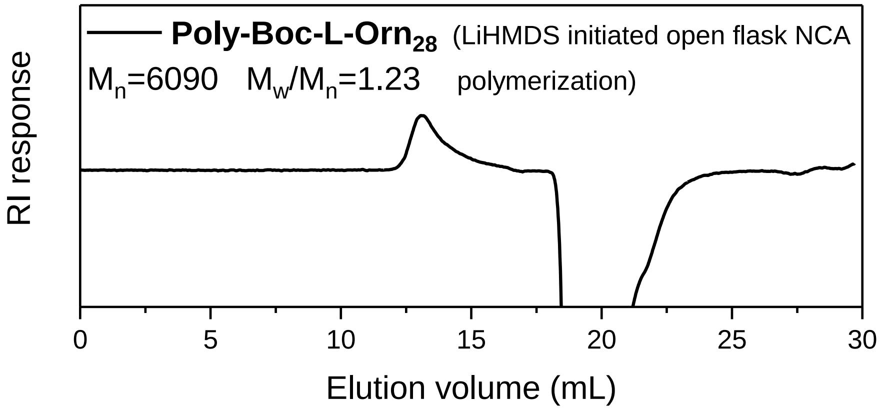

Supplementary Figure 86: GPC traces of poly-Boc-L-Orn$_{28}$ (LiHMDS initiated open flask NCA polymerization).

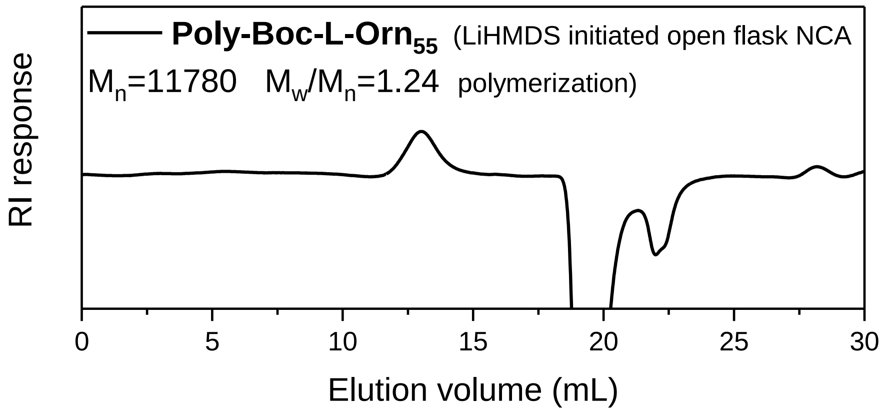

Supplementary Figure 87: GPC traces of poly-Boc-L-Orn_{55} (LiHMDS initiated open flask NCA polymerization).

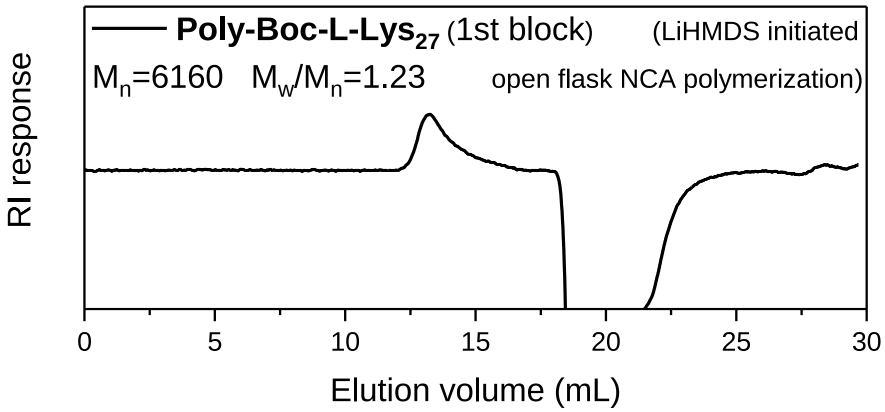

Supplementary Figure 88: GPC traces of poly-Boc-L-Lys_{27} (1st block) (LiHMDS initiated open flask NCA polymerization).

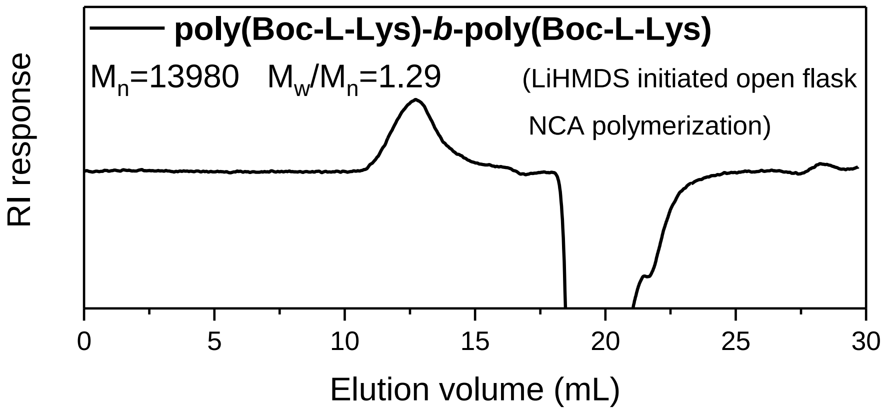

Supplementary Figure 89: GPC traces of poly(Boc-L-Lys)-*b*-poly(Boc-L-Lys) (LiHMDS initiated open flask NCA polymerization).

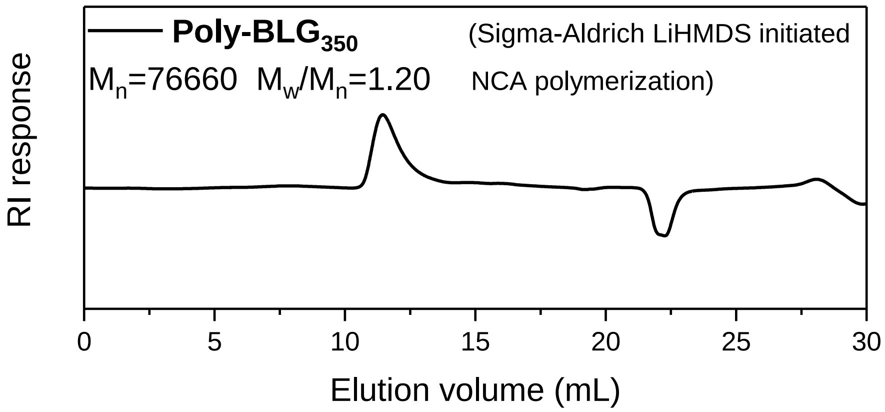

Supplementary Figure 90: GPC traces of poly-BLG_{350} prepared from NCA polymerization, initiaed by LiHMDS (Sigma-Aldrich product lot# SHBH2360V).

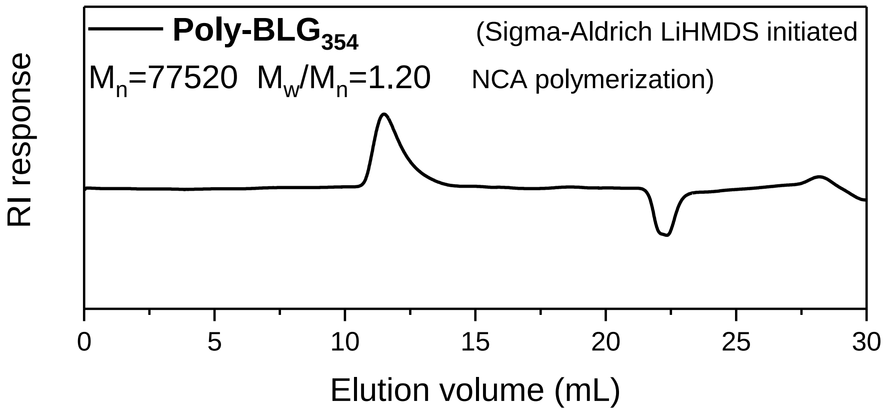

Supplementary Figure 91: GPC traces of poly-BLG_{354} prepared from NCA polymerization, initiaed by LiHMDS (Sigma-Aldrich product lot# SHBH9931).

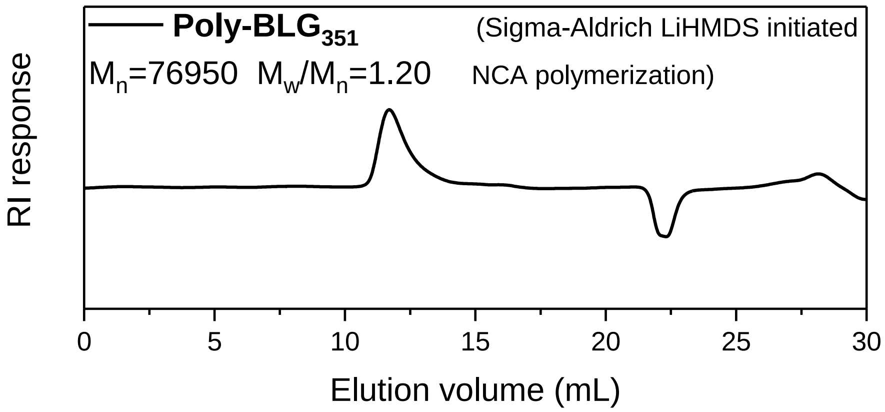

Supplementary Figure 92: GPC traces of poly-BLG_{351} prepared from NCA polymerization, initiaed by LiHMDS (Sigma-Aldrich product lot# SHBH9029).

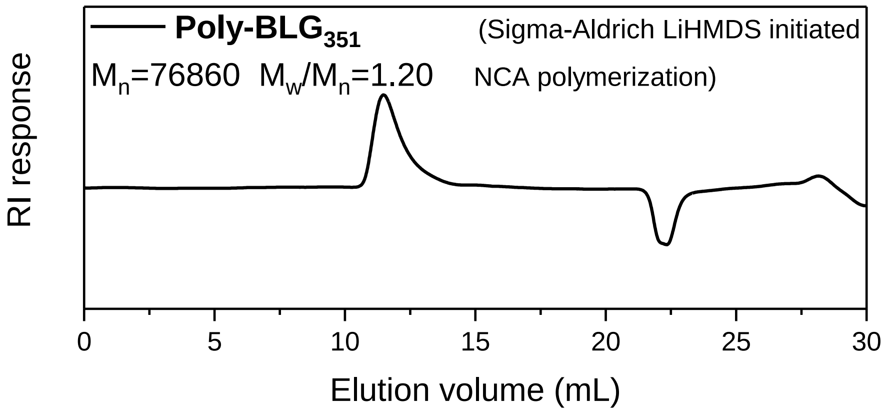

Supplementary Figure 93: GPC traces of poly-BLG_{351} prepared from NCA polymerization, initiaed by LiHMDS (Sigma-Aldrich product lot# SHBH8213).

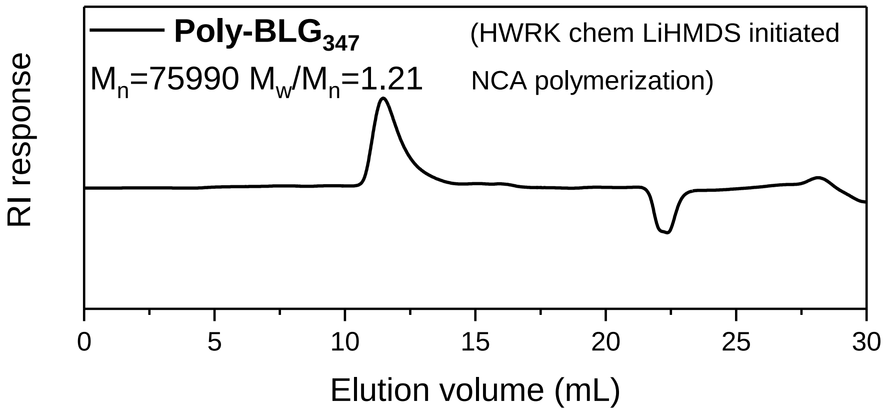

Supplementary Figure 94: GPC traces of poly-BLG_{347} prepared from NCA polymerization, initiaed by LiHMDS (HWRK chem product).

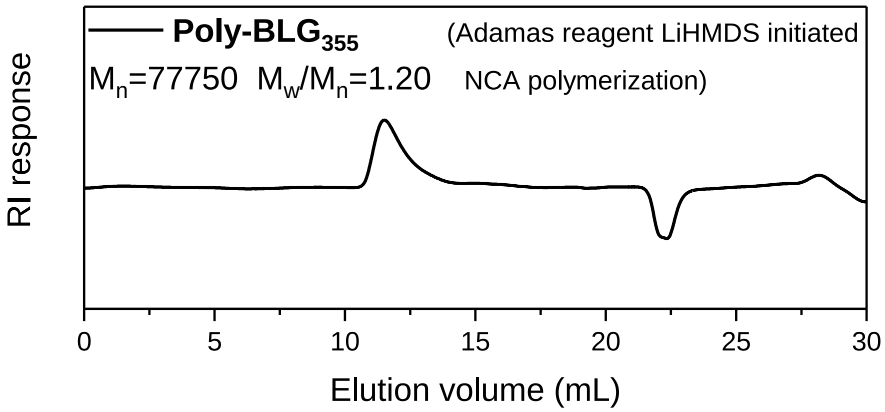

Supplementary Figure 95: GPC traces of poly-BLG_{355} prepared from NCA polymerization, initiaed by LiHMDS (Adamas reagent product).

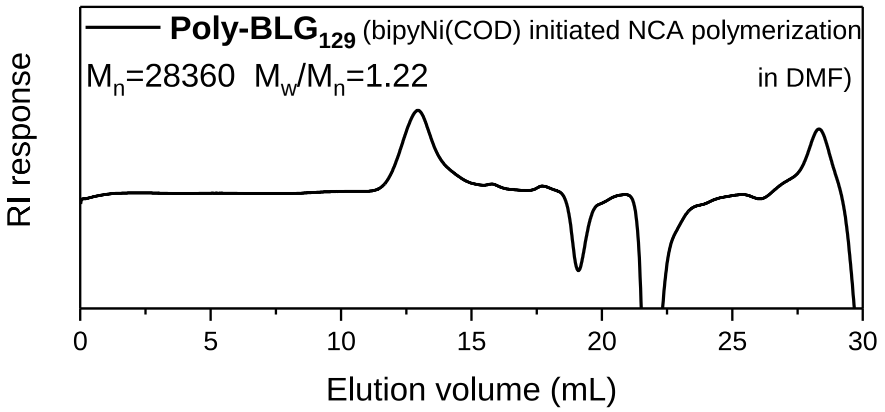

Supplementary Figure 96: GPC traces of poly-BLG_{129} (bipyNi(COD) initiated NCA polymerization in DMF).

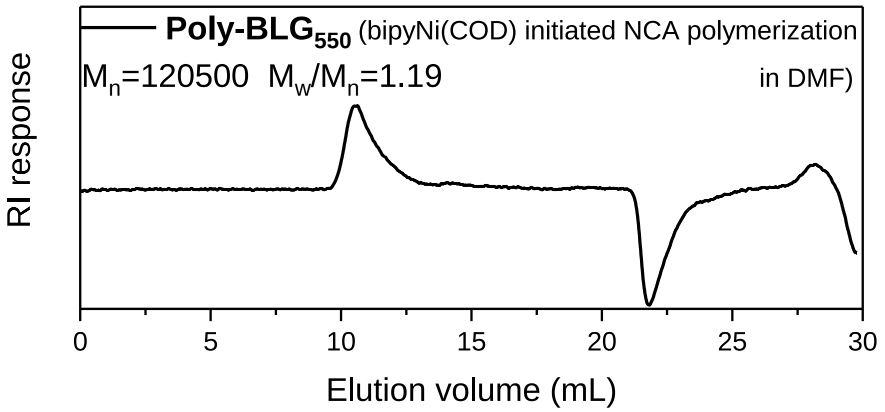

Supplementary Figure 97: GPC traces of poly-BLG_{550} (bipyNi(COD) initiated NCA polymerization in DMF).

Supplementary References

1. Daly, W. H. & Poche, D. The preparation of N-carboxyanhydrides of alpha-amino-acids using bis(trichloromethyl)carbonate. *Tetrahedron Lett.* **29**, 5859-5862 (1988).

2. Bindman, N. A., Bobeica, S. C., Liu, W. R. & Van Der Donk, W. A. Facile removal of leader peptides from lanthipeptides by incorporation of a hydroxy acid. *J. Am. Chem. Soc.* **137**, 6975-6978 (2015).

3. Song, A., Rane, A. A. & Christman, K. L. Antibacterial and cell-adhesive polypeptide and poly(ethylene glycol) hydrogel as a potential scaffold for wound healing. *Acta Biomater.* **8**, 41-50 (2012).

4. Habraken, G. J. M., Peeters, M., Dietz, C. H. J. T., Koning, C. E. & Heise, A. How controlled and versatile is N-carboxy anhydride (NCA) polymerization at 0 °C? Effect of temperature on homo-, block- and graft (co)polymerization. *Polym. Chem.* **1**, 514-524 (2010).

5. Barrett, S. E. et al. An in vivo evaluation of amphiphilic, biodegradable peptide copolymers as siRNA delivery agents. *Int. J. Pharm.* **466**, 58-67 (2014).

6. Lee, H., Park, J. B. & Chang, J. Y. Synthesis of poly(ethylene glycol)/polypeptide/poly(D,L-lactide) copolymers and their nanoparticles. *Polym. Chem.* **49**, 2859-2865 (2011).

7. Vacogne, C. D. & Schlaad, H. Primary ammonium/tertiary amine-mediated controlled ring opening polymerisation of amino acid N-carboxyanhydrides. *Chem. Commun.* **51**, 15645-15648 (2015).

8. Daly, W. H. & Poche, D. The preparation of N-carboxyanhydrides of alpha-amino-acids using bis(trichloromethyl)carbonate. *Tetrahedron Lett.* **29**, 5859-5862 (1988).

9. Semple, J. E., Sullivan, B. & Sill, K. N. Large-scale synthesis of alpha-amino acid-N-carboxyanhydrides. *Synth. Commun.* **47**, 53-61 (2017).

10. Deming, T. J. & Curtin, S. A. Chain initiation efficiency in cobalt- and nickel-mediated polypeptide synthesis. *J. Am. Chem. Soc.* **122**, 5710-5717 (2000).

11. Jia, X. G., Guo, P., Duan, J. C. & Shu, X. Z. Dual nickel and Lewis acid catalysis for cross-electrophile coupling: the allylation of aryl halides with allylic alcohols. *Chem. Sci.* **9**, 640-645 (2018).

12. Kricheldorf, H. R., Von Lossow, C. & Schwarz, G. Primary amine and solvent-induced polymerizations of L- or D,L-phenylalanine N-carboxyanhydride. *Macromol. Chem. Phys.* **206**, 282-290 (2005).

13. Kricheldorf, H. R., Von Lossow, C. & Schwarz, G. Cyclic polypeptides by solvent-induced polymerizations of alpha-amino acid N-carboxyanhydrides. *Macromolecules* **38**, 5513-5518 (2005).

14. Kramer, J. R. & Deming, T. J. General method for purification of alpha-amino acid-N-carboxyanhydrides using flash chromatography. *Biomacromolecules* **11**, 3668-3672 (2010).
